# Fecal microbiome profiles of neonatal dairy calves with varying severities of gastrointestinal disease

**DOI:** 10.1371/journal.pone.0262317

**Published:** 2022-01-04

**Authors:** Giovana S. Slanzon, Benjamin J. Ridenhour, Dale A. Moore, William M. Sischo, Lindsay M. Parrish, Sophie C. Trombetta, Craig S. McConnel

**Affiliations:** 1 Department of Veterinary Clinical Sciences, College of Veterinary Medicine, Washington State University, Pullman, Washington, United States of America; 2 Department of Mathematics and Statistical Science, College of Science, University of Idaho, Moscow, Idaho, United States of America; Michigan State University, UNITED STATES

## Abstract

Gastrointestinal disease (GI) is the most common illness in pre-weaned dairy calves. Studies have associated the fecal microbiome composition with health status, but it remains unclear how the microbiome changes across different levels of GI disease and breeds. Our objective was to associate the clinical symptoms of GI disease with the fecal microbiome. Fecal samples were collected from calves (n = 167) of different breeds (Holstein, Jersey, Jersey-cross and beef-cross) from 4–21 d of age. Daily clinical evaluations assessed health status. Calves with loose or watery feces were diagnosed with diarrhea and classified as bright-sick (BS) or depressed-sick (DS) according to behavior. Calves with normal or semiformed feces and no clinical illness were classified as healthy (H). One hundred and three fecal samples were obtained from consistently healthy calves and 64 samples were from calves with diarrhea (n = 39 BS; n = 25 DS). The V3-V4 region of 16S rRNA gene was sequenced and analyzed. Differences were identified by a linear-mixed effects model with a negative binomial error. DS and Jersey calves had a higher relative abundance of *Streptococcus gallolyticus* relative to H Holstein calves. In addition, DS calves had a lower relative abundance of *Bifidobacterium longum* and an enrichment of *Escherichia coli*. Species of the genus *Lactobacillus*, such as an unclassified *Lactobacillus*, *Lactobacillus reuteri*, and *Lactobacillus salivarius* were enriched in calves with GI disease. Moreover, we created a model to predict GI disease based on the fecal microbiome composition. The presence of *Eggerthella lenta*, *Bifidobacterium longum*, and *Collinsella aerofaciens* were associated with a healthy clinical outcome. Although lactobacilli are often associated with beneficial probiotic properties, the presence of *E*. *coli* and *Lactobacillus* species had the highest coefficients positively associated with GI disease prediction. Our results indicate that there are differences in the fecal microbiome of calves associated with GI disease severity and breed specificities.

## Introduction

Neonatal calf gastrointestinal (GI) disease, often simply diagnosed as diarrhea, is considered one of the major health challenges in the dairy industry. This disorder has a considerable economic impact on dairy farm operations and is responsible for causing substantial economic losses in cattle herds worldwide [1]. The National Animal Health Monitoring System (NAHMS) reported that diarrhea is the most common disease affecting pre-weaned heifers: 21% of pre-weaned heifers were diagnosed with GI problems, and 76% of these calves were treated with antibiotics [2]. Economic losses associated with GI disease include direct costs of calf losses, treatment costs (e.g., antibiotics and electrolytes), time spent caring for the affected animal and long-term effects on performance as a result of reduced growth rates [3,4]. In addition, GI disease represents a threat to animal welfare [5] due to the presence of physical and psychological stressors [6].

GI disease is a multifactorial pathophysiologic process that is complex to diagnose, prevent, and treat. On-farm calf caretakers and even veterinary personnel may lack the knowledge and on-farm tools to correctly identify phenotypes and different gradients of severity. Although this disease can manifest clinically as loose feces without other obvious clinical symptoms, it often presents a constellation of clinical signs including variable degrees of dehydration, electrolyte imbalance, and metabolic acidosis [7]. However, diagnoses based on fecal scores alone lack information regarding the actual biological adaptations that the GI tract undergoes during the onset of diarrhea [8]. Importantly, accurate and specific diagnoses that help in the understanding of these physiological and microbial changes can direct dairy producers and veterinarians to precisely administer therapies and implement practices that prevent new cases from occurring [9,10].

Comprehension of GI disease complexities should be the foundation guiding the prevention and control of calf diarrhea [11]. This includes an understanding of the interactions between microbiome composition and the development of disease [12], as well as the influence of host genetics on the fecal microbiome [13,14]. It has been suggested that the microbial colonization of the GI tract of neonatal calves begins even before birth, and it changes rapidly during the first weeks of life [15,16]. Furthermore, host-microbe interplay has been implicated in regulating different aspects of health [17], and for that reason studies are exploring how GI disorders are linked with alterations in the fecal microbial community structure of dairy calves [12,18]. However, without additional insight into disease phenotypes including breed-specific differences in the progression of disease and microbial signatures across different gradients of GI disease, it is difficult to 1) clarify the full diversity of underlying pathophysiologic processes that manifest as diarrhea, 2) implement appropriate preventative options, 3) accurately assess the associated costs, and 4) evaluate the efficacy of therapies including antimicrobials.

Therefore, as an initial step toward a better understanding of GI disease phenotypes this study utilized clinical assessments and fecal microbial community evaluations to test the hypothesis that fecal microbiome profiles differ between neonatal dairy calves with and without diarrhea. With this as a foundation, fecal microbiomes were compared across breeds and GI disease gradients to understand diarrhea complexities with more precision. We hypothesized that the fecal microbiome of calves with systemic clinical signs associated with GI disease would differ from the fecal microbiome of calves with diarrhea as the only symptom of GI disease. Moreover, we compared the fecal microbiomes of calves across different breeds. Therefore, we further hypothesized that there are breed specific characteristics of the microbiome correlated with varying GI disease severity. This study ultimately aimed to further the development of effective mechanisms to identify and treat GI disease through the practical application of bioinformatic data to animal health.

## Materials and methods

### Ethics statement

The research protocol was reviewed and approved by the Institutional Animal Care and Use Committee of Washington State University (ASAF#6414).

### Study design and calf enrollment

This field-based cohort study was conducted on a commercial calf ranch in the western U.S. in 2019. All 360 calves that arrived on the calf-ranch between 20–22 May, in addition to all 269 calves that arrived on the calf-ranch between 14–16 July were enrolled in the study. The ranch housed approximately 25,000 Holstein, Jersey, Jersey-cross (Jersey x Holstein), and crossbred (Jersey x Angus) calves from multiple dairies through 200 days of age. Colostrum was fed to the calves at the dairy of birth and the calves arrived at the ranch at <1 day of age. Calves did not spend more than two hours on transport. In order to assess transfer of passive immunity, blood samples were obtained from one-day-old calves via jugular venipuncture to measure total serum protein (TSP) using a calibrated refractometer [19].

**Fig 1:**
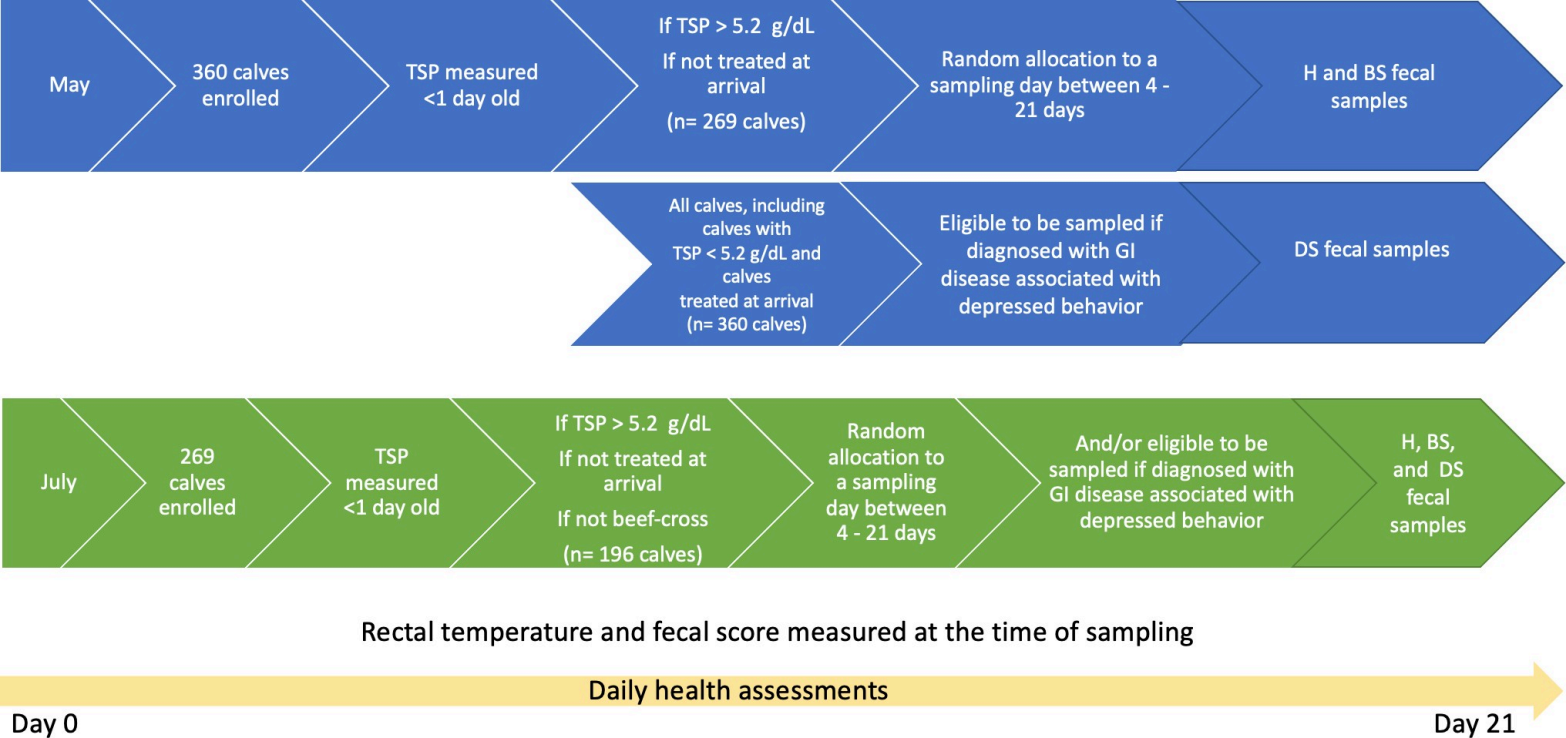
Graphical timeline of calf enrollment and data collection. Calves with evidence of diarrhea were classified as either bright sick (BS) based on diarrhea alone, or depressed sick (DS) if diarrhea was aligned with inappetence or changes in behavior. Calves with fecal scores <3 were classified as consistently healthy (H) if they did not elicit any treatment for either diarrhea, respiratory disease or other health events during the period of 21 days after arrival. TSP = total serum protein.

Calves were sampled either at a pre-determined time based on age or when diagnosed with diarrhea associated with depressed behavior. Using a random number generator for eligible calves, calves with TSP levels ≥5.2 g/dL were allocated to a sampling day between four to 21 days of age to have fecal samples (~25–250 g) collected after the morning feeding. Calves enrolled in May with low TSP (<5.2 g/dL) or that were treated (e.g., antimicrobial or anti-inflammatory drugs) during the first 3 days of age were not eligible to be sampled unless diagnosed with systemic clinical changes related to diarrhea. During the second enrollment period, beef-cross calves were unavailable to be sampled and it was determined that no calves were eligible to be sampled if they had low TSP (<5.2 g/dL) or if they were treated (e.g., antimicrobial or anti-inflammatory drugs) during the first three days of age (Fig 1). During the first three weeks of life, calves that manifested clinical signs of diarrhea associated with depressed behavior were sampled at the time of diagnosis as a comparison against healthy calves and calves with diarrhea but no other observed clinical symptoms. Therefore, repeated sampling of an individual occurred only when they had a pre-determined sampling age and subsequently developed diarrhea associated with depressed behavior. However, in those cases the previous sample was discarded and only the sample at the time of systemic diagnosis was analyzed.

### On farm animal care

On-farm personnel were responsible for all primary care of the calves including feeding, cleaning, watering and bedding maintenance. The calves were housed in adjacent individual hutches with no direct contact between calves. Upon arrival, on-farm personnel performed dehorning using a caustic paste (Dr. Naylor Dehorning Paste, H.W. Naylor Co. Inc., Morris NY, USA) and sprayed the calves with a fly control spray (Ultra-Boss Pour-on Insecticide, Intervet Inc., Merck Animal Health, Omaha NE, USA). An enteric clostridial vaccine (Ultrabac CD, Zoetis Inc., Kalamazoo MI, USA) and a vitamin complex with selenium and vitamin E (MU-SE, Intervet Inc., Merck Animal Health, Omaha NE, USA) were also administered on arrival. An intranasal viral respiratory vaccine (Vista Once SQ, Intervet Inc., Merck Animal Health, Omaha NE, USA) was administered at 2 days of age and a booster was administered at 20 days of age.

Calves enrolled in May 2019, were fed 2 liters of a custom milk blend in a bottle twice daily, from one day of age to 52 days. Calves enrolled in July received 2 liters of the same custom milk blend twice daily, from one day of age to 15 days of age and after this age, calves were fed an additional two liters of milk offered in the middle of the day until 52 days of age. The milk blend consisted of pasteurized waste milk and milk replacer and was targeted for an optimal composition of 13% solids, 22–24% fat, and 28% protein. Under the oversight of a licensed veterinarian, milk medicated with neomycin and oxytetracycline (Neo-Oxy 100/100 MR, PharmGate, Omaha NE, USA) was fed at the labeled dose (10 mg/lb body weight/day) to all calves between five to 12 days of age throughout the study period to combat ongoing diarrhea problems. No pre or probiotics were added to the milk. A customized electrolyte (one liter/calf) also was offered after the afternoon feeding during this same risk period from five to 12 days of age. Fresh water was available between milk feedings. A grain mix consisting of pellets, molasses, and whole corn was offered from day three of age and steadily increased to approximately 2.25 kg by day 30 with free choice thereafter.

### Data collection

#### Health assessment

Twice daily evaluations were conducted prior to and at the time of each feeding (6:00 am and 4:00 pm) by a PhD candidate and WSU veterinarian, assessing health status and clinical disease severity from the day of arrival through the completion of the follow-up period of 21 days. Due to inconsistencies in fecal appearance within the calf hutches and an inability to observe each calf defecate at the time of all clinical evaluations, fecal consistency scores only were recorded at the time of fecal sampling. Rectal temperature utilizing a digital thermometer and assessments of fecal consistency (1 = well-formed; 2 = semi-formed; 3 = loose; 4 = watery) were recorded for all calves at the time of sampling. Calves with fecal scores of 3 or 4 (3 = loose and 4 = watery) [20] were diagnosed with diarrhea and classified based on clinical signs primarily focused on behavior (bright for when the calf was responsive and active; or depressed for when the calf was considered dull, weak, or unresponsive), and milk intake (good appetite; did not finish the milk or did not take any of what was offered; required orogastric intubation). Based on these qualifiers, calves with evidence of GI disease were classified as either bright sick (**BS**) based on diarrhea alone, or depressed sick (**DS**) if diarrhea was aligned with inappetence or changes in behavior. A matrix incorporating demeanor, mobility, appetite, hydration status based on ocular recession, fecal scores and rectal temperature of DS calves can be found at S1 File. Calves with fecal scores <3 were classified as consistently healthy (**H**) if they did not elicit any treatment for either diarrhea, respiratory disease or other health events during the period of 21 days after arrival. Calves with clinical signs such as inappetence, dehydration (ocular recession ≥3 mm), recumbency (sternal or lateral), and diarrhea were treated by farm personnel following farm protocols.

#### Fecal samples

Fecal samples were collected in sterile sampling bags (Thermo Fisher Scientific, USA) and immediately placed in a cooler with ice packs, until they could be frozen (-20°C) within five hours after sampling. The samples were transferred to Washington State University on dry ice and stored for approximately 30 days in a -20°C freezer until further processing within the Field Disease Investigation Unit laboratory.

#### Fecal dry matter

Fecal samples were assessed for total dry matter by weighing out 1 gram of raw sample and drying the sample in an incubator at 37°C for 72 hours to remove moisture. Percent dry matter was calculated based on the difference between dry weight and wet weight.

### Amplification and sequencing of bacterial 16S rRNA gene

At the time of processing, fecal samples were thawed, mixed, and placed (1 g) into fecal DNA/RNA shield fecal collection tubes (Zymo Research, Irvine, CA). Samples were sent to be further processed and analyzed through the ZymoBIOMICS Service: Targeted Metagenomic Sequencing (Zymo Research, Irvine, CA). The ZymoBIOMICS-96 MagBead DNA Kit (Zymo Research, Irvine, CA) was used to extract the DNA from fecal samples. The DNA samples were prepared for targeted sequencing with the *Quick*-16 NGS Library Prep Kit (Zymo Research, Irvine, CA). The ZymoBIOMICS Microbial Community Standard (Zymo Research, Irvine, CA) was used as a positive control for each DNA extraction and targeted library preparation. Negative controls (i.e., blank extraction control, blank library preparation control) were included to assess the level of bioburden carried by the wet-lab process. The V3–V4 region (primers 341F-806R) of the 16S rRNA gene was amplified with Quick-16S Primer Set V3–V4 (Zymo Research, Irvine, CA). The final amplicon sizes, including primers, was ~350bp and ~460bp, respectively. The final library was sequenced on Illumina MiSeq with a v3 reagent kit (600 cycles). The sequencing was performed with >10% PhiX spike-in.

### Statistical analysis

Fastq files generated by Illumina MiSeq (2x300bp) amplicon reads were processed using the package dada2 [21] in R program 4.0.0 (R Project for Statistical Computing). Unique sequences were classified to species levels by SILVA 123 ribosomal RNA database [22] using SPINGO [23]. If sequences were identified as ambiguous by SPINGO, Ribosomal Database Project 11, University of Illinois in Urbana, IL [24], was used for taxonomy identification, and if sequences were still undefined, the next approach adopted was Basic Local Alignment Search Tool (BLAST) [25] available from the National Center for Biotechnology Information [26].

The final amplicon sequence variant (ASV) table was filtered via mutual information-based microbiome analysis that accounted for information loss [27]. The results of the filtered taxonomy ASV table were analyzed using the vegan package [28] in R program to group communities on the basis of similarities and differences in composition. Relative abundance data were calculated based on the number of sequences reads and total reads per sample. The chisq.test function in R’s stats package was used to perform Pearson’s chi-squared test with the objective to determine if the microbiome composition differs across health states. To explore and visualize dissimilarities between individuals and groups, Beta diversity was analyzed using the ordinate function in R’s phyloseq package [29] to create a Principal Coordinates Analysis (PCoA  =  Multidimensional scaling, MDS) based on the normalized number of reads in each sample, using median sequencing depth and the modified Gower distance (altGower) [30].

The linear mixed-effects model (LME) with a negative binomial error was fitted using the glmer.nb function of lme4 package [31], based on the log of total read counts of each sample, with the objective to identify the relationship between specific microbial organisms and disease severity. The primary explanatory variable of interest was clinical outcomes (healthy, bright sick, or depressed sick). Other variables included age in days, breed, enrollment period, and a variable indicating if the calf was receiving medicated milk or not at the time of sampling. In addition, the source farm on which the calf was born was considered a random effect in the model. We used Akaike information criterion as a basis for model selection. In order to identify differences in the microbiome across breeds, pairwise contrasts for each calf breed were calculated based on the estimated marginal means of the model using the functions emmeans and emmip, from the package emmeans. Prediction of disease was calculated using a binomial family to fit a generalized linear regression model with lasso regularization, using the glmnet function of glmnet package [32]. The explanatory variables were a matrix model of the number of sequences reads and total reads per sample, breed, age at sampling, and source farm, and the outcome variable was calf’s health (diarrhea incidence = TRUE or no diarrhea incidence = FALSE). The cv.glmnet function of glmnet package was used to perform cross-validation and calculate lambda.1se, the most regularized model such that error is within one standard error of the minimum. R code and its output can be found in S1 Appendix.

## Results

### Sample population

In 2019 a total of 360 calves were enrolled in the study from 20–22 May, and 269 were enrolled between 14–16 July. Calves originated from 28 different farms. A total of 167 of the enrolled calves were sampled. Of the 167 samples, 98 and 69 calves were sampled from the first and second enrollment cohorts, respectively. Both datasets were combined for analysis. Of those calves that were sampled, 103 fecal samples were obtained from consistently healthy (**H**) calves and 64 samples were from calves with diarrhea (n = 39 bright sick [**BS**]; n = 25 depressed sick [**DS**]). Fecal samples were obtained from calves representing all of the different breeds (farm records based on breeding records) at the ranch (Holstein n = 78, Jersey n = 55, Jersey-cross [Holstein x Jersey] n = 17 and beef-cross n = 17) as well as different ages (range = 4–21 days; average = 9 days; SD = 3.3 days). The samples distribution per age, breed and health state can be found in S1 Table. All DS cases were observed only during the 4–15 day period. The average fecal dry weight (median and interquartile range, IQR) of H samples was 26.5% (26.3%, IQR 8.7%), BS samples averaged 16.9% (16%, IQR 8.9%), and the average of DS samples was 14.7% (13%, IQR 10.8%). Two of the 25 DS samples collected were from calves enrolled in May with TSP equal to 5 g/dL. Furthermore, two additional DS samples were from calves enrolled in May that were medically treated at arrival with ceftiofur crystalline free acid and flunixin meglumine by farm personnel according to the dosage indicated by the label. These calves were sampled 4 and 7 days later, respectively, at the time of the diagnosis of diarrhea associated with depressed behavior.

### Calves and management practices

During the first period of sample collection, 20.5% (74/360) of calves were treated for either diarrhea, respiratory disease, or other health events during the first 21 days of age. More than half of the treatments targeted diarrhea (57%; 42/74), 28% targeted respiratory symptoms (21/74), and 15% of treatments were for other reasons such as joint problems or septicemia (11/74). The median TSP of the calves was 5.8 g/dL (n = 360). Seventy-three calves (20%) had TSP <5.2 g/dL but only two of those were included in the analysis as described above.

During the second sampling period, 18% (48/269) of calves were treated for either diarrhea, respiratory disease, or other health events during the first 21 days of age. Over half the treatments targeted diarrhea (79%; 38/48), 10% targeted respiratory symptoms (5/48), and 10% of treatments were for other reasons (5/48). No beef-cross calves were treated for diarrhea during this period. Of those 269 calves, 48 calves were beef-cross and were not sampled nor had their TSP measured. The median TSP of calves was 6.0 g/dL (n = 221). Nineteen calves (8.6%) had TSP <5.2 g/dL and were not eligible to be sampled (Table 1).

**Table 1:** Number of enrolled and sampled calves, TSP measurements, and diarrhea incidence by breed.

Enrollment period	Number of enrolled calves	Total number of calves sampled	Number of calves sampled while receiving medicated milk	Median TSP (g/dL) and IQR	Number of calves treated for diarrhea
**May**					
Holstein	194	54 (n = 43 H, n = 5 BS, n = 6 DS)	39 (n = 31 H, n = 5 BS, n = 3 DS)	5.7 (0.6)	14 (7%)
Jersey	88	23 (n = 8 H, n = 6 BS, n = 9 DS)	21 (n = 6 H, n = 6 BS, n = 9 DS)	5.8 (0.6)	20 (23%)
Jersey-cross	30	4 (n = 2 H, n = 1 BS, n = 1 DS)	4 (n = 2 H, n = 1 BS, n = 1 DS)	5.8 (0.5)	6 (20%)
beef-cross	48	17 (n = 15 H, n = 2 DS)	13 (n = 11 H, n = 2 DS)	5.9 (0.6)	2 (4%)
**July**					
Holstein	136	24 (n = 14 H, n = 7 BS, n = 3 DS)	21 (n = 12 H, n = 6 BS, n = 3 DS)	6.0 (0.6)	26 (19%)
Jersey	53	32 (n = 14 H, n = 15 BS, n = 3 DS)	25 (n = 11 H, n = 11 BS, n = 3 DS)	6.0 (0.7)	8 (15%)
Jersey-cross	32	13 (n = 7 H, n = 5 BS, n = 1 DS)	12 (n = 6 H, n = 5 BS, n = 1 DS)	6.0 (0.6)	4 (12.5%)
beef-cross	48	0	0	-	0

TSP = total serum protein.

IQR = interquartile range.

H = healthy, BS = bright sick, and DS = depressed sick calves.

### Fecal microbiota composition among different breeds of calves with and without GI disease

A total of 431 ASVs were identified in the 167 fecal samples. We first determined an appropriate threshold for mutual information scores in our microbial network using an expected power-law distribution; the best-fit power-law distribution (R^2^ = 0.89) occurred when mutual information scores (network edges) below 0.4 were dropped. After dropping these edges from the network, we determined the number of ASVs to retain for analysis by sequentially dropping ASVs from lowest abundance to highest abundance, and checking afterward if the information loss in the system was significant [27]. This process indicated that we could remove 76% of the sequencing data without significant information loss (p > 0.05 following Benjamini-Hochberg correction). These methods resulted in 57 “significant” ASVs which were included in subsequent analyses; the remaining 374 “insignificant” ASVs were grouped into an “other” category.

**Fig 2:**
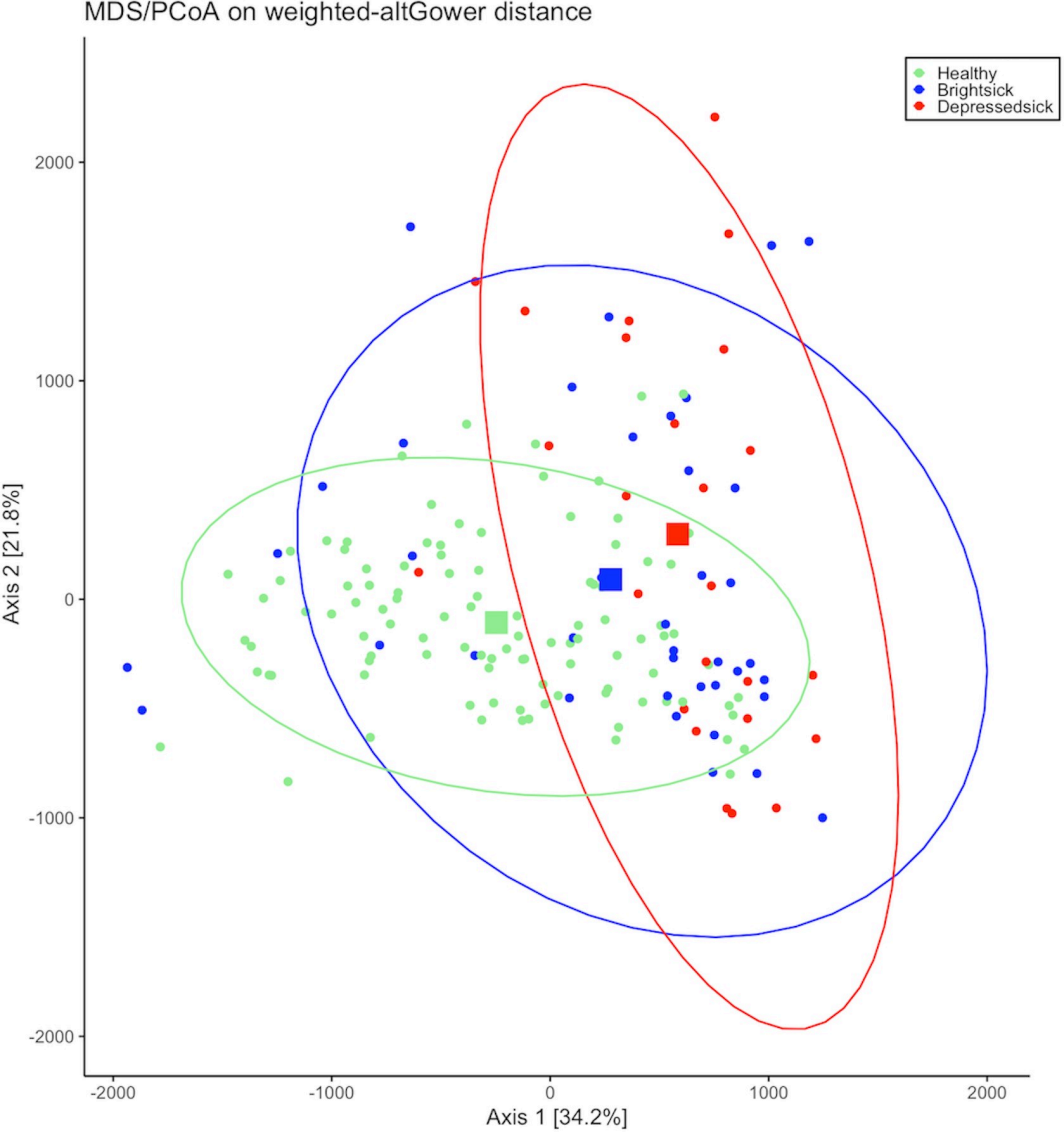
Principal coordinates analysis (PCoA) based on Gower distances grouped by health status. Proportion of variance explained by each principal coordinate axis is denoted in the corresponding axis label. The squares indicate the centroids for each group. The ellipses represent a 95% confidence interval calculated based on a t-distribution. Calves with evidence of diarrhea were classified as either bright sick based on diarrhea alone, or depressed sick if diarrhea was aligned with inappetence or changes in behavior. Calves with fecal scores <3 were classified as consistently healthy if they did not elicit any treatment for either diarrhea, respiratory disease or other health events during the period of 21 days after arrival.

**Fig 3:**
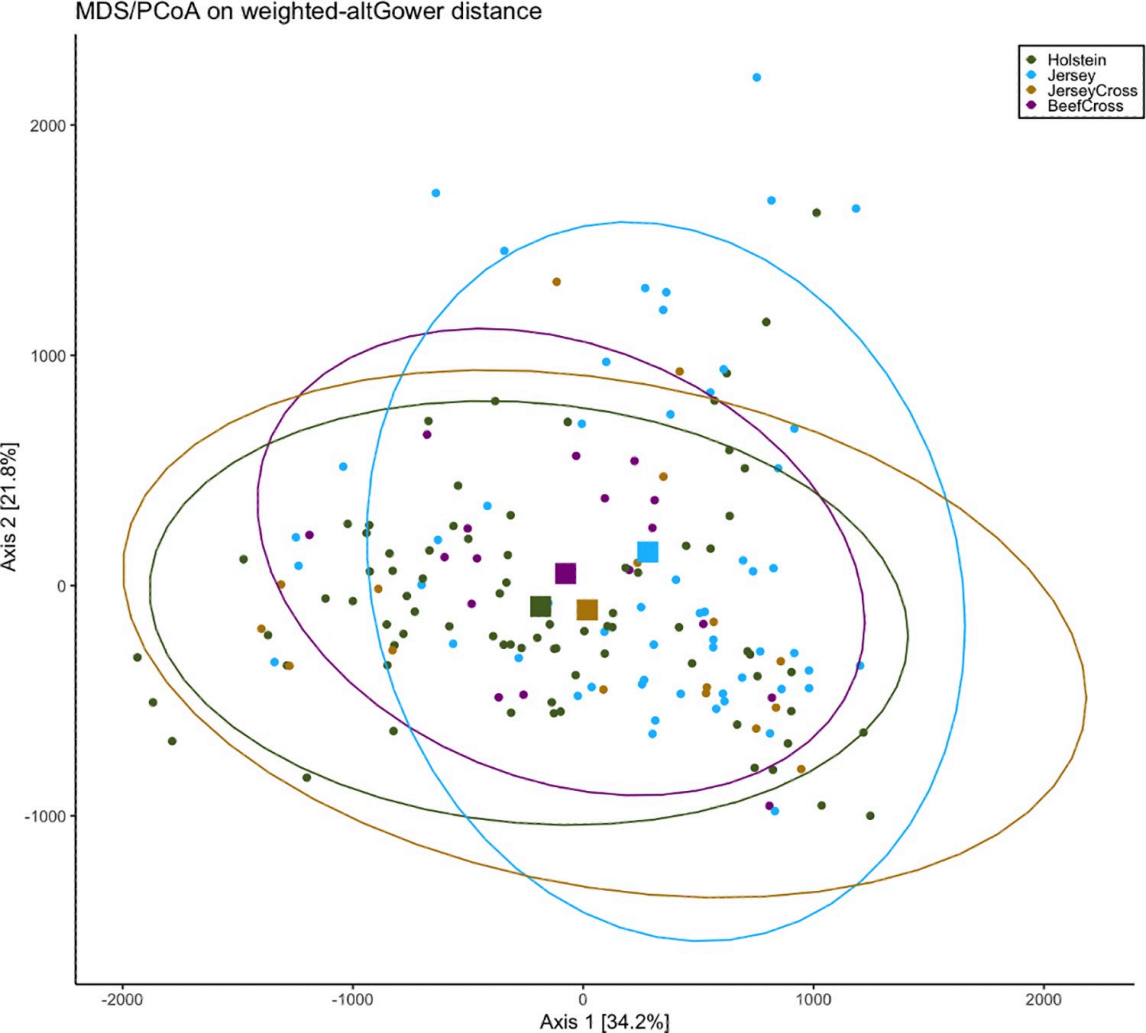
Principal coordinates analysis (PCoA) based on Gower distances grouped by breeds. Proportion of variance explained by each principal coordinate axis is denoted in the corresponding axis label. The squares indicate the centroids for each group. The ellipses represent a 95% confidence interval calculated based on a t-distribution.

According to the Pearson’s chi-squared test based on the number of sequences reads and total reads per sample, the fecal microbiome composition had a different distribution across different health status (healthy versus bright sick versus depressed sick; X^2^ = 1063615, d.f. = 114, p <0.001). A principal coordinate analysis (PCoA) based on Gower distances was performed to explore differences in the microbiota composition of calves with different gradients of GI disease and across different breeds. The results of the beta diversity analysis revealed no clear separation between the different health states (Fig 2) or between the breeds of the calves (Fig 3). Although the centroids representing the three different health states seem to lie within the ellipse encompassing the samples from H calves, indicating higher similarity in composition of the microbiota, the centroid representing the samples of BS calves appear to shift toward the samples from DS calves. Moreover, the centroids representing the different breeds of the calves clustered together, except for Jersey calves on a small scale, suggesting very small or no differences between the microbial communities.

### Composition of fecal microbial community species based on different disease states

**Fig 4:**
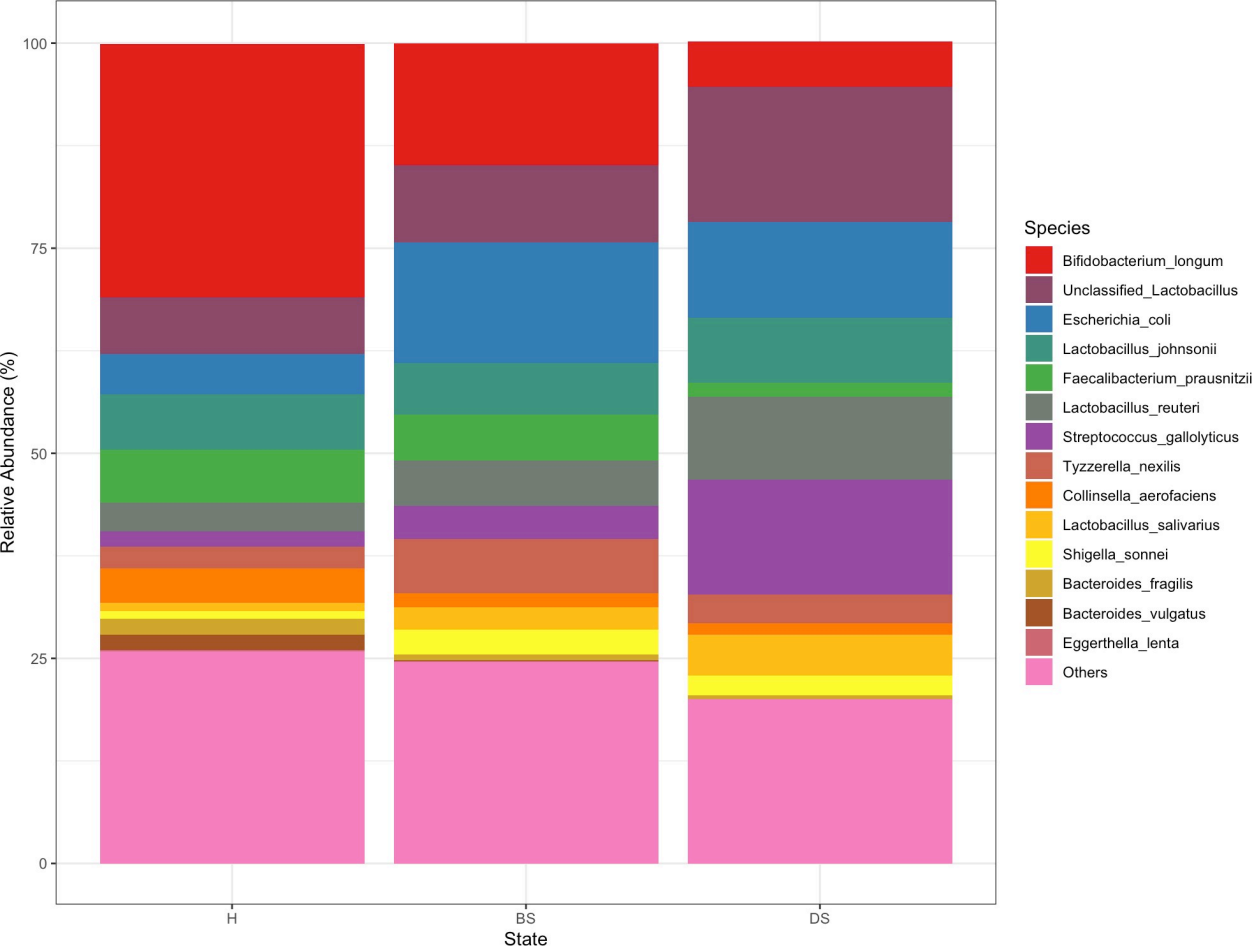
Empirical mean relative abundance at the species level across disease states. In the figure, H = healthy, BS = bright sick, and DS = depressed sick calves.

Four different phyla were identified in the final filtered data. Actinobacteria, Bacteroidetes, Firmicutes, and Proteobacteria accounted for most of the sequences and dominated the fecal microbiome composition regardless of diarrhea occurrence (S1 Appendix). Species abundance was investigated across all samples in calves with and without diarrhea. The percentage of the empirical means of the relative abundance at the species level was calculated for all calves across different disease severity states (Fig 4). Summary statistics of the relative abundance is presented in Table 2; all results of the LME can be found in S2 Table.

**Table 2:** Summarized results of the linear mixed-effects model (LME) for the normalized read counts of relevant species.

Variable description	Estimate	Std. Error	z value	p.value
***Streptococcus gallolyticus***				
**Depressed sick**	2.58	0.88	2.91	0.003
**Jersey**	1.28	0.64	2.01	0.04
**Sampling period**	-1.19	0.50	-2.37	0.01
**Medicated Milk**	2.37	0.79	3.00	0.002
***Bifidobacterium longum***				
**Depressed sick**	-2.4	0.44	-5.46	< 0.001
**Medicated Milk**	0.76	0.34	2.22	0.02
***Escherichia coli***				
**Depressed sick**	1.46	0.34	4.18	< 0.001
**Age at sampling**	-0.16	0.03	-4.90	< 0.001
**Bright sick:Jersey**	1.14	0.42	2.71	0.006
**Unclassified *Lactobacillus***				
**Bright sick**	2.71	0.6	4.51	< 0.001
**beef-cross**	1.18	0.47	2.49	0.01
**Jersey**	1.12	0.48	2.32	0.01
**Sampling period**	-1.45	0.35	-4.14	< 0.001
**Medicated Milk**	1.01	0.38	2.62	0.008
***Lactobacillus reuteri***				
**Bright sick**	1.41	0.41	3.38	< 0.001
**Depressed sick**	1.11	0.44	2.51	0.01
**Sampling period**s	-0.80	0.24	-3.34	< 0.001
**Medicated Milk**	0.70	0.29	2.40	0.01
***Lactobacillus salivarius***				
**Depressed sick**	1.63	0.73	2.23	0.02
***Faecalibacterium prausnitzii***				
**Age at sampling**	-0.18	0.05	-3.16	0.001
**Medicated Milk**	-1.42	0.47	-2.99	0.002

The models accounted for age at sampling, breed, clinical outcomes, source farm on which the calf was born (as a random effect), medicated milk at the time of sampling, enrollment period, and interactions between health outcomes and breed differences. The estimates represent the log of the normalized read counts. The variance explained by the random effect term can be found at the S1 Appendix.

According to the LME, DS (p = 0.003) and Jersey (p = 0.04) calves had a higher relative abundance of *S*. *gallolyticus* relative to H Holstein calves (Table 2). Moreover, we observed a lower relative abundance of *B*. *longum* in DS calves (p<0.001). *E*. *coli* was enriched in DS calves (p<0.001) and in BS Jersey calves relative to H Holstein calves (p = 0.006), and tended to reduce as calf’s age increased (p<0.001). An overall higher relative abundance of unidentified *Lactobacillus* ASV was observed in BS (p<0.001), beef-cross (p = 0.01), and Jersey calves (p = 0.01) relative to H Holstein calves. *L*. *reuteri* was enriched in calves with GI disease, BS (p<0.001) and DS (p = 0.01). In addition, *L*. *salivarius* (p = 0.02) was enriched in calves with severe GI disease (DS).

Fecal samples collected during the second enrollment phase had a lower relative abundance of *S*. *gallolyticus* (p = 0.01), unclassified *Lactobacillus* (p<0.001), and *L*. *reuteri* (p<0.001). In addition, *S*. *gallolyticus* (p = 0.002), *B*. *longum* (p = 0.02), unclassified *Lactobacillus* (p = 0.008), and *L*. *reuteri* (p = 0.01) were enriched on fecal samples of calves at the period they were receiving medicated milk. On the other hand, *F*. *prausnitzii* was reduced in fecal samples of calves during this same period (p = 0.02).

**Fig 5:**
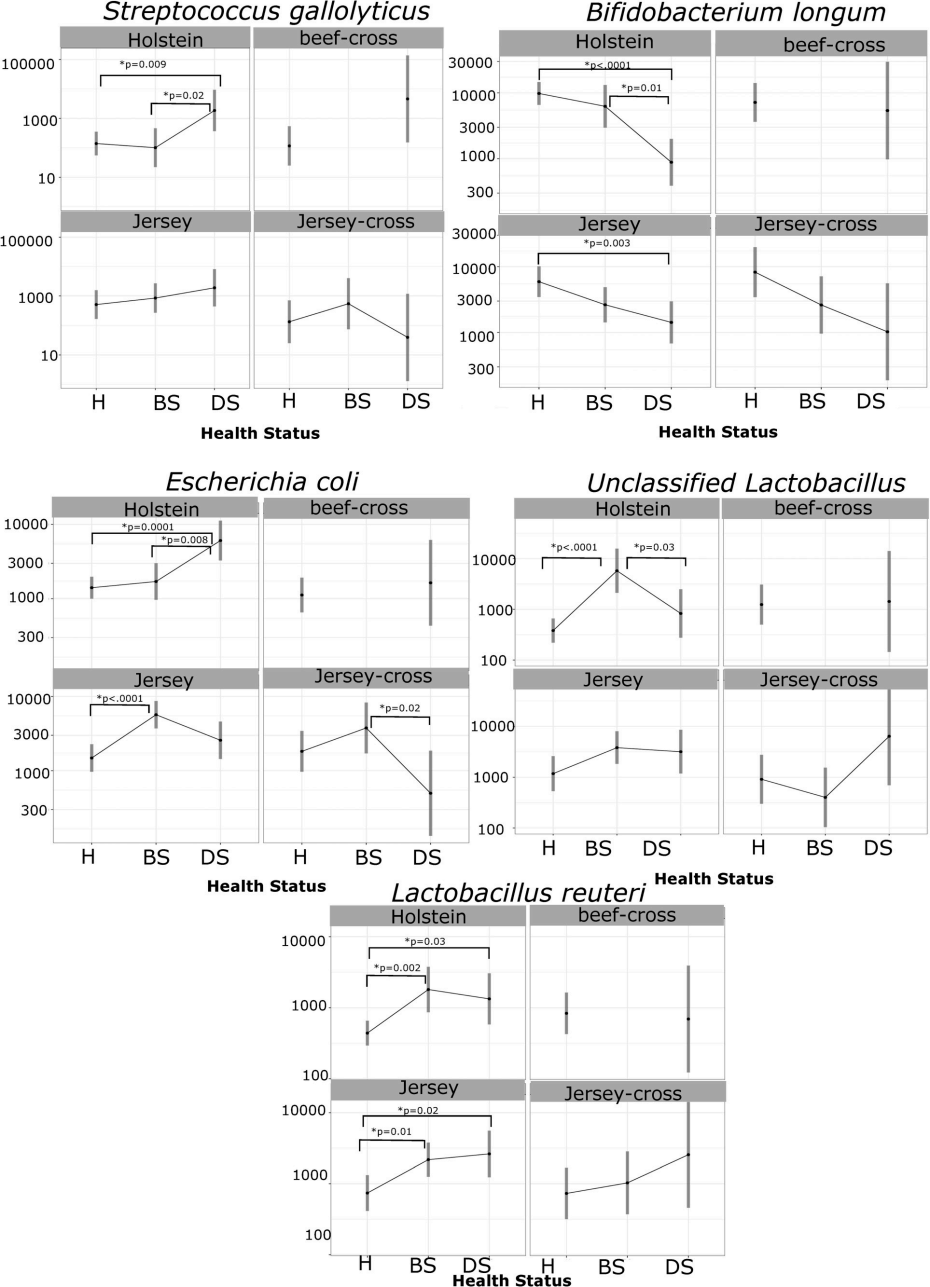
Results of pairwise comparisons within breeds for relevant species. Contrasts were calculated for different breeds across different health outcomes. Estimated marginal means of the normalized read counts on a log10 scale are shown on the y axis and calves’ health status are shown on the x axis (H = healthy, BS = bright sick, and DS = depressed sick calves).

Pairwise comparisons within breeds were calculated for relevant species (Fig 5).

### Disease prediction

A logistic regression model was used in an attempt to predict GI disease based on the fecal microbiome composition of calves. The maximum-likelihood method was used to estimate the negative and positive coefficients associated with diarrhea. A total of 15 coefficients were observed as predictors for diarrhea in calves (S3 Table). A positive coefficient indicates an increase in the odds of having diarrhea, and a negative coefficient is associated with lower odds of having a bright or depressed sick sample.

Coefficients were extracted from a regression model as predictors of disease based on the largest value of lambda such that error is within one standard error of the minimum. The value of lambda was 0.05045. The deviance of the cross-validation model was 0.8875 and the mean squared error was 0.05870. *E*. *lenta*, *B*. *longum*, and *C*. *aerofaciens* showed the lowest coefficient of all predictors (Table 3). *E*. *coli*, followed by species of the genus *Lactobacillus*, had the highest coefficients positively associated with diarrhea prediction.

**Table 3:** Main coefficients of predictors of GI disease in calves.

Predictors	Coefficients
***Eggerthella lenta***	-0.496
***Bifidobacterium longum***	-0.225
***Collinsella aerofaciens***	-0.202
**Breed Jersey**	0.100
***Lactobacillus salivarius***	0.273
***Lactobacillus reuteri***	0.391
***Escherichia coli***	0.598

## Discussion

This study aimed to compare the fecal microbial composition of different breeds of calves with and without GI disease of varying severity in a field setting. Differences in the fecal microbial composition of calves with or without GI disease were most evident rather than between breeds. Healthy (**H**) calves tended to have higher relative abundance of *B*. *longum*, and diseased calves (bright sick [**BS**] and depressed sick [**DS**]) had an enrichment of *E*. *coli* and organisms of the genus *Lactobacillus*. Organisms of the genus *Bifidobacterium* and *Lactobacillus* typically are known for their beneficial probiotic properties [33]. In this study we identified changes in their relative abundance related to disease severity, breed differences, and microbial interactions.

### Disease incidence

During both sampling periods, more than half of the treatments given to the calves were targeting diarrhea. Diarrhea is the most common illness in calves during the first few weeks of life. After approximately three weeks of age the risk of enteric disease decreases and the risk of respiratory disease increases [34]. For that reason, we focused our sampling collection during the period of 4–21 days of age. Overall, Jersey calves were treated for diarrhea more frequently than the other breeds. Interestingly, we did not observe any bright sick beef-cross (Jersey x Angus) calves. Beef operations tend to have different management practices than dairies [35], with greater cow-calf interactions and lower human-calf handling. Therefore, it is known that different factors can influence diarrhea in dairy and beef operations. However, in our study, all calves were raised under the same nutritional and management practices. We were unable to identify any specific fecal microbial composition associated with beef-cross calves that might explain the lower diarrhea incidence, reduced treatment rates, or differences in severity of disease in these calves.

### Beta diversity of the fecal microbiota among calves of different breeds with and without GI disease

The PCoA revealed a large variation in the fecal microbiome of calves within different health status and breeds (Figs 2 and 3). We were not able to distinguish associations of health status or breed specificities with the fecal microbiome community of calves. The fecal microbiome of calves during the first days and weeks of life have been reported to be diverse and change rapidly in early life [15]. Therefore, this large microbial variation observed might have been influenced by the age of the calves. Although the PCoA could not reveal a clear separation between groups, changes in the fecal microbiome composition were mostly associated with differences in the health states of healthy and depressed sick calves and were not breed specific, except for the fecal samples of Jersey calves that slightly clustered away from other samples. Differences in the microbiome profile of calves with and without diarrhea were expected given that enteric disease is a complex disorder often associated with dysbiosis regardless of specific pathogens [36,37]. Changes in the fecal microbiome composition across diarrheic and non-diarrheic calves have been previously reported using different sequencing methods [12,38]. Although we performed clinical assessments twice a day, one of the biggest limitations of our project was recording calves’ fecal score only at the time of sampling. Even though we focused our sampling during the high incidence period of GI disease, it is possible that a calf without any other clinical signs of disease had a fecal score of 3 or 4 only at the time of sampling due to factors unrelated to infectious GI disease, such as stress or feeding, which may have influenced outcomes related to different groups.

Even though all calves were under the same diet and management practices, we were surprised to find few differences in the microbiome profile of different breeds regardless of the presence of diarrhea. For example, we anticipated discovering breed specific characteristics of the microbiome that might help explain the overall higher diarrhea incidence in Jersey calves versus the lower incidence of disease in beef-cross calves. That we did not observe such differences suggests that in our study with the given sample size, it was not possible to identify a breed specific microbiome profile supportive of a breed’s propensity for health or disease. However, the manifestation of clinical signs might be affected by specific interactions between host-microbiome genotypes that were not explored in this study. It is proposed that livestock breeds may host distinct microbial communities due to the interactions between host genotype and microbiota [13]. Previous studies reported differences in the fecal microbiome associated with breed-specificities in boar pigs [39,40]. Similar evaluations targeting the effects of host genetics on the fecal microbiome composition of dairy cattle had not been explored until now [41]. However, analysis focusing on the ruminal microbiome of Holstein and Jersey cows under the same dietary and management conditions showed differences in ruminal bacterial communities associated with genetic factors [13].

### Composition of microbial communities at the species level according to different disease states and breeds

The relative abundance plot showed that healthy calves had an enrichment of *B*. *longum* compared with calves with diarrhea (Fig 4). More precisely, *B*. *longum* accounted for 31% of the relative abundance of all samples from healthy calves (Fig 4). Members of the genus *Bifidobacterium* are abundant in the fecal microbiota of milk-consuming calves [15]. Oligosaccharides present in bovine milk have been reported to enhance the growth of *B*. *longum* [42]. In addition, lower levels of this organism have been linked with several health disorders especially with regard to gut microbiota of infants [43]. Members of this genus play an important role in creating a physical and chemical barrier, increasing immunological and defensive functions, and preventing the growth of pathogens [44]. Moreover, the use of a probiotic containing a *Bifidobacterium* strain has been reported to reduce the frequency of diarrhea occurrence in calves [33].

Although *E*. *coli* was also present in healthy calves, calves with diarrhea had a higher relative abundance of this organism (Fig 4). An overgrowth of this organism is often associate with a disruption of the biome, suggesting dysbiosis in the gut microbiota [45]. As expected, the relative abundance of *E*. *coli* reduced as calves got older and they were less likely to have diarrhea. The lower relative abundance of *E*. *coli* in fecal samples of healthy calves might be explained by the greater presence of *B*. *longum* in this group. *Bifidobacterium* species have been reported to prevent the binding of pathogenic strains of *E*.*coli* [46], and this protective effect against enteropathogens such as *E*. *coli* has been associated with the production of acetate by some *Bifidobacterium* strains [47].

Calves with diarrhea had higher relative abundance of species of the genus *Lactobacillus* (Table 2). This was unexpected given that *Lactobacillus* was reported to be one of the most abundant genera found in fecal samples of healthy and diarrheic calves; however, no differences in the relative abundance of this genus were associated with the occurrence of the disease [12]. The probiotic properties of members of the genus *Lactobacillus* are being studied as potential preventative and therapeutic options for diarrhea in dairy calves [48]. The mechanisms underlying the protective action of this organism can be partly explained by an increase in GI mucus thickness [49], and a reduction in bacterial translocation from the intestine during induced colitis in mice [50]. Given the broad-spectrum probiotic efficacy of these organisms [51], it is plausible that the overgrowth of species of the genus *Lactobacillus* can serve as a restorative mechanism for the gut flora in calves with diarrhea.

Diarrhea is often associated with increased intestinal motility [52], and undigested content, along with microbiota from the small intestine, can be expected to pass more readily. Although greatly varied among individuals, high levels of *Lactobacillus* are present in the small intestine of neonatal calves [53]. In addition, calves with diarrhea often have an overgrowth of *E*. *coli* in the small intestine regardless of the cause of the disease [45]. These findings correlate with the observed enrichment of *Lactobacillus* species and *E*. *coli* in the fecal samples of calves with diarrhea. One other factor that might have contributed to the overgrowth of *Lactobacillus* in calves with diarrhea was the use of milk medicated with neomycin and oxytetracycline. We observed an enrichment of *S*. *gallolyticus*, *B*. *longum*, unclassified *Lactobacillus* ASV, and *L*. *reuteri*, during the period that calves were receiving medicated milk (Table 2). Even though tetracyclines are poorly absorbed in the intestinal tract and their absorption is impaired by milk [54], Oultram et al. [55] observed an increase in *Lactobacillus* in fecal samples of calves treated with different antibiotics such as oxytetracycline. It was suggested that this increase in *Lactobacillus* species in diseased calves out to two weeks post treatment was due to the effect of antibiotics on these bacteria or a delay in the ability of the treated calves to transition from a milk-based diet to a solid feed intake [55]. Even though more studies are needed to determine the impacts of the continuous use of antibiotics on the gut bacteria of dairy calves, the use of this therapy as a prophylactic treatment or for uncomplicated diarrhea cases should be discouraged. In our study, the enrichment of other organisms, such as *B*. *longum*, during the period that calves received medicated milk might be correlated with the young age (five-12d) at which calves received the medicated milk and the inherent fecal microbial composition associated with a milk-based diet consumed during this period.

The linear mixed-effects model showed that depressed sick and Jersey calves had an enrichment of *S*. *gallolyticus* when compared with healthy Holstein calves (Table 2). In addition, pairwise contrasts within breeds showed that depressed sick Holstein calves had an enrichment of *S*. *gallolyticus* when compared with bright sick and healthy Holstein calves (Fig 5). The genus *Streptococcus* has been reported to be almost five times more enriched in diarrheic calves than healthy calves [18]. As with *Lactobacillus*, *Streptococcus* species produce lactic acid that can lower intestinal pH levels [56], inhibiting the growth of organisms such as *E*.*coli* that do not thrive in acidic conditions. Moreover, higher lactate and fecal acidity have been previously associated with the occurrence of diarrhea in dairy calves [57,58], and might be a response associated with changes in the microbial composition during the disease process. The reason for this organism’s enrichment in Jersey calves remains unexplained, but it could be associated with a microbiota structure that is linked with a higher incidence of GI disease.

We also observed a few differences in the microbial community associated with the sampling periods. *S*. *gallolyticus*, unclassified *Lactobacillus*, and *L*. *reuteri* had a lower relative abundance in samples collect from July through August compared with samples collect during the first sampling period, May through June (Table 2). These differences could be a result of changes in management and nutritional practices, or even consequences of environmental factors [59]. One other aspect that has to be taken into consideration is the different sequencing runs for the two datasets. Several errors can be introduced during the workflow [60], and even though all samples were analyzed under the same DNA extraction protocols, sequencing platform, and taxonomic assignment methods, there could be potential differences between sequences runs.

### Disease prediction

Fifteen factors were identified as disease predictors of diarrhea in our dataset (S3 Table). The organisms with the highest coefficient factors associated with lack of disease belonged to the Actinobacteria phylum. *E*. *lenta* was the organism most negatively associated with diarrhea (Table 3). There was a considerable reduction of this organism in diseased calves (Fig 4), although the biological rationale for *E*. *lenta* to be unrelated to diarrhea incidence is unclear. Jersey calves were more likely to have diarrhea than other breeds on the participating calf ranch (Table 3). Furthermore, *E*. *coli* was the greatest predictor of diarrhea although members of the genera *Lactobacillus*, *L*. *salivarius* and *L*. *reuteri* were also strong predictors of diarrhea in this specific farm setting.

The microbiome is a complex and dynamic system. Different factors such as age and diet can influence its composition in different ways. One caveat to the interpretation of our results is that variation within the farm feeding protocol might have impacted our study. Moreover, the use of pasteurized and unpasteurized waste milk has been shown to affect the calf fecal microbiome [61,62]. Although pasteurization has the ability to reduce the microbial load, the concentration of antibiotic residues does not change significantly by pasteurization [63]. Dietary changes such as the type and volume of milk offered to the calves and the preventive use of medicated feed can promote or inhibit specific bacteria. Changes to and variability within feeding programs are likely to occur across dairy farms and calf ranches; therefore, these variations enabled us to analyze outcomes representative of commercial operations. Nonetheless, our primary goal was to describe differences in the fecal microbial composition of calves with varying gradients of GI disease. Although it is challenging to identify the cause of GI disease in neonatal calves and to define an optimal healthy microbiome composition, the associations computed within this study might help advance the development of novel, alternative treatments.

## Conclusions

Our results indicate that there are differences in the fecal microbiome composition of calves with and without GI disease and across different breeds. However, differences were most evident in calves with or without GI disease rather than between breeds. More specifically, species of the genus *Lactobacillus*, *S*. *gallolyticus* and *E*. *coli* were enriched in calves with GI disease. The reason behind the overgrowth of *Lactobacillus* is not entirely clear. However, the higher intestinal passage rate in calves with diarrhea and the use of medicated milk might have impacted the composition of the microbiota, contributing to the higher relative abundance of *Lactobacillus* species in fecal samples of calves with GI disease. However, given the extensive beneficial probiotic properties of *Lactobacillus*, the enrichment of species belonging to this genus might also be associated with an attempt by the host to restore the gut balance during the disease process. Future studies should focus in investigating what precedes the manifestation of clinical signs, such as changes to behavior, appetite, and fecal score, alongside changes to the fecal microbial community of dairy calves with GI disease. These results can be used to help guide producers and veterinarians in their health management decision making.

## Supporting information

S1 Table Total number of samples collected by age, breeds, and different health states. (DOCX)

S2 Table Results of the linear mixed-effects model (LME) for the normalized read counts of relevant species. (DOCX)

S3 Table Coefficients of predictors of GI disease in calves. (DOCX)

S1 File Health and behavior scores of DS calves. (XLSX)

S1 Appendix R Markdown file (pdf) with R code and its output. (PDF)
