# Supplementary material for: Fecal microbiome profiles of neonatal dairy calves with varying severities of gastrointestinal disease
Source: PLoS One. 2022 Jan 4;17(1):e0262317. doi: 10.1371/journal.pone.0262317 (PMC8726473; doi:10.1371/journal.pone.0262317)
Supplement: S1 Table — (DOCX) [file pone.0262317.s001.docx]

**S1 Table. Total number of samples collected by age, breeds, and different health states.**

| **Ages by breed** | **Health states** | | | **Total** |
| --- | --- | --- | --- | --- |
| **Holstein** | **BS** | **DS** | **H** | **Total samples** |
| 4 | 0 | 1 | 0 | 1 |
| 5 | 2 | 1 | 2 | 5 |
| 6 | 1 | 0 | 1 | 2 |
| 7 | 2 | 0 | 5 | 7 |
| 8 | 1 | 2 | 7 | 10 |
| 9 | 3 | 2 | 9 | 14 |
| 10 | 0 | 1 | 6 | 7 |
| 11 | 2 | 0 | 7 | 9 |
| 12 | 0 | 0 | 6 | 6 |
| 13 |  | 1 | 4 | 5 |
| 14 | 1 | 0 | 4 | 5 |
| 15 |  | 1 | 1 | 2 |
| 16 | 0 | 0 | 0 | 0 |
| 17 |  | 0 | 1 | 1 |
| 19 | 0 | 0 | 2 | 2 |
| 20 | 0 | 0 | 1 | 1 |
| 21 | 0 | 0 | 1 | 1 |
| **Total** | **12** | **9** | **57** | **78** |
|  |  |  |  |  |
| **Jersey** |  |  |  |  |
| 4 | 2 | 0 | 1 | 3 |
| 5 | 0 | 3 | 4 | 7 |
| 6 | 3 | 0 | 2 | 5 |
| 7 | 3 | 0 | 1 | 4 |
| 8 | 4 | 5 | 1 | 10 |
| 9 | 4 | 2 | 2 | 8 |
| 10 | 1 | 0 | 2 | 3 |
| 11 | 1 | 1 | 4 | 6 |
| 12 | 1 | 1 | 1 | 3 |
| 13 | 1 | 0 | 2 | 3 |
| 15 | 1 | 0 | 1 | 2 |
| 17 | 0 | 0 | 1 | 1 |
| **Total** | **21** | **12** | **22** | **55** |
|  |  |  |  |  |
| **beef-cross** |  |  |  |  |
| 6 |  |  | 1 | 1 |
| 7 |  | 2 | 2 | 4 |
| 8 |  |  | 4 | 4 |
| 9 |  |  | 1 | 1 |
| 10 |  |  | 1 | 1 |
| 11 |  |  | 1 | 1 |
| 12 |  |  | 1 | 1 |
| 13 |  |  | 2 | 2 |
| 14 |  |  | 1 | 1 |
| 19 |  |  | 1 | 1 |
| **Total** |  | **2** | **15** | **17** |
|  |  |  |  |  |
| **Jersey-cross** |  |  |  |  |
| 5 | 1 | 0 | 1 | 2 |
| 6 | 1 | 0 | 0 | 1 |
| 7 | 2 | 0 | 1 | 3 |
| 8 | 1 | 2 | 0 | 3 |
| 9 | 1 | 0 | 3 | 4 |
| 10 |  |  |  |  |
| 11 |  |  | 1 | 1 |
| 12 |  |  | 2 | 2 |
| 13 |  |  | 1 | 1 |
| **Total** | **6** | **2** | **9** | **17** |
| **Total samples** |  |  |  | **167** |
