# Supplementary material for: Fecal microbiome profiles of neonatal dairy calves with varying severities of gastrointestinal disease
Source: PLoS One. 2022 Jan 4;17(1):e0262317. doi: 10.1371/journal.pone.0262317 (PMC8726473; doi:10.1371/journal.pone.0262317)
Supplement: S2 Table — (DOCX) [file pone.0262317.s002.docx]

**S2 Table. Results of the linear mixed-effects model (LME) for the normalized read counts of relevant species.**

| **Variable description** | Estimate | Std. Error | z value | p.value |  |
| --- | --- | --- | --- | --- | --- |
| Streptococcus gallolyticus |  |  |  |  |  |
| Bright sick | -0.32 | 0.75 | -0.42 | 0.66 |  |
| Depressed sick | 2.58 | 0.88 | 2.91 | 0.003 |  |
| beef-cross | -0.18 | 0.72 | -0.25 | 0.8 |  |
| Jersey | 1.28 | 0.64 | 2.01 | 0.04 |  |
| Jersey-cross | -0.05 | 0.95 | -0.05 | 0.95 |  |
| Age at sampling | 0.12 | 0.12 | 1.05 | 0.29 |  |
| Sampling period | -1.19 | 0.50 | -2.37 | 0.01 |  |
| Medicated Milk | 2.37 | 0.79 | 3 | 0.002 |  |
| Depressed sick:beef-cross | 1.08 | 2.06 | 0.52 | 0.59 |  |
| Bright sick:Jersey | 0.83 | 1.07 | 0.78 | 0.43 |  |
| Depressed sick:Jersey | -1.27 | 1.26 | -1 | 0.31 |  |
| Bright sick:Jersey-cross | 1.73 | 1.48 | 1.16 | 0.24 |  |
| Depressed sick:Jersey-cross | -3.80 | 2.09 | -1.81 | 0.06 |  |
|  |  |  |  |  |  |
| Bifidobacterium longum |  |  |  |  |  |
| Bright sick | -0.44 | 0.41 | -1.00 | 0.27 |  |
| Depressed sick | -2.4 | 0.44 | -5.46 | < 0.001 |  |
| beef-cross | -0.31 | 0.35 | -0.88 | 0.37 |  |
| Jersey | -0.5 | 0.31 | -1.61 | 0.1 |  |
| Jersey-cross | -0.17 | 0.47 | -0.36 | 0.71 |  |
| Age at sampling | 0.08 | 0.04 | 1.86 | 0.06 |  |
| Sampling period | 0.14 | 0.24 | 0.58 | 0.56 |  |
| Medicated Milk | 0.76 | 0.34 | 2.22 | 0.02 |  |
| Depressed sick:beef-cross | 2.12 | 1 | 2.1 | 0.03 |  |
| Bright sick:Jersey | -0.35 | 0.53 | -0.67 | 0.49 |  |
| Depressed sick:Jersey | 0.98 | 0.61 | 1.59 | 0.11 |  |
| Bright sick:Jersey-cross | -0.69 | 0.78 | -0.89 | 0.37 |  |
| Depressed sick:Jersey-cross | 0.32 | 1.03 | 0.03 | 0.75 |  |
|  |  |  |  |  |  |
| Escherichia coli |  |  |  |  |  |
| Bright sick | 0.19 | 0.3 | 0.62 | 0.53 |  |
| Depressed sick | 1.46 | 0.34 | 4.18 | < 0.001 |  |
| beef-cross | -0.22 | 0.28 | -0.81 | 0.41 |  |
| Jersey | 0.05 | 0.25 | 0.2 | 0.83 |  |
| Jersey-cross | 0.25 | 0.34 | 0.74 | 0.45 |  |
| Age at sampling | -0.16 | 0.03 | -4.90 | < 0.001 |  |
| Sampling period | -0.04 | 0.17 | -0.26 | 0.79 |  |
| Medicated Milk | 0.21 | 0.27 | 0.79 | 0.42 |  |
| Depressed sick:beef-cross | -1.08 | 0.78 | -1.37 | 0.17 |  |
| Bright sick:Jersey | 1.14 | 0.42 | 2.71 | 0.006 |  |
| Depressed sick:Jersey | -0.91 | 0.49 | -1.82 | 0.06 |  |
| Bright sick:Jersey-cross | 0.53 | 0.57 | 0.92 | 0.35 |  |
| Depressed sick:Jersey-cross | -2.75 | 0.81 | -3.37 | < 0.001 |  |
|  |  |  |  |  |  |
| Unclassified Lactobacillus |  |  |  |  |  |
| Bright sick | 2.71 | 0.6 | 4.51 | < 0.001 |  |
| Depressed sick | 0.77 | 0.58 | 1.32 | 0.18 |  |
| beef-cross | 1.18 | 0.47 | 2.49 | 0.01 |  |
| Jersey | 1.12 | 0.48 | 2.32 | 0.01 |  |
| Jersey-cross | 0.87 | 0.58 | 1.5 | 0.13 |  |
| Age at sampling | 0.21 | 0.05 | 3.9 | < 0.001 |  |
| Sampling period | -1.45 | 0.35 | -4.14 | < 0.001 |  |
| Medicated Milk | 1.01 | 0.38 | 2.62 | 0.008 |  |
| Depressed sick:beef-cross | -0.63 | 1.36 | -0.46 | 0.64 |  |
| Bright sick:Jersey | -1.53 | 0.77 | -1.98 | 0.04 |  |
| Depressed sick:Jersey | 0.21 | 0.85 | 0.25 | 0.79 |  |
| Bright sick:Jersey-cross | -3.53 | 1 | -3.51 | < 0.001 |  |
| Depressed sick:Jersey-cross | 1.17 | 1.36 | 0.86 | 0.38 |  |
|  |  |  |  |  |  |
| Lactobacillus reuteri |  |  |  |  |  |
| Bright sick | 1.41 | 0.41 | 3.38 | < 0.001 |  |
| Depressed sick | 1.11 | 0.44 | 2.51 | 0.01 |  |
| beef-cross | 0.64 | 0.36 | 1.76 | 0.07 |  |
| Jersey | 0.52 | 0.34 | 1.49 | 0.13 |  |
| Jersey-cross | 0.50 | 0.44 | 1.15 | 0.24 |  |
| Age at sampling | 0.10 | 0.04 | 2.38 | 0.01 |  |
| Sampling period | -0.80 | 0.24 | -3.34 | < 0.001 |  |
| Medicated Milk | 0.70 | 0.29 | 2.40 | 0.01 |  |
| Depressed sick:beef-cross | -1.29 | 1.04 | -1.24 | 0.21 |  |
| Bright sick:Jersey | -0.33 | 0.55 | -0.59 | 0.54 |  |
| Depressed sick:Jersey | 0.15 | 0.64 | 0.23 | 0.81 |  |
| Bright sick:Jersey-cross | -1.07 | 0.75 | -1.42 | 0.15 |  |
| Depressed sick:Jersey-cross | 0.13 | 1.06 | 0.13 | 0.89 |  |
|  |  |  |  |  |  |
| Lactobacillus salivarius |  |  |  |  |  |
| Bright sick | 1.07 | 0.65 | 1.63 | 0.1 |  |
| Depressed sick | 1.63 | 0.73 | 2.23 | 0.02 |  |
| beef-cross | 0.41 | 0.59 | 0.69 | 0.48 |  |
| Jersey | -0.13 | 0.53 | -0.25 | 0.79 |  |
| Jersey-cross | 0.43 | 0.74 | 0.58 | 0.56 |  |
| Age at sampling | 0.04 | 0.06 | 0.71 | 0.47 |  |
| Sampling period | -0.44 | 0.36 | -1.2 | 0.22 |  |
| Medicated Milk | 0.76 | 0.5 | 1.50 | 0.13 |  |
| Depressed sick:beef-cross | -0.34 | 0.000 | 0.00 | 0.99 |  |
| Bright sick:Jersey | 1.04 | 0.89 | 1.17 | 0.24 |  |
| Depressed sick:Jersey | 0.47 | 1.04 | 0.45 | 0.64 |  |
| Bright sick:Jersey-cross | 0.02 | 1.23 | 0.01 | 0.98 |  |
| Depressed sick:Jersey-cross | 0.91 | 1.73 | 0.52 | 0.59 |  |
|  |  |  |  |  |  |
| Faecalibacterium prausnitzii |  |  |  |  |  |
| Bright sick | -0.6 | 0.61 | -0.98 | 0.32 |  |
| Depressed sick | -0.88 | 0.68 | -1.28 | 0.19 |  |
| beef-cross | 0.32 | 0.55 | 0.59 | 0.55 |  |
| Jersey | -0.13 | 0.49 | -0.27 | 0.78 |  |
| Jersey-cross | -0.36 | 0.69 | -0.52 | 0.60 |  |
| Age at sampling | -0.18 | 0.05 | -3.16 | 0.001 |  |
| Sampling period | 0.41 | 0.34 | 1.19 | 0.23 |  |
| Medicated Milk | -1.42 | 0.47 | -2.99 | 0.002 |  |
| Depressed sick:beef-cross | -27.57 | 19404.89 | -0.00 1 | 0.99 |  |
| Bright sick:Jersey | -0.92 | 0.83 | -1.11 | 0.26 |  |
| Depressed sick:Jersey | -1.41 | 0.97 | -1.44 | 0.14 |  |
| Bright sick:Jersey-cross | 1.23 | 1.15 | 1.06 | 0.28 |  |
| Depressed sick:Jersey-cross | -26.87 | 17884.96 | -0.002 | 0.99 |  |
|  |  |  |  |  |  |
| Collinsella aerofaciens |  |  |  |  |  |
| Bright sick | -1.32 | 0.55 | -2.39 | 0.01 |  |
| Depressed sick | -0.37 | 0.61 | -0.61 | 0.54 |  |
| beef-cross | -0.28 | 0.49 | -0.56 | 0.57 |  |
| Jersey | 0.29 | 0.44 | 0.67 | 0.50 |  |
| Jersey-cross | -0.47 | 0.63 | -0.74 | 0.45 |  |
| Age at sampling | 0.16 | 0.05 | 3.04 | 0.002 |  |
| Sampling period | -0.25 | 0.3 | -0.83 | 0.40 |  |
| Medicated Milk | 0.91 | 0.42 | 2.14 | 0.03 |  |
| Depressed sick:beef-cross | -0.37 | 0.000 | 0.000 | 0.99 |  |
| Bright sick:Jersey | 0.71 | 0.75 | 0.94 | 0.34 |  |
| Depressed sick:Jersey | -1.35 | 0.88 | -1.53 | 0.12 |  |
| Bright sick:Jersey-cross | 1.61 | 1.04 | 1.54 | 0.12 |  |
| Depressed sick:Jersey-cross | -1.21 | 1.46 | -0.83 | 0.4 |  |
|  |  |  |  |  |  |
| Shigella sonnei |  |  |  |  |  |
| Bright sick | 0.16 | 0.32 | 0.49 | 0.61 |  |
| Depressed sick | 1.39 | 0.37 | 3.75 | < 0.001 |  |
| beef-cross | -0.27 | 0.29 | -0.91 | 0.35 |  |
| Jersey | -0.02 | 0.27 | -0.09 | 0.92 |  |
| Jersey-cross | 0.37 | 0.36 | 1 | 0.31 |  |
| Age at sampling | -0.15 | 0.03 | -4.36 | < 0.001 |  |
| Sampling period | -0.08 | 0.18 | -0.42 | 0.67 |  |
| Medicated Milk | 0.21 | 0.29 | 0.73 | 0.46 |  |
| Depressed sick:beef-cross | -1.06 | 0.83 | -1.26 | 0.2 |  |
| Bright sick:Jersey | 1.32 | 0.45 | 2.91 | 0.003 |  |
| Depressed sick:Jersey | -0.64 | 0.53 | -1.22 | 0.22 |  |
| Bright sick:Jersey-cross | 0.39 | 0.61 | 0.64 | 0.52 |  |
| Depressed sick:Jersey-cross | -2.66 | 0.87 | -3.06 | 0.002 |  |
|  |  |  |  |  |  |
| Bacteroides fragilis |  |  |  |  |  |
| Bright sick | -1.17 | 0.9 | -1.23 | 0.21 |  |
| Depressed sick | -2.08 | 1.01 | -2.05 | 0.03 |  |
| beef-cross | -0.54 | 0.81 | -0.67 | 0.50 |  |
| Jersey | 0.72 | 0.73 | 0.99 | 0.32 |  |
| Jersey-cross | -0.28 | 1.03 | -0.28 | 0.77 |  |
| Age at sampling | -0.2 | 0.08 | -2.38 | 0.01 |  |
| Sampling period | -0.46 | 0.5 | -0.91 | 0.35 |  |
| Medicated Milk | -0.48 | 0.7 | -0.69 | 0.48 |  |
| Depressed sick:beef-cross | -0.31 | 0.000 | 0.000 | 1 |  |
| Bright sick:Jersey | -0.6 | 1.22 | -0.49 | 0.62 |  |
| Depressed sick:Jersey | 0.26 | 1.44 | 0.18 | 0.85 |  |
| Bright sick:Jersey-cross | 0.44 | 1.7 | 0.25 | 0.79 |  |
| Depressed sick:Jersey-cross | -0.58 | 2.39 | -0.24 | 0.8 |  |
