# Supplementary material for: Fecal microbiome profiles of neonatal dairy calves with varying severities of gastrointestinal disease
Source: PLoS One. 2022 Jan 4;17(1):e0262317. doi: 10.1371/journal.pone.0262317 (PMC8726473; doi:10.1371/journal.pone.0262317)
Supplement: S3 Table — (DOCX) [file pone.0262317.s003.docx]

**S3 Table. Coefficients of predictors of GI disease in calves.**

| Predictors | Coefficients |
| --- | --- |
| Eggerthella lenta | -0.496 |
| Bifidobacterium longum | -0.225 |
| Collinsella aerofaciens | -0.202 |
| Bacteroides vulgatus | -0.075 |
| Bacteroides fragilis | -0.064 |
| Lachnoclostridium pacaense | -0.063 |
| Age at sampling | -0.043 |
| Pseudoflavonifractor capillosus | -0.017 |
| Faecalicoccus pleomorphus | -0.005 |
| Tyzzerella nexilis | 0.065 |
| Streptococcus gallolyticus | 0.087 |
| Breed Jersey | 0.100 |
| Lactobacillus salivarius | 0.273 |
| Lactobacillus reuteri | 0.391 |
| Escherichia coli | 0.598 |
