# Supplementary material for: Fecal microbiome profiles of neonatal dairy calves with varying severities of gastrointestinal disease
Source: PLoS One. 2022 Jan 4;17(1):e0262317. doi: 10.1371/journal.pone.0262317 (PMC8726473; doi:10.1371/journal.pone.0262317)

Dada2 workflow

Giovana Slanzon and Ben Ridenhour

8/12/2021

DADA2 pipeline

```
library("dada2")

library("ggplot2")

fqs <-dir() #list all of the files in the working directory, and save as fqs
fqs <- fqs[grep(".fastq.gz$",fqs)] #remove any file from fqs that isn't a "*.
fastq.gz" file

fqs.for <- fqs[grep("_R1",fqs)] #forward group of fastq files, grep looks for
the pattern "_R1"
fqs.rev <- fqs[grep("_R2",fqs)] #reverse group of fastq files, grep looks for
the pattern "_R2"

sample.names <- sapply(strsplit(fqs.for, "_"), `[`, 2)
#Let's output some plots from sample 1's reads
plotQualityProfile(fqs.for[[1]]) + ggtitle("Quality Scores for Forward Reads"
)
```

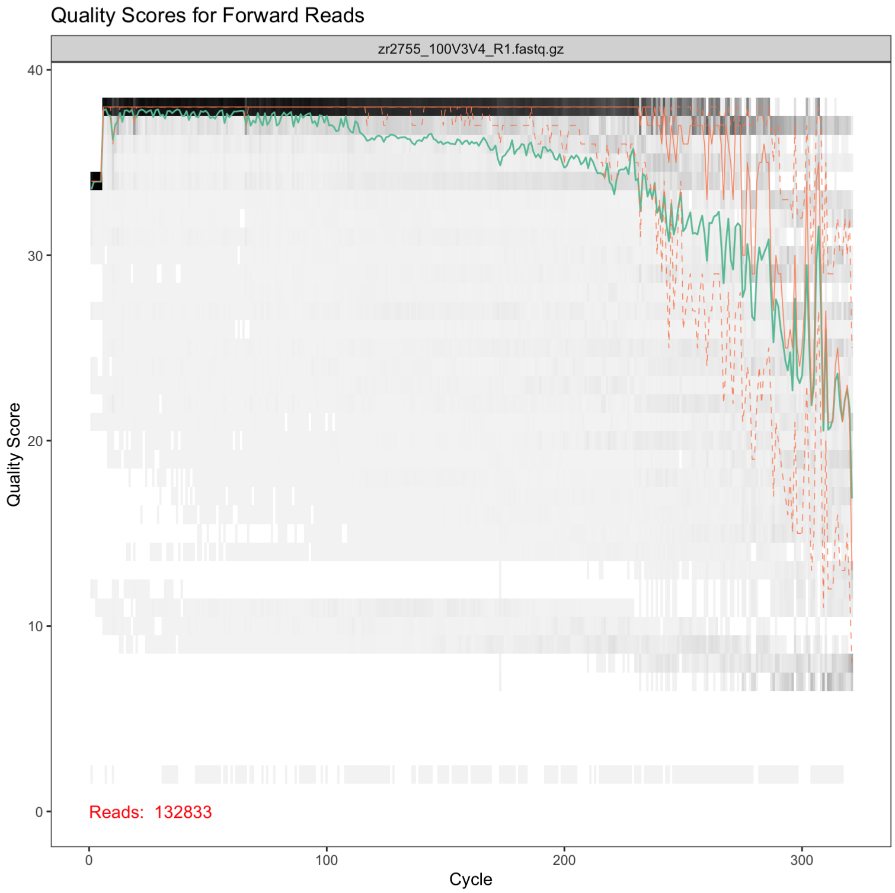


```r
plotQualityProfile(fqs.rev[[1]]) + ggtitle("Quality Scores for Reverse Reads"
)
```

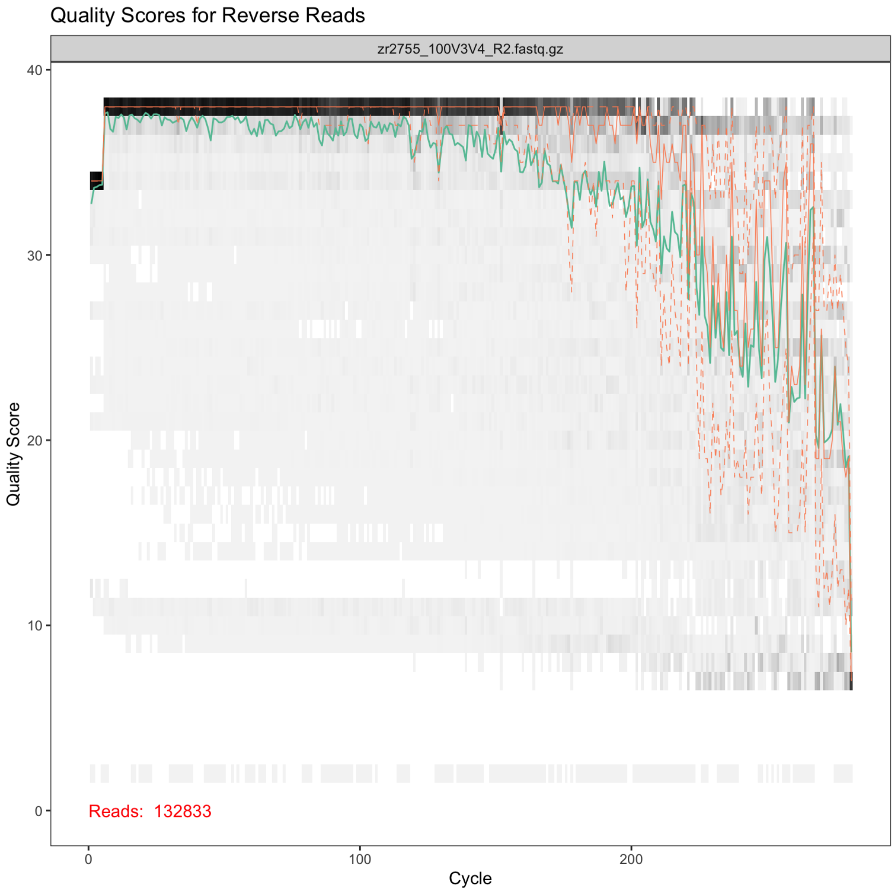


```
plotQualityProfile(fqs.for[[2]]) + ggtitle("Quality Scores for Forward Reads"
)
```

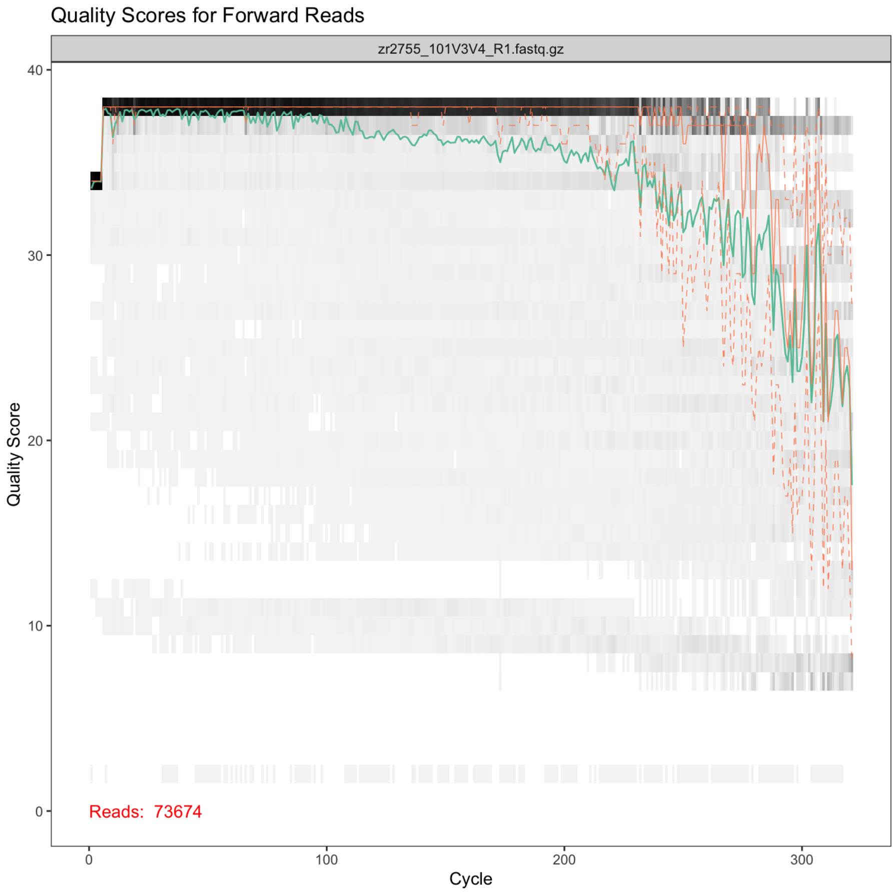


```
plotQualityProfile(fqs.rev[[2]]) + ggtitle("Quality Scores for Reverse Reads"
)
```

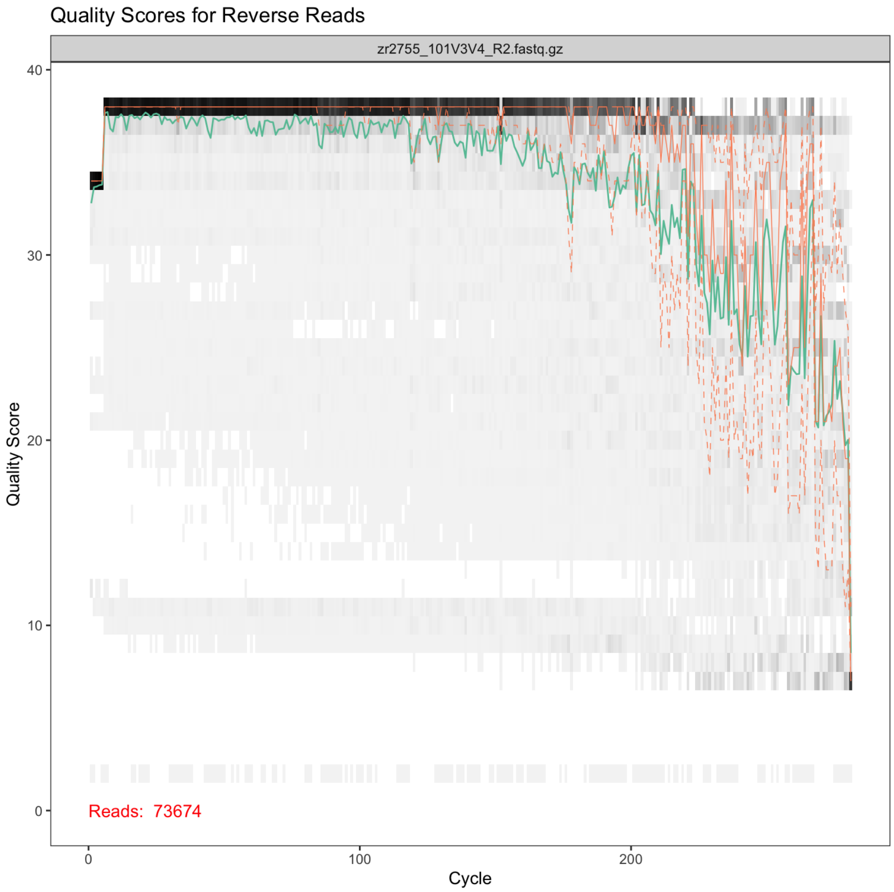


```
plotQualityProfile(fqs.for[[3]]) + ggtitle("Quality Scores for Forward Reads"
)
```

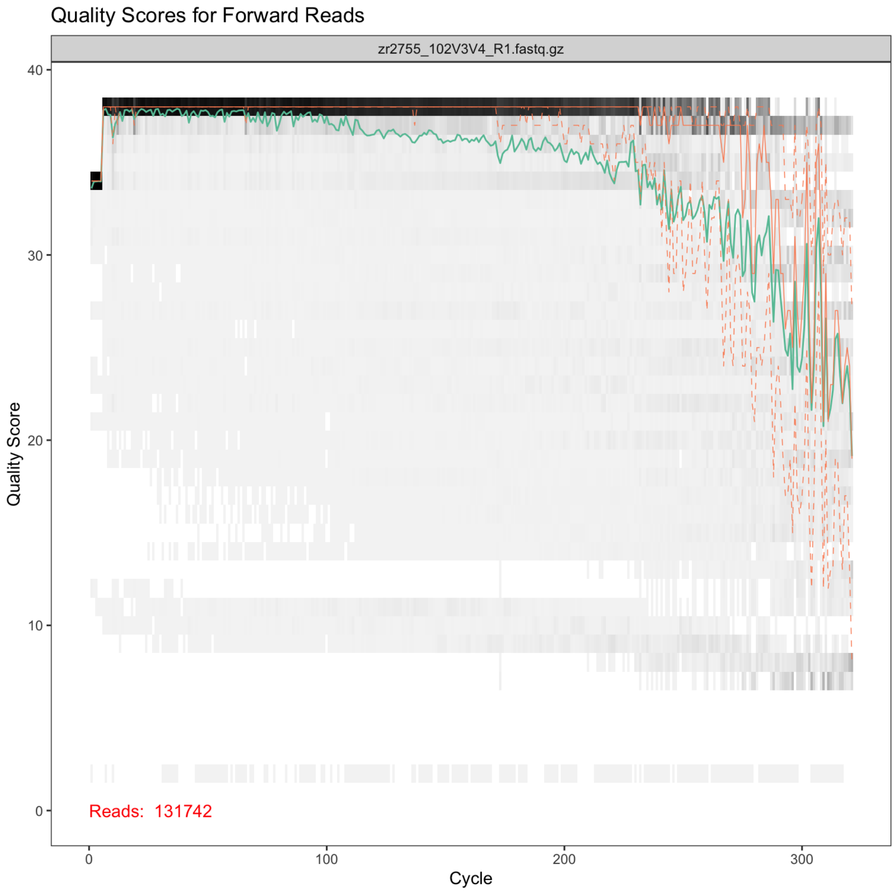


```
plotQualityProfile(fqs.rev[[3]]) + ggtitle("Quality Scores for Reverse Reads"
)
```

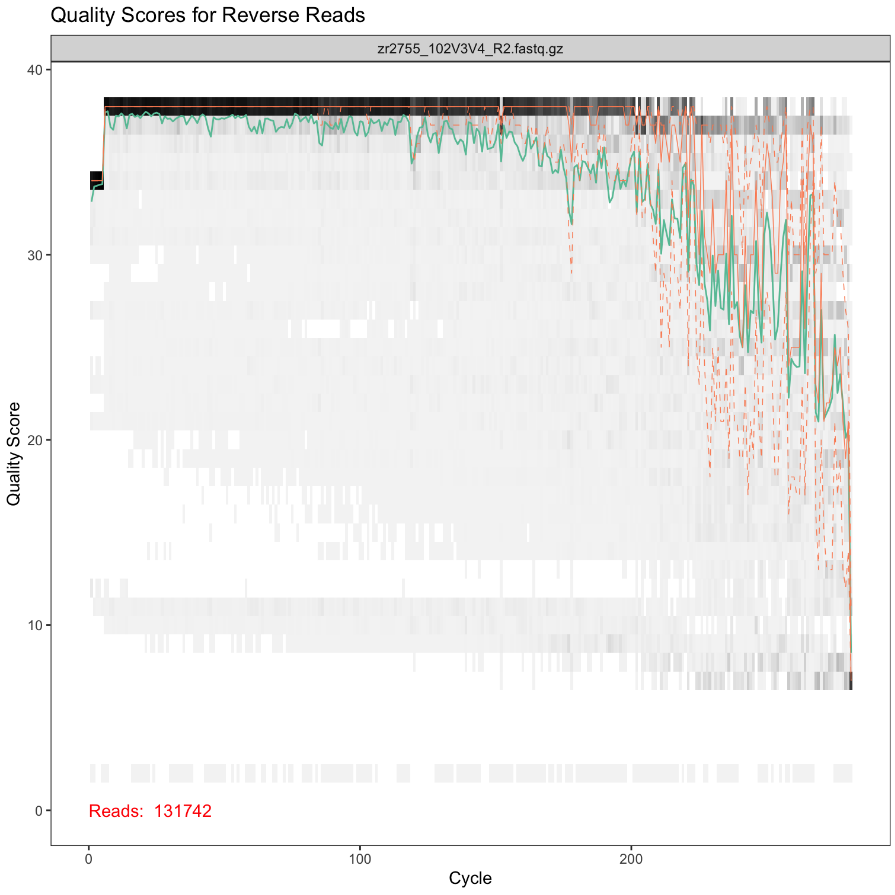

Make directory and filenames for the filtered fastqs

```
filt_path <- file.path(".", "filtered")
if(!file_test("-d", filt_path)) dir.create(filt_path)
filtFs <- file.path(filt_path, paste0(sample.names, "_F_filt.fastq.gz"))
filtRs <- file.path(filt_path, paste0(sample.names, "_R_filt.fastq.gz"))
# Filter and trim
## trimLeft = c(10,10) says drop the first 10 base pairs from both the F and
R reverse reads
## truncLen = c(300,225) says to trim the F at 300bp in length and the R at 2
25bp in length
## maxN = 0, no "N" nucleotides allowed in the sequence
## max EE = maximum expected errors over the entire read, set to 2
## truncQ = 2, drop an reads where a nucleotide a Q score of less than 2 (ie.
20)
out <- filterAndTrim(fqs.for, filtFs, fqs.rev,filtRs,
                     trimLeft=c(10, 10), truncLen=c(300,225),
                     maxN=0, maxEE=2, truncQ=2, matchIDs = T,
                     compress=TRUE, verbose=TRUE, multithread = 20)
#dereplicate the data
derepFs <- derepFastq(filtFs, verbose=TRUE)
derepRs <- derepFastq(filtRs, verbose=TRUE)
```

```
# Name the derep-class objects by the sample names
names(derepFs) <- sample.names
names(derepRs) <- sample.names

errF <- learnErrors(derepFs, multithread = 20)
errR <- learnErrors(derepRs, multithread = 20)

dadaFs <- dada(derepFs, err=errF, selfConsist = TRUE, multithread = 20)
dadaRs <- dada(derepRs, err=errR, selfConsist = TRUE, multithread = 20)
###
mergers <- mergePairs(dadaFs, derepFs, dadaRs, derepRs, verbose=TRUE)
save(file = "MergedReads2.RData", mergers)
load("MergedReads.RData")

seqtab <- makeSequenceTable(mergers)
dim(seqtab)
seqtab.nochim <- removeBimeraDenovo(seqtab, verbose=TRUE, multithread = 12)
dim(seqtab.nochim)
save(file = "SeqTab2.RData", seqtab)
getN <- function(x) sum(getUniques(x))
track <- cbind(out, sapply(dadaFs, getN), sapply(dadaRs, getN), sapply(merger
s, getN), rowSums(seqtab.nochim))

colnames(track) <- c("input", "filtered", "denoisedF", "denoisedR", "merged",
"nonchim")
rownames(track) <- sample.names
head(track)

sum(track[,6])/sum(track[,1]) #fraction of reads kept at the end
hist(track[,6]/track[,1],20, xlab="Fraction Retained", main = NULL) #histogra
m of fraction of reads kept in each sample
dim(seqtab.nochim) #106 samples x 2586 SVs

hist(colSums(seqtab.nochim),100, xlab="Read Counts per SV", main=NULL) #histo
gram of read counts per SV
###make a fasta file for SPINGO
sink("FMTPart1.fasta", append = T)
for(i in seq_len(ncol(seqtab.nochim))){
  cat(paste(">ASV",i, "\n", sep=""))
  cat(paste(colnames(seqtab.nochim))[i], "\n")
}
sink()
```

Read in sequence table and merge with metadata

```
load("SeqTab.RData")
library(dada2)
seqtab.nochim <- removeBimeraDenovo(seqtab, verbose=TRUE, multithread = 12)
```

```
counttab <- seqtab.nochim
colnames(counttab) <- paste("ASV",1:ncol(seqtab.nochim),sep="") #rename colum
n of seqtab to ASV1, ASV2, ... , ASV2586

counttab <- t(counttab)
counttab <- as.data.frame(counttab)
counttab$Species <- ids[match(rownames(counttab),ids$Species),"Species.1"]

counttab.agg <- aggregate(counttab[,-107], by=counttab[,107,drop=F], sum)
counttab.agg <- counttab.agg[order(rowSums(counttab.agg[,-1]), decreasing = T
),]

ids <- read.xlsx("../ASV_Final_Table_BLAST.xlsx",1)

#get rid of V3, V4 which are the L1 (group) assignments from SPINGO
ids[,c(3:4)] <- NULL
 names(ids) <- c("ASV", "Kmer_Similarity", "Genus", "Genus_Bootstrap", "Speci
es", "Species_Bootstrap")

counttab <- seqtab.nochim
colnames(counttab) <- paste("ASV",1:ncol(seqtab.nochim),sep="") #rename colum
n of seqtab to ASV1, ASV2, ... , ASV3358

###Get the sequence of an ambiguous ASV and BLAST them:
getSequenceByASV <- function(asvName){
  colnames(seqtab.nochim)[which(colnames(counttab) == asvName)]
}

getSequenceByASV("ASV4")

sapply(c("ASV4","ASV10","ASV12"), getSequenceByASV)

counttab <- t(counttab)
counttab <- as.data.frame(counttab)
counttab$Species <- ids[match(rownames(counttab),ids$ASV),"Species"]

counttab.agg <- aggregate(counttab[,-1*ncol(counttab)], by=counttab[,ncol(cou
nttab),drop=F], sum)
counttab.agg <- counttab.agg[order(rowSums(counttab.agg[,-1]), decreasing = T
),]

metadata <- read.xlsx("../MasterSheet_All_Samples_02-25_20.xlsx")
metadata$Zymo_foo <- ifelse(metadata$Part == "_p2",2929,2755)
metadata$Zymo_N <- ifelse(metadata$Part == "_p2", metadata$LabID, as.numeric(
substr(metadata$Sample_n., start = 12, stop = 100000)))
metadata$Zymo_foo <- paste("zr",metadata$Zymo_foo,"_",metadata$Zymo_N,"V3V4",
sep="")

metadata$Breed <- as.factor(metadata$Breed)
```

```
levels(metadata$Breed) <- c("Holstein", "Holstein", "Jersey", "Jersey", "Beef
Cross", "JerseyCross", "JerseyCross")

analysisData <- as.data.frame(t(counttab.agg[,-1]))
colnames(analysisData) <- counttab.agg[,1]
analysisData$ZymoSample <- colnames(counttab.agg[,-1])


subData$Zymo_foo <- analysisData$ZymoSample
subData <- merge(subData,metadata[,c("Zymo_foo", "SampleType","Breed","Sample
Age")]) #add in sample type to asv data
```

Filtering

##Filtering methods

Load packages, data and functions

```
packages = c("openxlsx","Tmisc","dirmult","imputeTS","kableExtra","compositio
ns","stringr","dplyr","pracma","splus2R","matrixcalc","plotly","reshape","vir
idis","Hmisc","gridExtra","DT","infotheo","networkD3","igraph","WGCNA","micEc
on","decontam","inflection","gplots","boot","kableExtra","qwraps2","caret","g
grepel","network","ggnet", "GGally")

invisible(lapply(packages, library,character.only=TRUE))

##data
dta=read.xlsx("~/Desktop/Analysis_Part1/analysisData.xlsx", rowNames = TRUE)
rownames(dta) = dta$ZymoSample
dta$...1=NULL
Samples =dta$ZymoSample
dta$ZymoSample=NULL

## functions
source("~/Desktop/Analysis_Part1/Info.R")
source("~/Desktop/Analysis_Part1/perm.info.R")
source("~/Desktop/Analysis_Part1/BootStrap.info.R")


## find those ASVs with 0 across all samples
names(which(colSums(dta)==0))

##  [1] "Desulfonatronobacter_acidivorans"
##  [2] "Anaerostipes_hadrus"
##  [3] "Eubacterium_ruminantium"
##  [4] "Bifidobacterium_thermacidophilum"
##  [5] "Parabacteroides_Uncultured_bacterium"
##  [6] "Bifidobacterium_animalis"
##  [7] "Clostridium_bornimense"
```

```
##  [8] "Ruminococcus_champanellensis"
##  [9] "Eubacterium_pyruvativorans"
## [10] "Natranaerovirga_pectinivora"
## [11] "Rhodospirillum_rubrum"
## [12] "Eubacterium_ramulus"
## [13] "Roseburia_intestinalis"
## [14] "Desulfotomaculum_nigrificans"
## [15] "Pelobacter_propionicus"
## [16] "Olsenella_uli"
## [17] "Kandleria_vitulina"
## [18] "Anaerostipes_Uncultured_bacterium"
## [19] "Peptococcus_simiae"
## [20] "Lutispora_thermophila"

dta= dta[,-as.vector(which(colSums(dta)==0))]
```

Load other functions

```
## required functions

# number of bins
nbins.scott=function (x)
{
  h <- stats::sd(x)
  if (h == 0)
    h <- stats::mad(x, constant = 2)
  if (h > 0)
    ceiling(diff(range(x))/(3.49 * h * length(x)^(-1/3)))
  else 1L
}
```

Fit the power law distribution to empirical data in log space

```
scaleFreeR2= function (connectivity,plt=TRUE,histo=TRUE){
  ## Use FD method for binning
  nbins = nbins.scott(connectivity)
  if(nbins == 1)
    nbins<-nbins+1
  k = connectivity
  discretized.k = cut(k, nbins)
  dk = tapply(k, discretized.k, mean)  ## mean of values in each interval
  p.dk = as.vector(tapply(k, discretized.k, length)/length(k)) ## relative fr
eq
  breaks1 = seq(from = min(k), to = max(k), length = nbins +1)
  if(histo==TRUE){
    hist1 =hist(k, breaks = breaks1)
  }
  else{
    hist1 =hist(k, breaks = breaks1,plot = FALSE)
  }
```

```
  dk2 = hist1$mids
  dk = ifelse(is.na(dk), dk2, dk)
  dk = ifelse(dk == 0, dk2, dk)
  p.dk = ifelse(is.na(p.dk), 0, p.dk)
  log.dk = as.vector(log10(dk))
  log.p.dk = as.numeric(log10(p.dk + 1e-09))
  lm1 = lm(log.p.dk ~ log.dk)
  OUTPUT = data.frame(scaleFreeRsquared = round(summary(lm1)$r.squared,
                                                 2), slope = round(lm1$coeffic
ients[[2]], 2))
  title = paste(" scale free R^2=", as.character(round(summary(lm1)$r.squared
,
                                                       2)), ", slope=", round
(lm1$coefficients[[2]], 2))
  if(plt==TRUE){
    suppressWarnings(plot(log.dk, log.p.dk, xlab = "log10(k)",
                          ylab = "log10(p(k))", main = title))
    lines(log.dk, predict(lm1), col = 1)
  }

  OUTPUT
}

boot.R2= function(deg,b=NULL,plt=TRUE,histo=TRUE){
  nbins = nbins.scott(deg)
  if(nbins == 1)
    nbins<-nbins+1
  k = deg
  discretized.k = cut(k, nbins)
  dk = tapply(k, discretized.k, mean)  ## mean of values in each interval
  p.dk = as.vector(tapply(k, discretized.k, length)/length(k)) ## relative fr
eq
  breaks1 = seq(from = min(k), to = max(k), length = nbins +1)
  if(histo==TRUE){
    hist1 =hist(k, breaks = breaks1)
  }
  else{
    hist1 =hist(k, breaks = breaks1,plot = FALSE)
  }

  dk2 = hist1$mids
  dk = ifelse(is.na(dk), dk2, dk)
  dk = ifelse(dk == 0, dk2, dk)
  p.dk = ifelse(is.na(p.dk), 0, p.dk)
  log.dk = as.vector(log10(dk))
  log.p.dk = as.numeric(log10(p.dk + 1e-09))
  dta = data.frame(log.p.dk,log.dk)
  rsq <- function(formula, data, indices){
    d<- data[indices,]
    fit<- lm(formula,data = d)
```

```
    return(summary(fit)$r.square)
  }
  results <- suppressWarnings(boot(data=dta, statistic=rsq,
                                   R=b, formula=log.p.dk~log.dk))
  rsqrd = mean(as.vector(results$t),na.rm=TRUE)
  return(rsqrd)
}
```

Mutual Information

```
## We estimate mutual iformation and marginal entropies
##and normalized mutual information by joint entropy
mi.res = mutinformation(dta,method = "emp")   ## mutual information between e
ach pair of taxa
marg.res = apply(dta,2,entropy,method = "emp")  ## marginal entropy of each t
axon

# calculate joint and conditional entropy
cond.ent= NULL
joint.ent= NULL
for ( i in 1: dim(dta)[2]){
  cond.ent.vec=c()
  joint.ent.vec=c()
  for(j in 1: dim(dta)[2]){
    cond.ent.vec = c(cond.ent.vec,round(condentropy(dta[,i],dta[,j],method="e
mp"),3))
    joint.ent.vec = c(joint.ent.vec,round(cond.ent.vec[j]+entropy(dta[,j],met
hod = "emp"),3))
  }
  cond.ent = rbind(cond.ent,cond.ent.vec)
  joint.ent = rbind(joint.ent,joint.ent.vec)
}

colnames(cond.ent) <-colnames(dta)
colnames(joint.ent) <-colnames(dta)

## weighted network adjacency matrix
I_adj_univ = round(mi.res/joint.ent,3) ## 2 decimal places makes the matrix t
o be symmetric
#isSymmetric(I_adj_univ)

#  matrix symmetric
I_adj_univ[lower.tri(I_adj_univ)] = t(I_adj_univ)[lower.tri(I_adj_univ)]
#isSymmetric(I_adj_univ)
```

Calculate mean degree and R2 (goodness of fit)

```
tau =seq(0.05,0.95,0.05)

# threshold based on median degree
R2=c()
```

```r
mean.deg=c()
slope=c()
Rsqrd.boot=c()

for( i in 1: length(tau)){
  I_unw = ifelse(I_adj_univ<tau[i],0,1)
  diag(I_unw)=0
  bb = mean(apply(I_unw,1,sum))
  r2 = scaleFreeR2(apply(I_unw,1,sum),plt=FALSE,histo = FALSE)$scaleFreeRsqua
red
  gamma = scaleFreeR2(apply(I_unw,1,sum),plt=FALSE,histo=FALSE)$slope
  R2 = c(R2,r2)

  r2.boot = boot.R2(apply(I_unw,1,sum),b=5000,plt=FALSE,histo = FALSE)
  Rsqrd.boot = c(Rsqrd.boot,r2.boot)
  slope = c(slope,gamma)
  mean.deg=c(mean.deg,bb)
  #print(i)
}

df <- data.frame(thresh=tau,tau=tau,Rsqrd=Rsqrd.boot,meandeg = mean.deg,Slope
=slope)

p1 = ggplot(data=df,aes(x=tau,y=(Rsqrd))) +
  geom_point(size=4,col="red") +
  # geom_line(size=1,col="red") +
  geom_text(aes(label="",hjust=0,vjust=2))+
  theme_bw()+xlab("Threshold")+ylab("R-squared")+
  theme(axis.text.x=element_text(angle=45, hjust=1,size=12)) +
  theme(axis.text.y=element_text(hjust=1,size=12),
        axis.title=element_text(size=12,face="bold"),
        legend.text=element_text(size=10),
        legend.title=element_text(size=12,face="bold")) +
  theme(plot.title = element_text(size=14))+
  xlab("Thresholds")+
  scale_x_continuous(breaks = seq(0.05,0.95,0.05))+
  #geom_text(aes(0.5, .92, label="R2:0.97", color=""))+
  theme(legend.position = "none") +
  scale_y_continuous(limits = c(0,1))+geom_line()

p2=ggplot(data=df,aes(x=tau,y=(meandeg))) +
  geom_point(size=4,col="red") +
  #geom_line(size=1,col="red") +
  geom_text(aes(label="",hjust=0,vjust=2))+
  theme_bw()+xlab("Threshold")+ylab("mean degree")+
  theme(axis.text.x=element_text(angle=45, hjust=1,size=12)) +
  theme(axis.text.y=element_text(hjust=1,size=12),
        axis.title=element_text(size=12,face="bold"),
        legend.text=element_text(size=10),
```

```
        legend.title=element_text(size=12,face="bold")) +
  theme(plot.title = element_text(size=14))+
  xlab("Thresholds")+
  scale_x_continuous(breaks = seq(0.05,0.95,0.05))+
  theme(legend.position = "none")+geom_line()

grid.arrange(p1,p2,ncol=2)
```

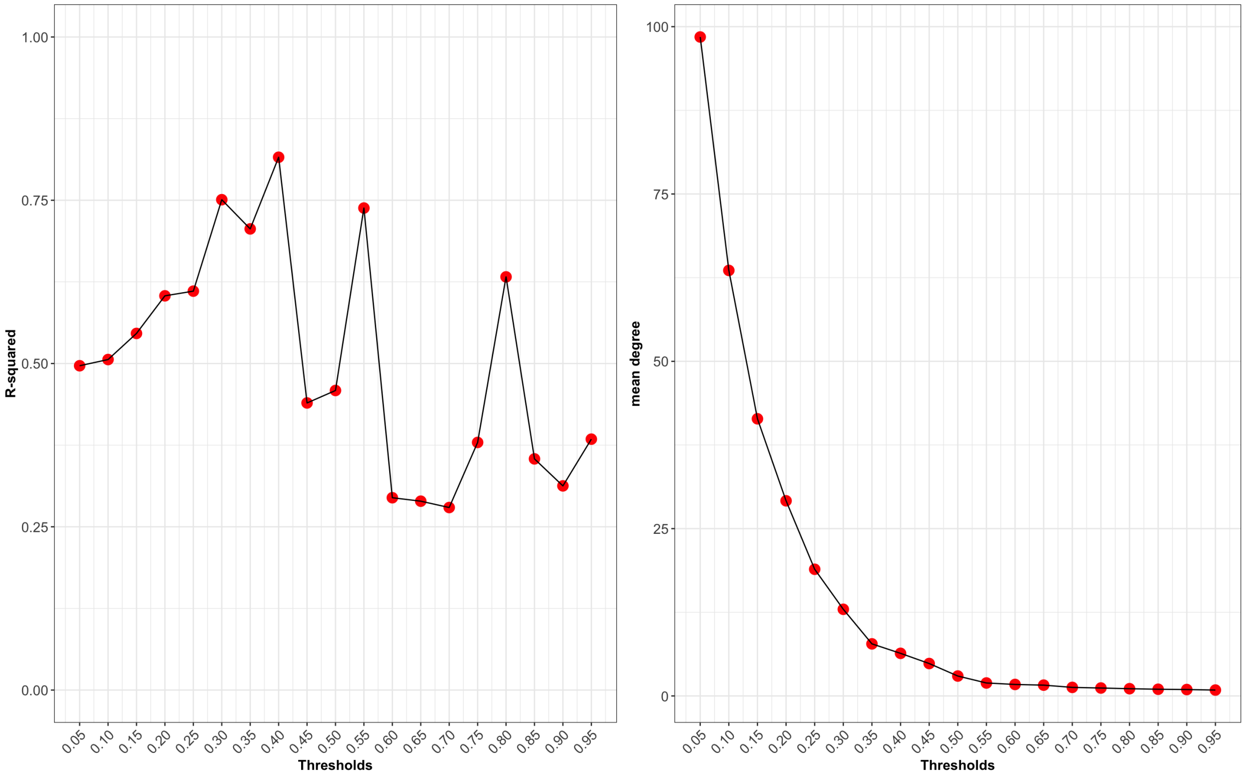

Choose tau that maximizes goodness of fit and binarize the information adjacency matrix

```
tau1=tau[which(R2==max(R2))]
I_unw = ifelse(I_adj_univ<tau1,0,1)
diag(I_unw)=0

R2

##[1] 0.49 0.55 0.57 0.60 0.62 0.75 0.72 0.89 0.19 0.22 0.83 0.13 0.21 0.20 0
.19
## [16] 0.70 0.15 0.24 0.34

which.max(R2)

## [1] 8

tau[8]

## [1] 0.4
```

```
net = network(I_unw, directed = FALSE)
#network.vertex.names(net)

#install.packages("GGally")
#library(GGally)

ggnet2(net,  palette = "Set2",size=1,  label = TRUE,
       label.size = 3,mode = "kamadakawai",legend.size = 0)
```

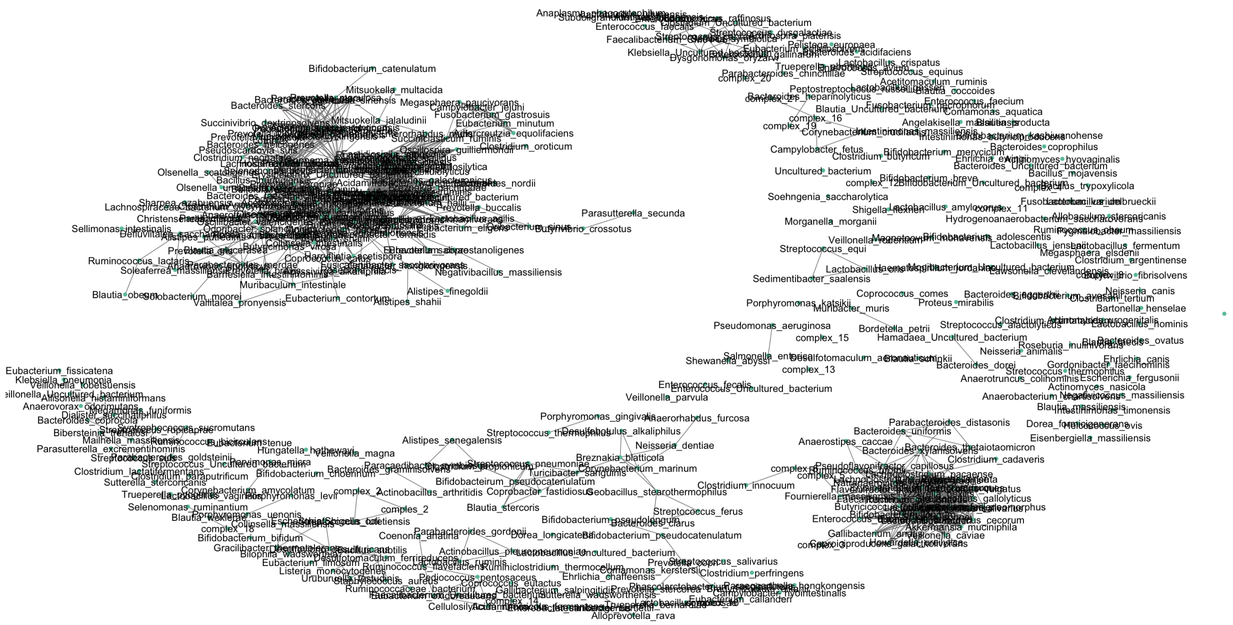

Choose ASVs using both unweighted and weighted network based on their 3 rd qurtile of degree

```
thresh_unw= summary(apply(I_unw,1,sum))[5]  ## based on 3rd quantile
deggrt1 = apply(I_unw,1,sum)[which(as.vector(apply(I_unw,1,sum))>thresh_unw)]

thresh_wnw= as.numeric(summary(apply(I_adj_univ,1,sum))[5])
deggrt2 = apply(I_adj_univ,1,sum)[which(as.vector(apply(I_adj_univ,1,sum))>th
resh_wnw)]



## unweighted network
output= data.frame(degree=as.vector(deggrt1)[order(as.vector(deggrt1))],
                   Taxonomy=names(deggrt1[order(as.vector(deggrt1))]))  ## 99
ASVs

## weighted network
output2= data.frame(degree=as.vector(deggrt2)[order(as.vector(deggrt2))],
```

```
                        Taxonomy=names(deggrt2[order(as.vector(deggrt2))])) ## 10
3 ASVs
```

Information Loss calculation based on weighted network

```
diag(I_adj_univ)=0
connect_w=sort(rowSums(I_adj_univ,na.rm=TRUE),decreasing=TRUE)
sort.connect = sort(connect_w)
vec= c(seq(0.01,1,0.05),0.99)


InfoLoss=c()
q2.data.ls=list()
for ( i in 1:length(vec)){
  #print(i)
  quantile(sort.connect, vec[i])
  q1.data=sort.connect[sort.connect > quantile(sort.connect, vec[i])]
  q2.data=names(sort.connect[sort.connect <= quantile(sort.connect, vec[i])])
  q2.data.ls[[i]]=q2.data
  names(q2.data.ls)[[i]]=vec[i]
  trunc.dta=dta[,names(q1.data)]

  if(is.vector(trunc.dta)==TRUE){
    trunc.mi = entropy(trunc.dta,method = "emp")} else{
      trunc.mi = mutinformation(trunc.dta,method = "emp")}

  info.filt =psych::tr(t(trunc.mi)%*%trunc.mi)

  ## norm of truncated MI for whole data
  results = mutinformation(dta, method="emp")
  info.all = psych::tr(t(results)%*%results)

  ## information loss
  InfoLos=round(1-(info.filt/info.all),2)

  InfoLoss=c(InfoLoss,InfoLos)
}

DInfloss = diff(InfoLoss)

p1=ggplot()+geom_line(aes(x=vec,y=InfoLoss),color="coral",size=1)+
  geom_point(aes(x=vec,y=InfoLoss),color="coral",size= 3)+theme_bw()+
  scale_x_continuous(breaks = vec,limits=c(0, 1))+
  theme(axis.text.x = element_text(angle = 60, hjust = 1))+
  ylab("Information loss")+
  xlab("Cutoff")+
  theme_bw()+
  theme(axis.text.x=element_text(angle=45, hjust=1,size=12)) +
  theme(axis.text.y=element_text(hjust=1,size=12),
```

```
        axis.title=element_text(size=12,face="bold"),
        legend.text=element_text(size=10),
        legend.title=element_text(size=12,face="bold")) +
  theme(plot.title = element_text(size=14))+
  xlab("Thresholds")+
  theme(legend.position = "none")

name=c()
for ( i in 1: length(vec)-1){name=c(name,paste0(vec[i+1],"-", vec[i]))}
name=name[-1]

p2=ggplot()+geom_line(aes(x=as.character(vec[-1]),y=DInfloss),group=1,color="
coral",size=1)+
  geom_point(aes(x=as.character(vec[-1]),y=DInfloss,group=1),color="coral",si
ze=3)+theme_bw()+
  scale_x_discrete(breaks=as.character(vec[-1]),
                   labels=name)+
  theme(axis.text.x=element_text(angle=45, hjust=1,size=12)) +
  theme(axis.text.y=element_text(hjust=1,size=12),
        axis.title=element_text(size=12,face="bold"),
        legend.text=element_text(size=10),
        legend.title=element_text(size=12,face="bold")) +
  theme(plot.title = element_text(size=14))+
  xlab("Thresholds")+
  theme(legend.position = "none") +
  ylab("Difference Information loss")+xlab("Diff. Threshold")

grid.arrange(p1,p2,ncol=2)
```

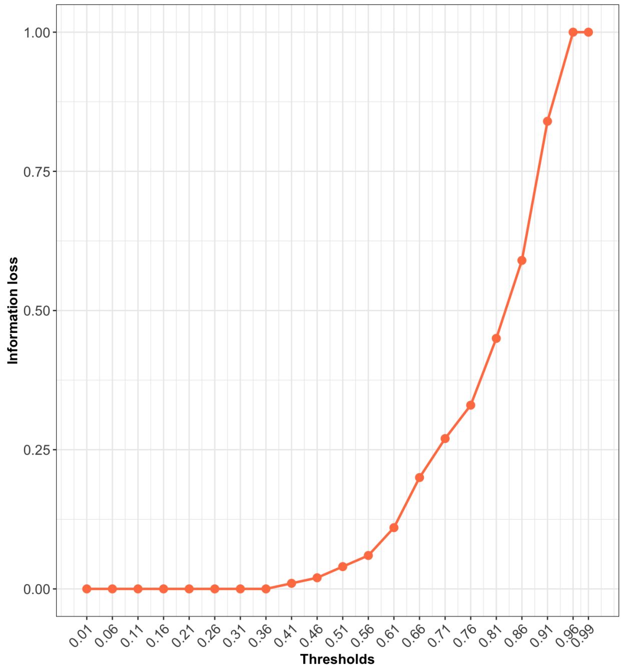

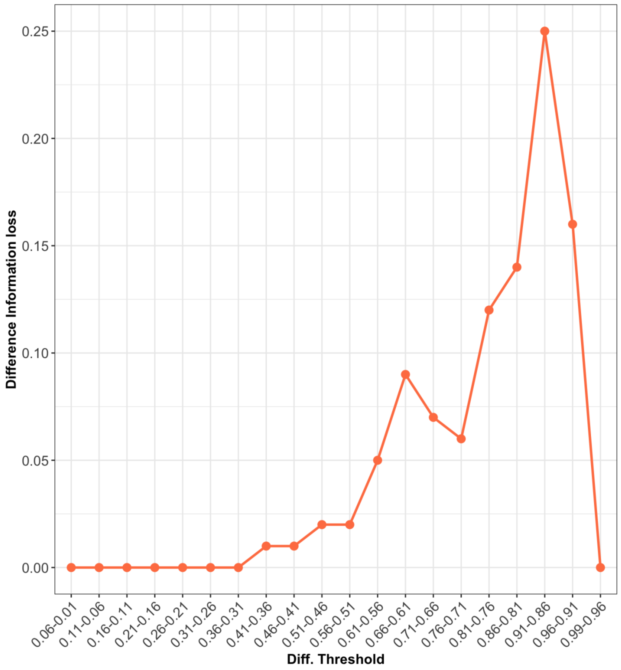

Permutation Test

```
PS = dta
cutoff<- c(seq(0.01,1,0.05))
num = 100
y = InformationLoss(ps=PS,cutoff,sort.connect)
```

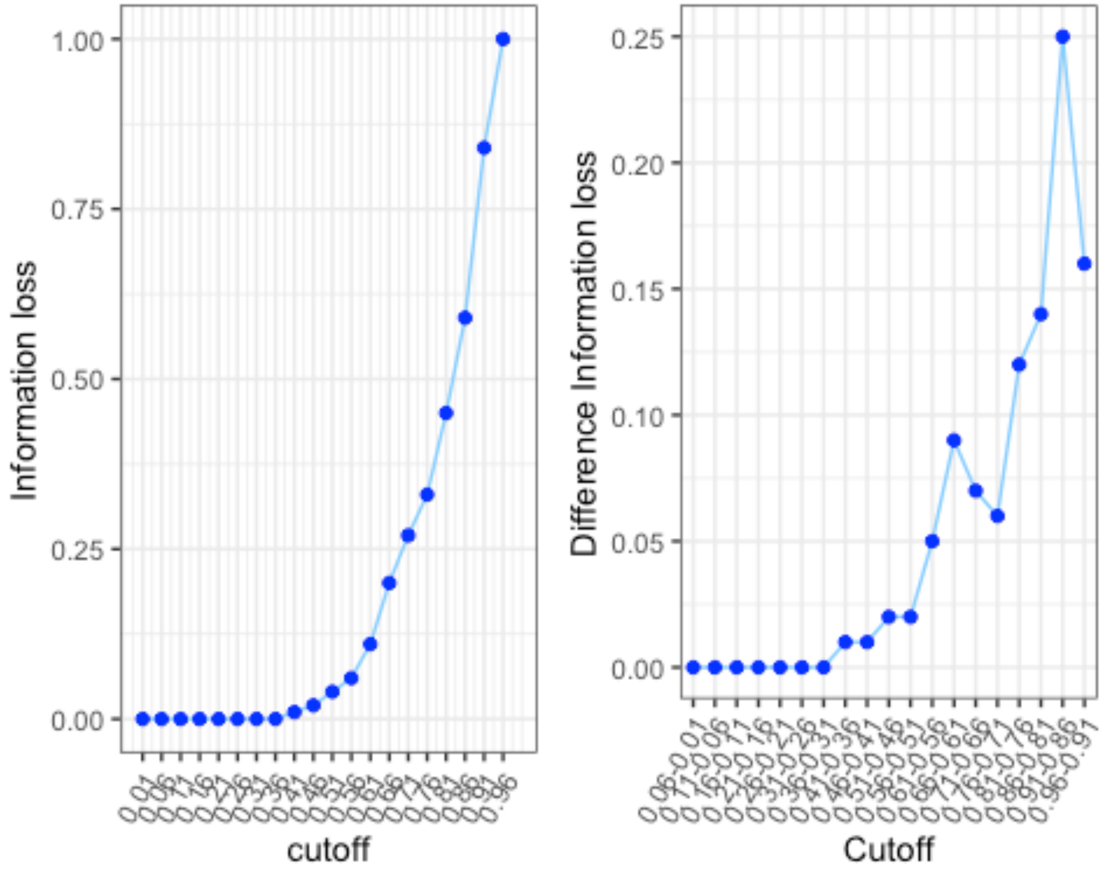


```
res=perm.mi(ps=PS,Num=y$num,M=num,y=y,cutoff,prbar = FALSE)

Pval = res$p_vals
Pval

##             X.pvalsperm2.
## 0.06-0.01          1.00
## 0.11-0.06          1.00
## 0.16-0.11          1.00
## 0.21-0.16          1.00
## 0.26-0.21          1.00
## 0.31-0.26          1.00
## 0.36-0.31          1.00
## 0.41-0.36          0.94
## 0.46-0.41          1.00
## 0.51-0.46          0.84
## 0.56-0.51          0.87
```

```
## 0.61-0.56          0.45
## 0.66-0.61          0.04
## 0.71-0.66          0.08
## 0.76-0.71          0.05
## 0.81-0.76          0.00
## 0.86-0.81          0.00
## 0.91-0.86          0.00
## 0.96-0.91          0.00
```

```
## benjamin-hochberg method to FDR in multiple testing.
Pval_ord= sort(Pval)
bh = (c(1:length(Pval_ord$X.pvalsperm2.)/length(Pval_ord$X.pvalsperm2.)))*0.1

cbind(Pval_ord$X.pvalsperm2.,bh,c(1:length(Pval_ord$X.pvalsperm2.)))
```

```
##                      bh
##  [1,] 0.00 0.005263158  1
##  [2,] 0.00 0.010526316  2
##  [3,] 0.00 0.015789474  3
##  [4,] 0.00 0.021052632  4
##  [5,] 0.04 0.026315789  5
##  [6,] 0.05 0.031578947  6
##  [7,] 0.08 0.036842105  7
##  [8,] 0.45 0.042105263  8
##  [9,] 0.84 0.047368421  9
## [10,] 0.87 0.052631579 10
## [11,] 0.94 0.057894737 11
## [12,] 1.00 0.063157895 12
## [13,] 1.00 0.068421053 13
## [14,] 1.00 0.073684211 14
## [15,] 1.00 0.078947368 15
## [16,] 1.00 0.084210526 16
## [17,] 1.00 0.089473684 17
## [18,] 1.00 0.094736842 18
## [19,] 1.00 0.100000000 19
```

Remove ASVs with degree less than 76 percentile 5% sig. level after BH correction

```
Pval <- cbind(Pval, BH_pval = p.adjust(Pval$X.pvalsperm2., method = "BH"))


outy=data.frame(colnames(dta)[-which(colnames(dta)%in%q2.data.ls$`0.76`)])
colnames(outy) = "Taxa to keep"
outy$num = c(1:dim(outy)[1])
outy = data.frame(outy$num,outy$`Taxa to keep`)
colnames(outy) = c("number","Taxa To Keep")
outy
```

```
##    number                        Taxa To Keep
## 1       1             Bifidobacterium_longum
## 2       2                          complex_1
```

```
## 3        3                      Escherichia_coli
## 4        4               Lactobacillus_johnsonii
## 5        5          Faecalibacterium_prausnitzii
## 6        6                 Lactobacillus_reuteri
## 7        7           Streptococcus_gallolyticus
## 8        8                  Tyzzerella_nexilis
## 9        9        Butyricicoccus_pullicaecorum
## 10      10             Collinsella_aerofaciens
## 11      11                Ruminococcus_torques
## 12      12                 Ruminococcus_gnavus
## 13      13            Lactobacillus_salivarius
## 14      14                     Shigella_sonnei
## 15      15                Bacteroides_fragilis
## 16      16                Bacteroides_vulgatus
## 17      17   Bifidobacterium_pseudocatenulatum
## 18      18        Bifidobacterium_pseudolongum
## 19      19           Faecalicoccus_pleomorphus
## 20      20      Erysipelatoclostridium_ramosum
## 21      21           Bacteroides_xylanisolvens
## 22      22          Natranaerovirga_hydrolytica
## 23      23               Prevotella_stercorea
## 24      24                    Prevotella_copri
## 25      25  Lachnoclostridium_urinimassiliense
## 26      26                  Alloprevotella_rava
## 27      27                 Ruminococcus_bromii
## 28      28            Subdoligranulum_variabile
## 29      29               Dorea_formicigenerans
## 30      30      Pseudoflavonifractor_capillosus
## 31      31           Fournierella_massiliensis
## 32      32               Bacteroides_uniformis
## 33      33        Bacteroides_thetaiotaomicron
## 34      34 Phascolarctobacterium_succinatutens
## 35      35         Lachnoclostridium_pacaense
## 36      36              Flavonifractor_plautii
## 37      37                       Blautia_obeum
## 38      38        Barnesiella_intestinihominis
## 39      39                  Olsenella_umbonata
## 40      40             Sellimonas_intestinalis
## 41      41                  Blautia_glucerasea
## 42      42               Ruminococcus_lactaris
## 43      43        Eubacterium_coprostanoligenes
## 44      44           Sutterella_wadsworthensis
## 45      45         Parabacteroides_distasonis
## 46      46                 Eubacterium_limosum
## 47      47           Parabacteroides_johnsonii
## 48      48                 Sharpea_azabuensis
## 49      49                   Eggerthella_lenta
## 50      50               Howardella_ureilytica
## 51      51             Muribaculum_intestinale
## 52      52     Parasutterella_excrementihominis
```

```
## 53     53            Lachnospiraceae_bacterium
## 54     54                Vallitalea_pronyensis
## 55     55               Roseimarinus_sediminis
## 56     56               Parabacteroides_merdae
## 57     57                Eubacterium_contortum
## 58     58                  Bacteroides_sartorii
## 59     59             Soleaferrea_massiliensis
## 60     60         Oscillibacter_valericigenes
## 61     61               Bacillus_thuringiensis
## 62     62                  Prevotella_baroniae
## 63     63             Odoribacter_splanchnicus
## 64     64       Negativibacillus_massiliensis
## 65     65                    Olsenella_profusa
## 66     66               Olsenella_scatoligenes
## 67     67                  Butyricimonas_virosa
## 68     68            Intestinimonas_timonensis
## 69     69                Pedobacter_alluvionis
## 70     70            Stoquefichus_massiliensis
## 71     71           Anaerotruncus_rubiinfantis
## 72     72                    Coprococcus_catus
## 73     73     Acetobacteroides_hydrogenigenes
## 74     74         Anaerophaga_thermohalophila
## 75     75                     Alistipes_ihumii
## 76     76                  Desulfovibrio_piger
## 77     77                     Roseburia_faecis
## 78     78          Bacteroides_faecichinchillae
## 79     79         Defluviitalea_saccharophila
## 80     80               Christensenella_minuta
## 81     81                  Mycoplasma_arginini
## 82     82            Faecalitalea_cylindroides
## 83     83      Anaerosolibacter_carboniphilus
## 84     84                      Bacillus_cereus
## 85     85                Alistipes_putredinis
## 86     86             Robinsoniella_peoriensis
## 87     87                  Pedobacter_arcticus
## 88     88            Oscillibacter_ruminantium
## 89     89             Collinsella_intestinalis
## 90     90             Butyricicoccus_desmolans
## 91     91                    Eubacterium_sulci
## 92     92           Prevotella_conceptionensis
## 93     93                  Eubacterium_nodatum
## 94     94             Treponema_succinifaciens
## 95     95                Holdemania_filiformis
## 96     96                  Phocea_massiliensis
## 97     97              Mogibacterium_neglectum
## 98     98            Ethanoligenens_harbinense
## 99     99         Sporanaerobacter_acetigenes
```

Shared ASVs that were chosen to keep based on permutation test on weighted network as well as based on unweighted network with power-law distribution fit

```
shrd = as.character(outy$`Taxa To Keep`)[which((as.character(outy$`Taxa To Ke
ep`)%in%as.character(output$Taxonomy)))]
shrd= data.frame(shrd)
shrd$num = c(1:dim(shrd)[1])
shrd = data.frame(shrd$num,shrd$shrd)
colnames(shrd) = c("number","Taxa shared")

shrd

##    number                     Taxa shared
## 1       1           Bifidobacterium_longum
## 2       2                        complex_1
## 3       3                 Escherichia_coli
## 4       4          Lactobacillus_johnsonii
## 5       5     Faecalibacterium_prausnitzii
## 6       6             Lactobacillus_reuteri
## 7       7       Streptococcus_gallolyticus
## 8       8              Tyzzerella_nexilis
## 9       9     Butyricicoccus_pullicaecorum
## 10     10           Collinsella_aerofaciens
## 11     11             Ruminococcus_torques
## 12     12              Ruminococcus_gnavus
## 13     13          Lactobacillus_salivarius
## 14     14                  Shigella_sonnei
## 15     15             Bacteroides_fragilis
## 16     16             Bacteroides_vulgatus
## 17     17        Faecalicoccus_pleomorphus
## 18     18   Erysipelatoclostridium_ramosum
## 19     19      Natranaerovirga_hydrolytica
## 20     20 Lachnoclostridium_urinimassiliense
## 21     21    Pseudoflavonifractor_capillosus
## 22     22         Lachnoclostridium_pacaense
## 23     23           Flavonifractor_plautii
## 24     24               Eggerthella_lenta
## 25     25            Howardella_ureilytica
## 26     26           Roseimarinus_sediminis
## 27     27             Bacteroides_sartorii
## 28     28              Prevotella_baroniae
## 29     29         Odoribacter_splanchnicus
## 30     30               Olsenella_profusa
## 31     31             Butyricimonas_virosa
## 32     32            Pedobacter_alluvionis
## 33     33        Stoquefichus_massiliensis
## 34     34         Anaerotruncus_rubiinfantis
## 35     35    Acetobacteroides_hydrogenigenes
## 36     36      Anaerophaga_thermohalophila
## 37     37                Alistipes_ihumii
## 38     38              Desulfovibrio_piger
## 39     39      Bacteroides_faecichinchillae
## 40     40               Mycoplasma_arginini
```

```
## 41      41          Faecalitalea_cylindroides
## 42      42     Anaerosolibacter_carboniphilus
## 43      43                   Bacillus_cereus
## 44      44           Robinsoniella_peoriensis
## 45      45                Pedobacter_arcticus
## 46      46          Oscillibacter_ruminantium
## 47      47           Collinsella_intestinalis
## 48      48           Butyricicoccus_desmolans
## 49      49                  Eubacterium_sulci
## 50      50         Prevotella_conceptionensis
## 51      51                Eubacterium_nodatum
## 52      52           Treponema_succinifaciens
## 53      53              Holdemania_filiformis
## 54      54                Phocea_massiliensis
## 55      55            Mogibacterium_neglectum
## 56      56          Ethanoligenens_harbinense
## 57      57        Sporanaerobacter_acetigenes
```

```
saveRDS(shrd, "shrd.rds")
```

Extract the 57 ASVs resulted from the filtering method from ASV count table

```
#Saving 57 ASVs after filtering the data
asvs <- readRDS("shrd.rds")

asvs = as.character(asvs$`Taxa shared`)

library("readxl")
data = read_excel("/Users/giovanaslanzon/Desktop/Analysis_Part1/analysisData.
xlsx")

output = data[,asvs]
rownames(output) = data$...1

library("openxlsx")
write.xlsx(output, file = "AnalysisData_output57.xlsx", row.names=T)
```

Merging the data with information of the calves

```
#Now I will merge analysisData(that is the shrd file) with metadata (that is
the MasterSheet)
library(openxlsx)
analysisData <- read.xlsx("AnalysisData_output57.xlsx")
names(analysisData)[1] <- "ZymoSample"
analysisData <- merge(analysisData,metadata[,c("ZymoSample", "SampleType","Br
eed","SampleAge","FarmID")])
analysisData$TotalReads <- colSums(counttab[, -ncol(counttab)])[analysisData$
ZymoSample]
analysisData$Other <- analysisData$TotalReads - rowSums(analysisData[,c(2:58)
])
hist(analysisData$Other/analysisData$TotalReads,20, main = "", xlab = "Fracti
```

```
on of Other in Sample") #histogram of percentage of "other" after dimensional reduction
```

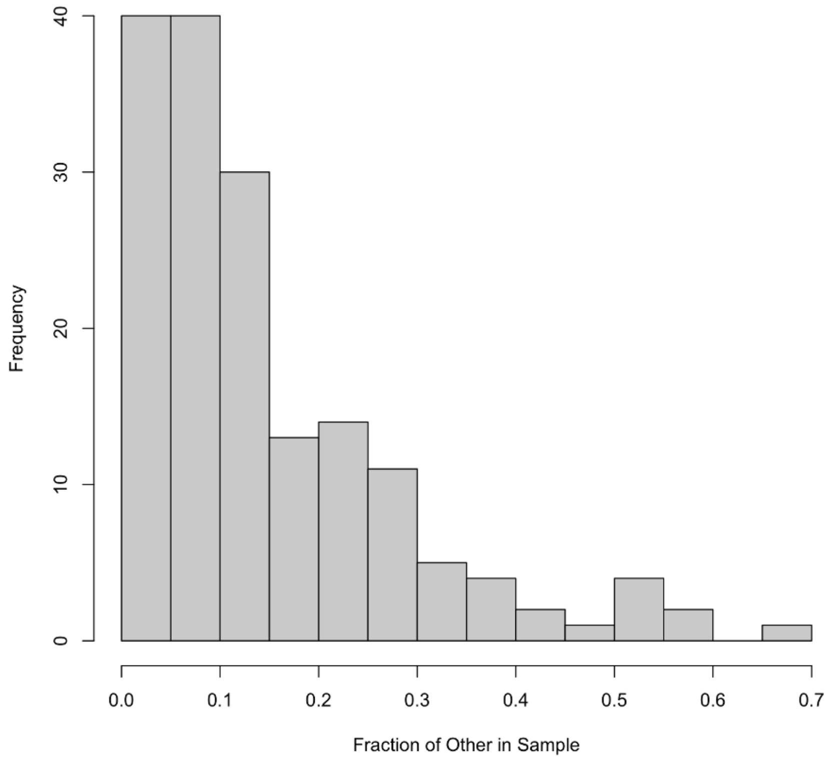

Data analysis

#Creating a dataset (temp) to access difference in the microbiome across health status

```
library("tidyverse")

library("readxl")
mergedASV_21<- read_excel("AnalysisData_output57.xlsx")
temp <- aggregate(mergedASV_21[,c(2:58,64),], by = mergedASV_21[,59,drop=F],sum)
temp$TotalReads - rowSums(temp[,2:58])

## [1] 130747 133240 527378

temp$Other <- temp$TotalReads - rowSums(temp[,2:58])
temp$TotalReads <- NULL
rowSums(temp[,-1])

## [1] 1114561  919291 3581830
```

```r
temp$Other
```

```
## [1] 130747 133240 527378
```

```r
rownames(temp) <- temp[,1]
temp <- temp[,-1]
xsq <- chisq.test(temp)

xsq # To get chi squared test - pvalues, df, x-squared
```

```
##
##  Pearson's Chi-squared test
##
## data:  temp
## X-squared = 1063615, df = 114, p-value < 2.2e-16
```

```r
xsq$residuals
```

```
##    Bifidobacterium_longum  complex_1 Escherichia_coli Lactobacillus_johnso
nii
## BS              -189.3482   15.70618         251.4912                -23.269
374
## DS              -357.2414  240.44499         126.9372                 34.956
191
## H                286.6057 -130.57324        -204.5962                 -4.728
884
##    Faecalibacterium_prausnitzii Lactobacillus_reuteri
## BS                     7.177462              24.52495
## DS                  -156.283418             220.82048
## H                     75.171009            -125.55060
##    Streptococcus_gallolyticus Tyzzerella_nexilis Butyricicoccus_pullicaeco
rum
## BS                  -12.11451         169.470484                     25.75
056
## DS                  450.22146          -5.368387                    -96.42
426
## H                  -221.32903         -91.815511                     34.48
517
##    Collinsella_aerofaciens Ruminococcus_torques Ruminococcus_gnavus
## BS               -88.47813            101.62659           59.021382
## DS              -101.90887            -62.08923          -47.898754
## H                100.98356            -25.23500           -8.657711
##    Lactobacillus_salivarius Shigella_sonnei Bacteroides_fragilis
## BS                 52.44897       117.84708            -63.06031
## DS                206.76354        58.20423            -75.64593
## H                -134.00599       -95.22513             73.49974
##    Bacteroides_vulgatus Faecalicoccus_pleomorphus
## BS            -105.3659                 -58.37489
## DS            -106.6415                 -57.56539
## H              112.8016                  61.72630
##    Erysipelatoclostridium_ramosum Natranaerovirga_hydrolytica
```

```
## BS                      -23.48655                     38.047389
## DS                      -12.41662                    -45.350207
## H                        19.39181                      1.751026
##    Lachnoclostridium_urinimassiliense Pseudoflavonifractor_capillosus
## BS                           7.249855                       -37.27292
## DS                         -16.590259                       -41.85628
## H                            4.360632                        41.99665
##    Lachnoclostridium_pacaense Flavonifractor_plautii Eggerthella_lenta
## BS                  -19.62344               3.893371         -21.54808
## DS                  -28.48176             -20.990915         -14.32080
## H                    25.37564               8.462387          19.27516
##    Howardella_ureilytica Roseimarinus_sediminis Bacteroides_sartorii
## BS              7.471266              -19.23418            -15.34239
## DS             -2.948430              -17.46820            -13.93374
## H              -2.673968               19.57891             15.61737
##    Prevotella_baroniae Odoribacter_splanchnicus Olsenella_profusa
## BS           -11.80373                -12.39428          4.838739
## DS           -10.71998                -11.25630         -8.845842
## H             12.01529                 12.61642          1.782217
##    Butyricimonas_virosa Pedobacter_alluvionis Stoquefichus_massiliensis
## BS            -11.19091            -10.051007                 -9.031743
## DS            -10.16342             -9.128179                 -8.202499
## H              11.39148             10.231151                  9.193619
##    Anaerotruncus_rubiinfantis Acetobacteroides_hydrogenigenes
## BS                  -7.754825                       -9.586083
## DS                  -7.042820                       -8.705942
## H                    7.893815                        9.757894
##    Anaerophaga_thermohalophila Alistipes_ihumii Desulfovibrio_piger
## BS                   -6.455952        -8.797980           -8.775392
## DS                   -5.863203        -7.990198           -7.969684
## H                     6.571663         8.955666            8.932674
##    Bacteroides_faecichinchillae Mycoplasma_arginini Faecalitalea_cylindroi
des
## BS                    -8.358378           -5.599726                 -6.268
783
## DS                    -7.590958           -5.532814                 -5.693
218
## H                      8.508185            5.926658                  6.381
139
##    Anaerosolibacter_carboniphilus Bacillus_cereus Robinsoniella_peoriensis
## BS                      -4.839402       -5.438058                -5.271263
## DS                      -4.395075       -4.938765                -4.787285
## H                        4.926138        5.535524                 5.365740
##    Pedobacter_arcticus Oscillibacter_ruminantium Collinsella_intestinalis
## BS           -6.059496                 -5.859678                -4.960912
## DS           -5.503147                 -5.321674                -4.505428
## H             6.168101                  5.964701                 5.049826
##    Butyricicoccus_desmolans Eubacterium_sulci Prevotella_conceptionensis
## BS                -5.438058         -4.586734                  -4.672475
## DS                -4.938765         -4.165606                  -4.243474
```

```
## H                  5.535524          4.668943                     4.756220
##    Eubacterium_nodatum Treponema_succinifaciens Holdemania_filiformis
## BS           -4.155376                -4.249828              6.702294
## DS           -3.773852                -3.859632             -3.456899
## H             4.229853                 4.325998             -1.987419
##    Phocea_massiliensis Mogibacterium_neglectum Ethanoligenens_harbinense
## BS           -3.021549               -2.440121                 -1.941903
## DS           -2.744127               -2.216082                 -1.763608
## H             3.075704                2.483855                  1.976707
##    Sporanaerobacter_acetigenes     Other
## BS                   -1.890109 -66.40585
## DS                   -1.716570  10.26045
## H                     1.923986  31.84492
```

```r
sum(xsq$residuals^2)
```

```
## [1] 1063615
```

```r
colSums(xsq$residuals^2)
```

```
##              Bifidobacterium_longum                          complex_1
##                        245616.95284                        75109.84834
##                    Escherichia_coli           Lactobacillus_johnsonii
##                        121220.49864                         1785.76141
##       Faecalibacterium_prausnitzii             Lactobacillus_reuteri
##                         30126.70348                        65126.10915
##         Streptococcus_gallolyticus                 Tyzzerella_nexilis
##                        251832.66220                        37179.15249
##       Butyricicoccus_pullicaecorum          Collinsella_aerofaciens
##                         11149.95583                        28411.47625
##                Ruminococcus_torques               Ruminococcus_gnavus
##                         14819.84125                         5852.77016
##           Lactobacillus_salivarius                    Shigella_sonnei
##                         63459.66267                        26343.49155
##                Bacteroides_fragilis              Bacteroides_vulgatus
##                         15101.12089                        35198.58383
##          Faecalicoccus_pleomorphus     Erysipelatoclostridium_ramosum
##                         10531.53830                         1081.83272
##         Natranaerovirga_hydrolytica Lachnoclostridium_urinimassiliense
##                          3507.31120                          346.81219
##    Pseudoflavonifractor_capillosus        Lachnoclostridium_pacaense
##                          4904.93701                         1840.21313
##              Flavonifractor_plautii                  Eggerthella_lenta
##                           527.38883                         1040.93708
##              Howardella_ureilytica            Roseimarinus_sediminis
##                            71.66317                         1058.42548
##                Bacteroides_sartorii              Prevotella_baroniae
##                           673.44024                          398.61303
##           Odoribacter_splanchnicus                  Olsenella_profusa
##                           439.49642                          104.83861
##                Butyricimonas_virosa              Pedobacter_alluvionis
```

```
##                           358.29747                          289.02284
##          Stoquefichus_massiliensis          Anaerotruncus_rubiinfantis
##                           233.37600                          172.05092
##    Acetobacteroides_hydrogenigenes        Anaerophaga_thermohalophila
##                           262.90289                          119.24321
##                     Alistipes_ihumii                 Desulfovibrio_piger
##                           221.45168                          220.31603
##       Bacteroides_faecichinchillae                Mycoplasma_arginini
##                           199.87434                           97.09424
##          Faecalitalea_cylindroides     Anaerosolibacter_carboniphilus
##                           112.42932                           67.00333
##                    Bacillus_cereus          Robinsoniella_peoriensis
##                            84.60590                           79.49548
##                 Pedobacter_arcticus          Oscillibacter_ruminantium
##                           105.04759                           98.23370
##           Collinsella_intestinalis          Butyricicoccus_desmolans
##                            70.41028                           84.60590
##                   Eubacterium_sulci        Prevotella_conceptionensis
##                            60.18943                           62.46073
##                 Eubacterium_nodatum          Treponema_succinifaciens
##                            49.40076                           51.67206
##               Holdemania_filiformis               Phocea_massiliensis
##                            60.82073                           26.11994
##            Mogibacterium_neglectum          Ethanoligenens_harbinense
##                            17.03474                           10.78867
##        Sporanaerobacter_acetigenes                              Other
##                            10.22085                         5529.11331
```

```
sort(colSums(xsq$residuals^2),decreasing = T)
```

```
##         Streptococcus_gallolyticus              Bifidobacterium_longum
##                        251832.66220                        245616.95284
##                    Escherichia_coli                           complex_1
##                        121220.49864                         75109.84834
##               Lactobacillus_reuteri             Lactobacillus_salivarius
##                         65126.10915                         63459.66267
##                 Tyzzerella_nexilis                 Bacteroides_vulgatus
##                         37179.15249                         35198.58383
##       Faecalibacterium_prausnitzii              Collinsella_aerofaciens
##                         30126.70348                         28411.47625
##                     Shigella_sonnei                Bacteroides_fragilis
##                         26343.49155                         15101.12089
##               Ruminococcus_torques         Butyricicoccus_pullicaecorum
##                         14819.84125                         11149.95583
##          Faecalicoccus_pleomorphus                  Ruminococcus_gnavus
##                         10531.53830                          5852.77016
##                               Other     Pseudoflavonifractor_capillosus
##                          5529.11331                          4904.93701
##        Natranaerovirga_hydrolytica          Lachnoclostridium_pacaense
##                          3507.31120                          1840.21313
```

```
##             Lactobacillus_johnsonii     Erysipelatoclostridium_ramosum
##                          1785.76141                         1081.83272
##              Roseimarinus_sediminis                   Eggerthella_lenta
##                          1058.42548                         1040.93708
##                 Bacteroides_sartorii              Flavonifractor_plautii
##                           673.44024                          527.38883
##             Odoribacter_splanchnicus                Prevotella_baroniae
##                           439.49642                          398.61303
##                 Butyricimonas_virosa Lachnoclostridium_urinimassiliense
##                           358.29747                          346.81219
##               Pedobacter_alluvionis    Acetobacteroides_hydrogenigenes
##                           289.02284                          262.90289
##            Stoquefichus_massiliensis                   Alistipes_ihumii
##                           233.37600                          221.45168
##                   Desulfovibrio_piger       Bacteroides_faecichinchillae
##                           220.31603                          199.87434
##           Anaerotruncus_rubiinfantis       Anaerophaga_thermohalophila
##                           172.05092                          119.24321
##            Faecalitalea_cylindroides                Pedobacter_arcticus
##                           112.42932                          105.04759
##                     Olsenella_profusa          Oscillibacter_ruminantium
##                           104.83861                           98.23370
##                   Mycoplasma_arginini                    Bacillus_cereus
##                            97.09424                           84.60590
##             Butyricicoccus_desmolans          Robinsoniella_peoriensis
##                            84.60590                           79.49548
##               Howardella_ureilytica          Collinsella_intestinalis
##                            71.66317                           70.41028
##     Anaerosolibacter_carboniphilus         Prevotella_conceptionensis
##                            67.00333                           62.46073
##               Holdemania_filiformis                  Eubacterium_sulci
##                            60.82073                           60.18943
##             Treponema_succinifaciens                Eubacterium_nodatum
##                            51.67206                           49.40076
##                  Phocea_massiliensis             Mogibacterium_neglectum
##                            26.11994                           17.03474
##            Ethanoligenens_harbinense        Sporanaerobacter_acetigenes
##                            10.78867                           10.22085
```

```
qchisq(0.95,114)
```

```
## [1] 139.9208
```

```
chisq.test(temp[,"Bifidobacterium_longum"])
```

```
## 
##  Chi-squared test for given probabilities
## 
## data:  temp[, "Bifidobacterium_longum"]
## X-squared = 1525104, df = 2, p-value < 2.2e-16
```

```r
chisq.test(temp[,"Sporanaerobacter_acetigenes"])

##
##  Chi-squared test for given probabilities
##
## data:  temp[, "Sporanaerobacter_acetigenes"]
## X-squared = 36, df = 2, p-value = 1.523e-08

#Accounting for Other
chisq.test(temp[,c("Bifidobacterium_longum","Other")])

##
##  Pearson's Chi-squared test
##
## data:  temp[, c("Bifidobacterium_longum", "Other")]
## X-squared = 121081, df = 2, p-value < 2.2e-16

chisq.test(temp[,c("Sporanaerobacter_acetigenes","Other")]) # All of them wer
e different

##
##  Pearson's Chi-squared test
##
## data:  temp[, c("Sporanaerobacter_acetigenes", "Other")]
## X-squared = 9.0101, df = 2, p-value = 0.01105
```

β-diversity analysis

```r
library("phyloseq")
library("ggplot2")
library("readxl")
library("dplyr")
```

Read the data and create phyloseq objects

```r
analysisData_output57 <- read_excel("~/Desktop/Phyloseq/analysisData_output57
.xlsx")
ASV <- (t(analysisData_output57[,c(2:60)]))
otu_mat<- as.data.frame(ASV)

taxonomy_analysisData_output57 <- #read_excel("~/Desktop/Phyloseq/taxonomy_an
alysisData_output57.xlsx")
taxonomy <- (t(FAMILY_GENUS_ORDER_analysisData_output57))
tax_mat<- as.data.frame(taxonomy)

samplescalves <- read_excel("~/Desktop/Phyloseq/samples.xlsx")
samples_df <- as.data.frame(samplescalves)

library("openxlsx")
write.xlsx(otu_mat, file = "otu_mat.xlsx", row.names=T)
```

```
otu_mat <- read_excel("otu_mat.xlsx") #Write "OTU" in the header in excel bef
ore calling it

write.xlsx(tax_mat, file = "tax_mat.xlsx", row.names=T)
tax_mat <- read_excel("tax_mat.xlsx")

write.xlsx(samples_df, file = "samples_df.xlsx", row.names=T)
samples_df <- read_excel("samples_df.xlsx")
```

Phyloseq objects need to have row names

Define the row names from the otu column

```
    row.names(otu_mat) <- otu_mat$otu
```

Remove the column otu since it is now used as a row name

```
    otu_mat <- otu_mat %>% select (-otu)
```

Idem for the two other matrixes

```
  row.names(tax_mat) <- tax_mat$otu
  tax_mat <- tax_mat %>% select (-otu)

  row.names(samples_df) <- samples_df$sample
  samples_df <- samples_df %>% select (-sample)
```

Transform into matrixes otu and tax tables

```
  otu_mat <- as.matrix(otu_mat)
  colnames(otu_mat) <- as.character(otu_mat[1,]) # Making Zymo id as header
  class(otu_mat) <- "numeric"
  otu_mat = otu_mat[-1,] #Deleting 1st row that is NA
  tax_mat <- as.matrix(tax_mat)
```

Transform to phyloseq objects

```
OTU = otu_table(otu_mat, taxa_are_rows = TRUE)
TAX = tax_table(tax_mat)

meta.df <- samples_df %>% select(-sample) %>% as.data.frame
rownames(meta.df) <- samples_df$sample
sample.data <- sample_data(meta.df)

samples = sample_data(meta.df)


calves <- phyloseq(OTU, TAX, samples)
calves
```

```
phyloseq-class experiment-level object
otu_table()   OTU Table:         [ 58 taxa and 167 samples ]
```

```
sample_data() Sample Data:       [ 167 samples by 5 sample variables ]
tax_table()   Taxonomy Table:    [ 58 taxa by 5 taxonomic ranks ]
```

Normalize number of reads in each sample using median sequencing depth.

```
  total = median(sample_sums(calves))
  standf = function(x, t=total) round(t * (x / sum(x)))
  calves = transform_sample_counts(calves, standf)
```

Creating a MDS (PCoA) - AltGower - Grouped by disease state (SampleType)

```
library(vegan)

GP = calves
wh0 = genefilter_sample(GP, filterfun_sample(function(x) x > 5), A = 0.5 * ns
amples(GP))
GP1 = prune_taxa(wh0, GP)

ord = ordinate(GP1~SampleType, "NMDS", "altGower")
(ordplot <- plot_ordination(GP1, ord, "samples", color="SampleType"))

# get data from the plotted ordination
orddata <- ordplot$data

ggplot(orddata, aes(x=Axis.1, y=Axis.2, color=SampleType)) +
  geom_point() +
  coord_fixed() +
  labs(x="Axis 1",
       y=" Axis 2") +
  scale_color_manual(name=NULL,
                     breaks=c("H",
                              "BS",
                              "DS"),
                     values=c("lightgreen", "blue", "red"),
                     labels=c("Healthy",
                              "Brightsick",
                              "Depressedsick")
                     )+
  theme_classic() +
  theme(
    legend.key.size = unit(0.25, "cm"),
    legend.position = c(0.95, 0.95),
    legend.background = element_rect(fill="NA",
                                     color="black"),
    legend.margin = margin(t=-2, r=3, b=3, l=3)
  )

centroid <- orddata %>%
  group_by(SampleType) %>%
  summarize(Axis.1 = mean(Axis.1),
```

```
            Axis.2 = mean(Axis.2), .groups="drop")

centroid

#Plotting the centroid in the figure
ggplot(orddata, aes(x=Axis.1, y=Axis.2, color=SampleType)) +
  geom_point() +
  geom_point(data=centroid, mapping= aes(x=Axis.1, y=Axis.2, color=SampleType
)) +
  coord_fixed() +
  labs(x="Axis 1",
       y=" Axis 2") +
  scale_color_manual(name=NULL,
                     breaks=c("H",
                              "BS",
                              "DS"),
                     values=c("lightgreen", "blue", "red"),
                     labels=c("Healthy",
                              "Brightsick",
                              "Depressedsick")
                     )+
  theme_classic() +
  theme(
    legend.key.size = unit(0.25, "cm"),
    legend.position = c(0.95, 0.95),
    legend.background = element_rect(fill="NA",
                                     color="black"),
    legend.margin = margin(t=-2, r=3, b=3, l=3)
  )

#Changing the simbol of the centroid so I can see where it is
ggplot(orddata, aes(x=Axis.1, y=Axis.2, color=SampleType)) +
  geom_point() +
  geom_point(data=centroid,
             mapping= aes(x=Axis.1, y=Axis.2, color=SampleType),
             shape= 15, size=5, show.legend = FALSE) +
  coord_fixed() +
  labs(x="Axis 1",
       y=" Axis 2") +
  scale_color_manual(name=NULL,
                     breaks=c("H",
                              "BS",
                              "DS"),
                     values=c("lightgreen", "blue", "red"),
                     labels=c("Healthy",
                              "Brightsick",
                              "Depressedsick")
                     )+
  theme_classic() +
  theme(
```

```
    legend.key.size = unit(0.25, "cm"),
    legend.position = c(0.95, 0.95),
    legend.background = element_rect(fill="NA",
                                     color="black"),
    legend.margin = margin(t=-2, r=3, b=3, l=3)
  )

#Adding ellipses
ggplot(orddata, aes(x=Axis.1, y=Axis.2, color=SampleType)) +
  geom_point() +
  geom_point(data=centroid,
             mapping= aes(x=Axis.1, y=Axis.2, color=SampleType),
             shape= 15, size=5, show.legend = FALSE) +
  stat_ellipse(type = "t", level = 0.95) +    #changing normal (default) to t
distribution
  coord_fixed() +                            #level default is 95% Conf. inte
rval, I changed to 80%
  labs(x="Axis 1",
       y=" Axis 2") +
  scale_color_manual(name=NULL,
                     breaks=c("H",
                              "BS",
                              "DS"),
                     values=c("lightgreen", "blue", "red"),
                     labels=c("Healthy",
                              "Brightsick",
                              "Depressedsick")
                     )+
  theme_bw() +
  theme(
    legend.key.size = unit(0.25, "cm"),
    legend.position = c(0.95, 0.95),
    legend.background = element_rect(fill="NA",
                                     color="black"),
    legend.margin = margin(t=-2, r=3, b=3, l=3)
  )

#Changing colors
ggplot(orddata, aes(x=Axis.1, y=Axis.2, color=SampleType)) +
  geom_point() +
  geom_point(data=centroid,
             mapping= aes(x=Axis.1, y=Axis.2, color=SampleType),
             shape= 15, size=5, show.legend = FALSE) +
  stat_ellipse(type = "t", level = 0.95) +    #changing normal (default) to t
distribution, 95% Conf. interval
  coord_fixed() +
  labs(x="Axis 1 [34.2%]",
       y=" Axis 2 [21.8%]") +
  scale_color_manual(name=NULL,
                     breaks=c("H",
```

```
                                   "BS",
                                   "DS"),
                         values=c("lightgreen", "blue", "red"),
                         labels=c("Healthy",
                                   "Brightsick",
                                   "Depressedsick")
                         )+
  theme_classic() +
  ggtitle("MDS/PCoA on weighted-altGower distance") +
  theme(
    legend.key.size = unit(0.25, "cm"),
    legend.position = c(0.95, 0.95),
    legend.background = element_rect(fill="NA",
                                     color="black"),
    legend.margin = margin(t=-2, r=3, b=3, l=3)
  )
```

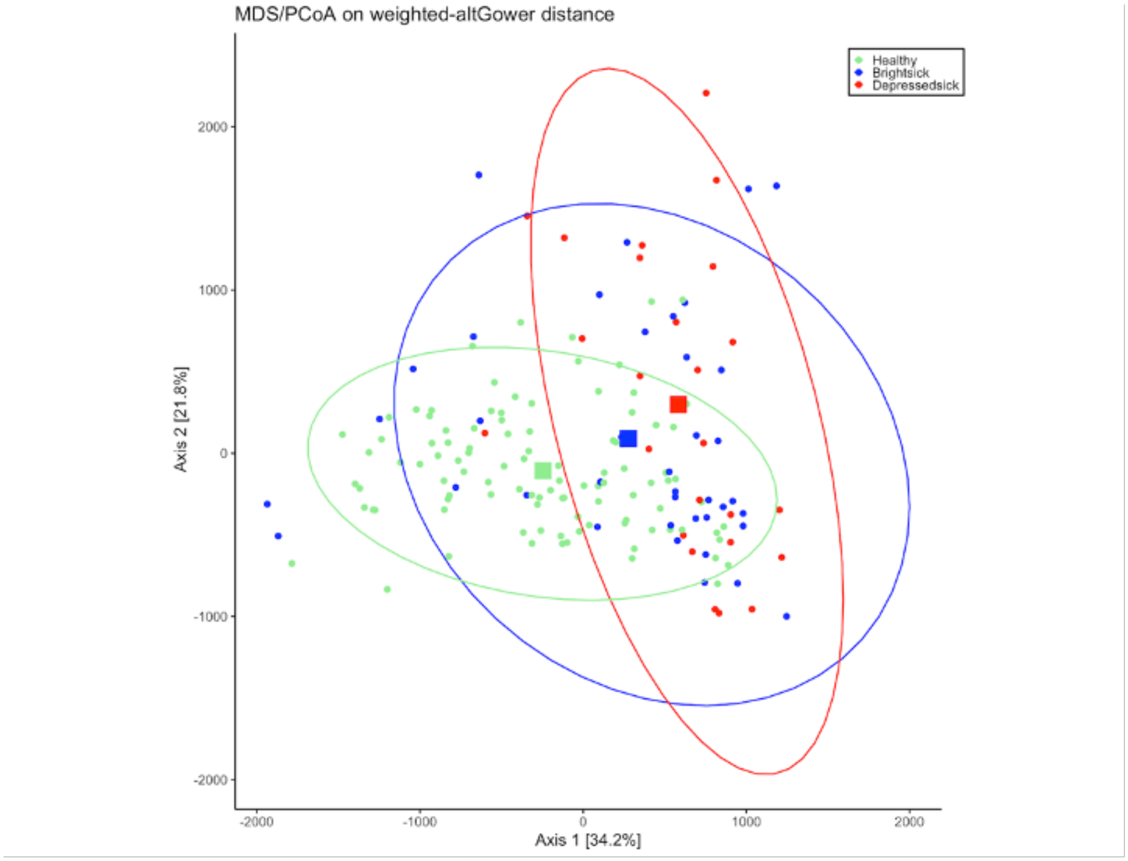

Creating a MDS (PCoA) - AltGower - Grouped by breeds

```
ggplot(orddata, aes(x=Axis.1, y=Axis.2, color=Breed)) +
  geom_point() +
  coord_fixed() +
  labs(x="Axis 1",
       y=" Axis 2") +
  scale_color_manual(name=NULL,
```

```
                     breaks=c("Holstein",
                              "Jersey",
                              "JerseyCross",
                              "BeefCross"),
                     values=c("darkolivegreen", "deepskyblue1","darkgoldenrod
", "darkmagenta"),
                     labels=c("Holstein",
                              "Jersey",
                              "JerseyCross",
                              "BeefCross")
                     )+
  theme_classic() +
  theme(
    legend.key.size = unit(0.25, "cm"),
    legend.position = c(0.95, 0.95),
    legend.background = element_rect(fill="NA",
                                     color="black"),
    legend.margin = margin(t=-2, r=3, b=3, l=3)
  )

centroid <- orddata %>%
  group_by(Breed) %>%
  summarize(Axis.1 = mean(Axis.1),
            Axis.2 = mean(Axis.2), .groups="drop")

centroid

#Plotting the centroid in the figure
ggplot(orddata, aes(x=Axis.1, y=Axis.2, color=Breed)) +
  geom_point() +
  geom_point(data=centroid, mapping= aes(x=Axis.1, y=Axis.2, color=Breed)) +
  coord_fixed() +
  labs(x="Axis 1",
       y=" Axis 2") +
  scale_color_manual(name=NULL,
                     breaks=c("Holstein",
                              "Jersey",
                              "JerseyCross",
                              "BeefCross"),
                     values=c("darkolivegreen", "deepskyblue1","darkgoldenrod
", "darkmagenta"),
                     labels=c("Holstein",
                              "Jersey",
                              "JerseyCross",
                              "BeefCross")
                     )+
  theme_classic() +
  theme(
    legend.key.size = unit(0.25, "cm"),
    legend.position = c(0.95, 0.95),
```

```
    legend.background = element_rect(fill="NA",
                                     color="black"),
    legend.margin = margin(t=-2, r=3, b=3, l=3)
  )

#Changing the simbol of the centroid so I can see where it is
ggplot(orddata, aes(x=Axis.1, y=Axis.2, color=Breed)) +
  geom_point() +
  geom_point(data=centroid,
             mapping= aes(x=Axis.1, y=Axis.2, color=Breed),
             shape= 15, size=5, show.legend = FALSE) +
  coord_fixed() +
  labs(x="Axis 1",
       y=" Axis 2") +
  scale_color_manual(name=NULL,
                     breaks=c("Holstein",
                              "Jersey",
                              "JerseyCross",
                              "BeefCross"),
                     values=c("darkolivegreen", "deepskyblue1","darkgoldenrod
", "darkmagenta"),
                     labels=c("Holstein",
                              "JerseyCross",
                              "JerseyCross",
                              "BeefCross")
                     )+
  theme_classic() +
  theme(
    legend.key.size = unit(0.25, "cm"),
    legend.position = c(0.95, 0.95),
    legend.background = element_rect(fill="NA",
                                     color="black"),
    legend.margin = margin(t=-2, r=3, b=3, l=3)
  )

#Adding ellipses
ggplot(orddata, aes(x=Axis.1, y=Axis.2, color=Breed)) +
  geom_point() +
  geom_point(data=centroid,
             mapping= aes(x=Axis.1, y=Axis.2, color=Breed),
             shape= 15, size=5, show.legend = FALSE) +
  stat_ellipse(type = "t", level = 0.95) +    #changing normal (default) to t
distribution
  coord_fixed() +                            #level default is 95% Conf. inte
rval, I changed to 80%
  labs(x="Axis 1 [34.2%]",
       y=" Axis 2 [21.8%]") +
  scale_color_manual(name=NULL,
                     breaks=c("Holstein",
                              "Jersey",
```

```
                                          "JerseyCross",
                                          "BeefCross"),
                               values=c("darkolivegreen", "deepskyblue1","darkgoldenrod
", "darkmagenta"),
                               labels=c("Holstein",
                                          "Jersey",
                                          "JerseyCross",
                                          "BeefCross")
                               )+
  theme_classic() +
  ggtitle("MDS/PCoA on weighted-altGower distance") +
  theme(
    legend.key.size = unit(0.25, "cm"),
    legend.position = c(0.95, 0.95),
    legend.background = element_rect(fill="NA",
                                     color="black"),
    legend.margin = margin(t=-2, r=3, b=3, l=3)
  )
```

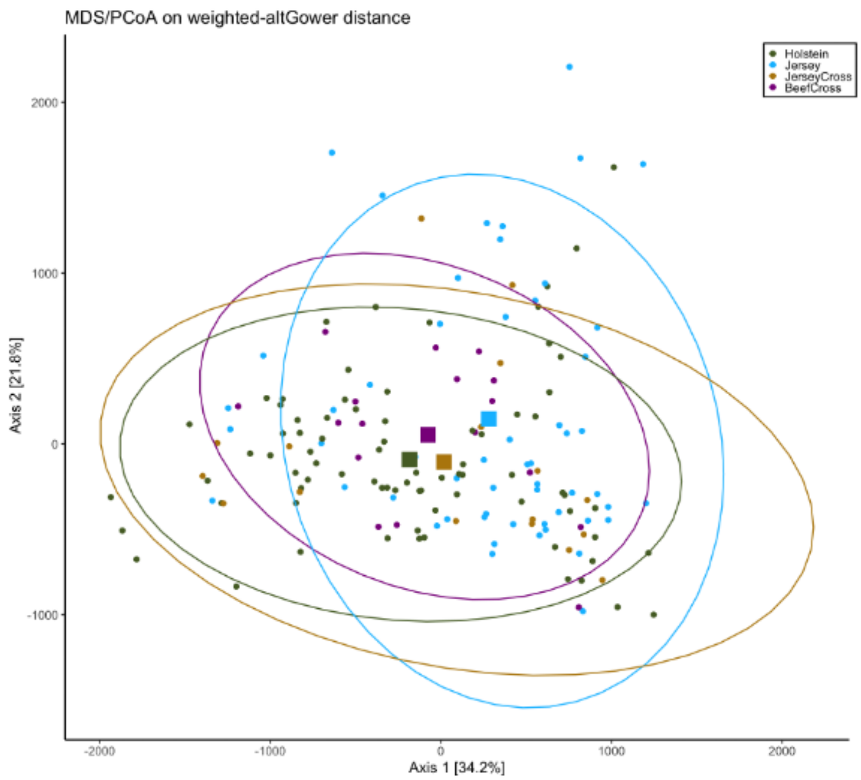

Relative abundance plots and heatmap

Creating a relative abundance plot at the phylum level

```
library(emmeans)
library("xlsx")
library(openxlsx)
library(readxl)
library(emmeans)
library(ggplot2)
library(magrittr)
library(dplyr)
library(tidyr)
library(lme4)
library("lmerTest")
library("car")
library(effects)
library("MASS")

phyla_counts <- aggregate(otu_mat, by = data.frame(tax_mat2[,"Phylum",drop=F]
), sum)

ds <- which(calves@sam_data[,"SampleType"] == "DS")
bs <- which(calves@sam_data[,"SampleType"] == "BS")
h <- which(calves@sam_data[,"SampleType"] == "H")

phyla_counts %>% transmute(Phylum, DS = rowSums(.[ds + 1]) / sum(.[ds + 1]) ,
                           BS = rowSums(.[bs + 1]) / sum(.[bs + 1]),
                           H = rowSums(.[h + 1]) / sum(.[h + 1])) %>% pivot_l
onger(cols = 2:4) -> phyla_perc

#Plotting the relative abundance
g <- ggplot(phyla_perc, aes(x = name, y = value))
g + geom_bar(aes(fill = Phylum), stat = "identity") + xlab("State") + ylab("R
elative Abundance") + theme_bw()


inner_join(
  phyla_counts %>% transmute(Phylum, DS = rowSums(.[ds + 1]),
                             BS = rowSums(.[bs + 1]),
                             H = rowSums(.[h + 1]) ) %>% pivot_longer(cols =
c(2:4), names_to = "Status", values_to = "Count")
  ,
  phyla_counts %>% transmute(Phylum, Tot_DS = sum(.[ds + 1]) ,
                             Tot_BS = sum(.[bs + 1]),
                             Tot_H = sum(.[h + 1])) %>%
pivot_longer(cols = c(2:4), names_prefix = "Tot_", names_to="Status", values_
to = "Total_Count")
) -> phyla_reads
```

```r
#Relative abundance plot
g <- ggplot(phyla_perc, aes(x = name, y = value))
g + geom_bar(aes(fill = Phylum), stat = "identity") + xlab("State") + ylab("R
elative Abundance") + theme_bw()

#At the phylum level- correcting x axis 0-100%
phyla_perc2 <- phyla_perc %>% mutate(perc = value * 100)
g <- ggplot(phyla_perc2, aes(x = name, y = perc))
g + geom_bar(aes(fill = Phylum), stat = "identity") + xlab("State") + ylab("R
elative Abundance (%)") + theme_bw() #S1 Fig - Empirical mean relative abunda
nce at the phylum level across disease states.
```

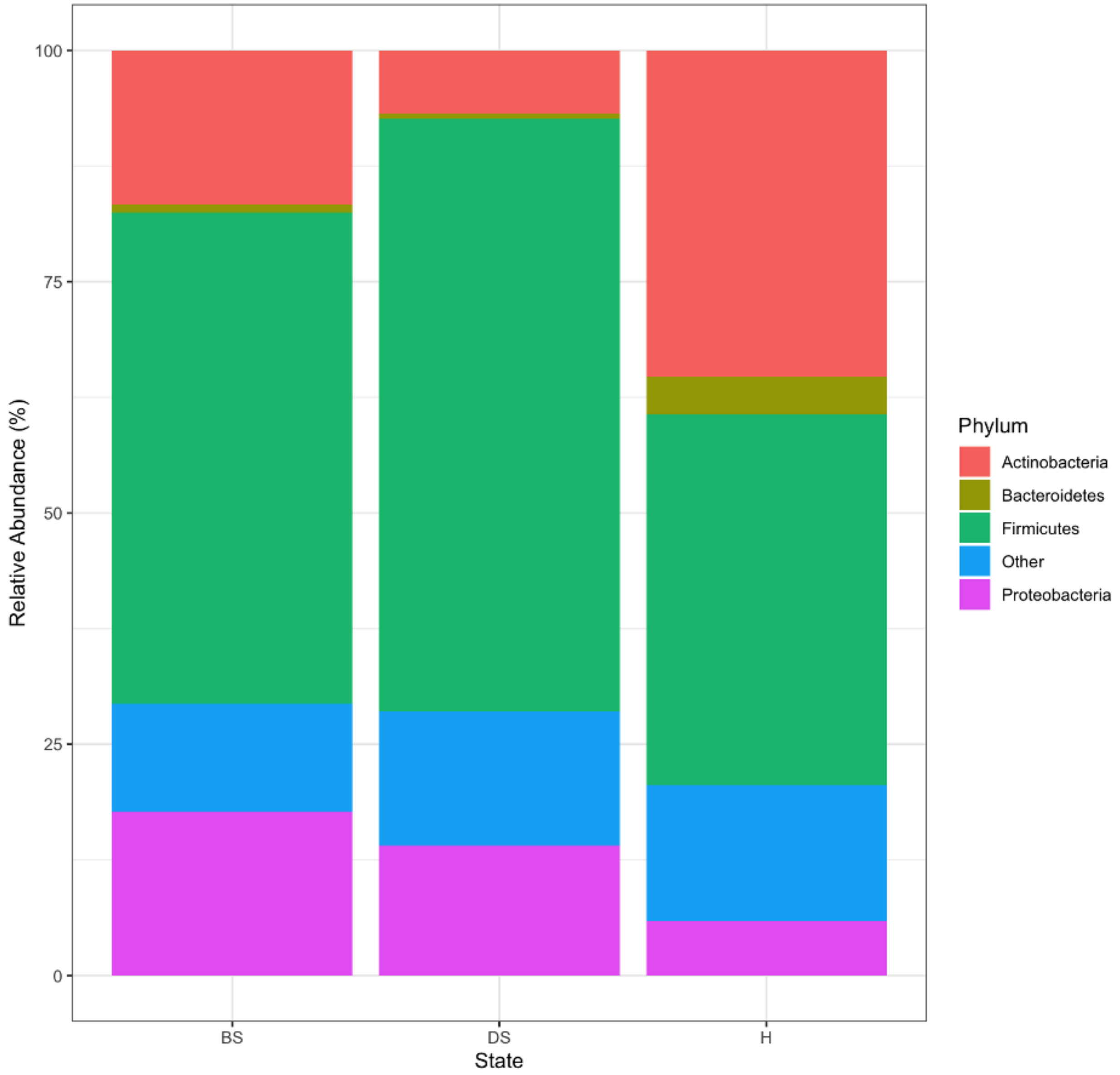

Relative abundance plot at the species level for relevant species according to the chi-square results

```
library(ggplot2)
library(dplyr)
library(openxlsx)
library(readxl)
library(tidyr)
library(stats)

#Species count results
mergedASV_21 %>%
  group_by(SampleType) %>%
  summarise(GroupReads = sum(TotalReads))

mergedASV_21 %>%
  group_by(SampleType) %>%
  summarise(GroupReads = sum(Bifidobacterium_longum))

mergedASV_21 %>%
  group_by(SampleType) %>%
  summarise(GroupReads = sum(complex_1))

mergedASV_21 %>%
  group_by(SampleType) %>%
  summarise(GroupReads = sum(Escherichia_coli))

mergedASV_21 %>%
  group_by(SampleType) %>%
  summarise(GroupReads = sum(Lactobacillus_johnsonii))

mergedASV_21 %>%
       group_by(SampleType) %>%
       summarise(GroupReads = sum(Faecalibacterium_prausnitzii))

mergedASV_21 %>%
       group_by(SampleType) %>%
       summarise(GroupReads = sum(Lactobacillus_reuteri))


mergedASV_21 %>%
       group_by(SampleType) %>%
       summarise(GroupReads = sum(Streptococcus_gallolyticus))

mergedASV_21 %>%
       group_by(SampleType) %>%
       summarise(GroupReads = sum(Tyzzerella_nexilis))

mergedASV_21 %>%
```

```
        group_by(SampleType) %>%
        summarise(GroupReads = sum(Butyricicoccus_pullicaecorum))

mergedASV_21 %>%
        group_by(SampleType) %>%
        summarise(GroupReads = sum(Collinsella_aerofaciens))

mergedASV_21 %>%
        group_by(SampleType) %>%
        summarise(GroupReads = sum(Lactobacillus_salivarius))

mergedASV_21 %>%
        group_by(SampleType) %>%
        summarise(GroupReads = sum(Shigella_sonnei))

mergedASV_21 %>%
        group_by(SampleType) %>%
        summarise(GroupReads = sum(Bacteroides_fragilis))

mergedASV_21 %>%
        group_by(SampleType) %>%
        summarise(GroupReads = sum(Bacteroides vulgatus ))

mergedASV_21 %>%
        group_by(SampleType) %>%
        summarise(GroupReads = sum( Eggerthella_lenta))

mergedASV_21 %>%
        group_by(SampleType) %>%
        summarise(GroupReads = sum( Others))


#Results saved in a file (species count)

species_count <- read_excel("Species_count.xlsx")

library(RColorBrewer)
nb.cols <- 25
mycolors <- colorRampPalette(brewer.pal(8, "Set1"))(nb.cols)
g <- ggplot(species_count, aes(x = Status, y = value))
g + geom_bar(aes(fill = Species), stat = "identity") + xlab("State") +
  ylab("Relative Abundance (%)") + theme_bw() +
  scale_fill_manual(values = mycolors)
```

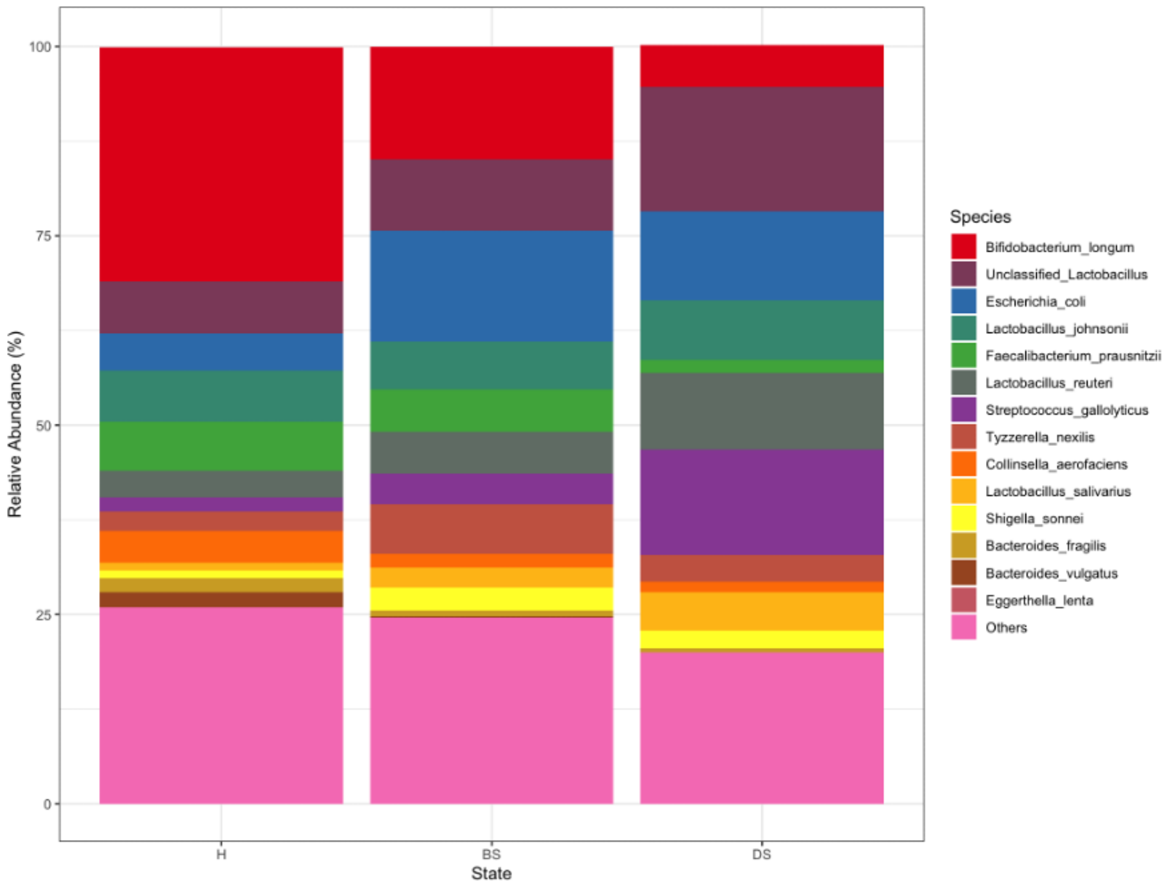

Creating a heatmap for relevant species according to the chi-square results

```
library("gplots")
library(cluster)
library(vegan)
library(RColorBrewer)

order_sampletype <- order(analysisData$SampleType)

order_sampletype2 <- analysisData[order_sampletype,]

#Organizing ASVs according to their residuals
matrixData <- as.matrix(order_sampletype2[,c(8, 2, 4, 3, 7, 14, 9, 17, 6, 11,
15, 16)]) # transform into a matrix
rownames(matrixData) <- rnames

matrixData <- prop.table(matrixData, 1)

dissMat <- vegdist(matrixData,method = "altGower")

matrixData <- log(matrixData)
matrixData[is.infinite(matrixData)] <- NA
```

```r
limits <- quantile(as.vector(matrixData),c(0.05,0.95), na.rm=T)

library(RColorBrewer)
Colors=c("gold", "orangered")
Colors=colorRampPalette(Colors)(100)
heatmap.2(matrixData,
          dendrogram = "none",
          Rowv = F,
          RowSideColors = c(    # grouping row-variables into different categ
ories
     rep("red", 25),
     rep("blue", 39),
     rep("lightgreen", 103)),
          Colv = F,
          tracecol = NA,
          breaks = seq(limits[1],limits[2],length=101),
          col = Colors,
          na.color = "gray70",
          symkey = F,
          key.title = NA,
          density = "none",
          key.xlab = "Log Frequency",
          cexRow=0.3,
          margins=c(12,2),
          rowsep=c(25, 64), sepcolor="black",  sepwidth=c(2,2))

#Adding a legend
par(lend = 1)           # square line ends  for the color legend
legend("topright",      # location of the legend on the heatmap plot
    legend = c("DepressedSick", "BrightSick", "Healthy"), # category labels
    col = c("red", "blue", "lightgreen"),  # color key
    lty = 1,            # line style
    lwd = 9,           # line width
    cex = 0.4
   )
```

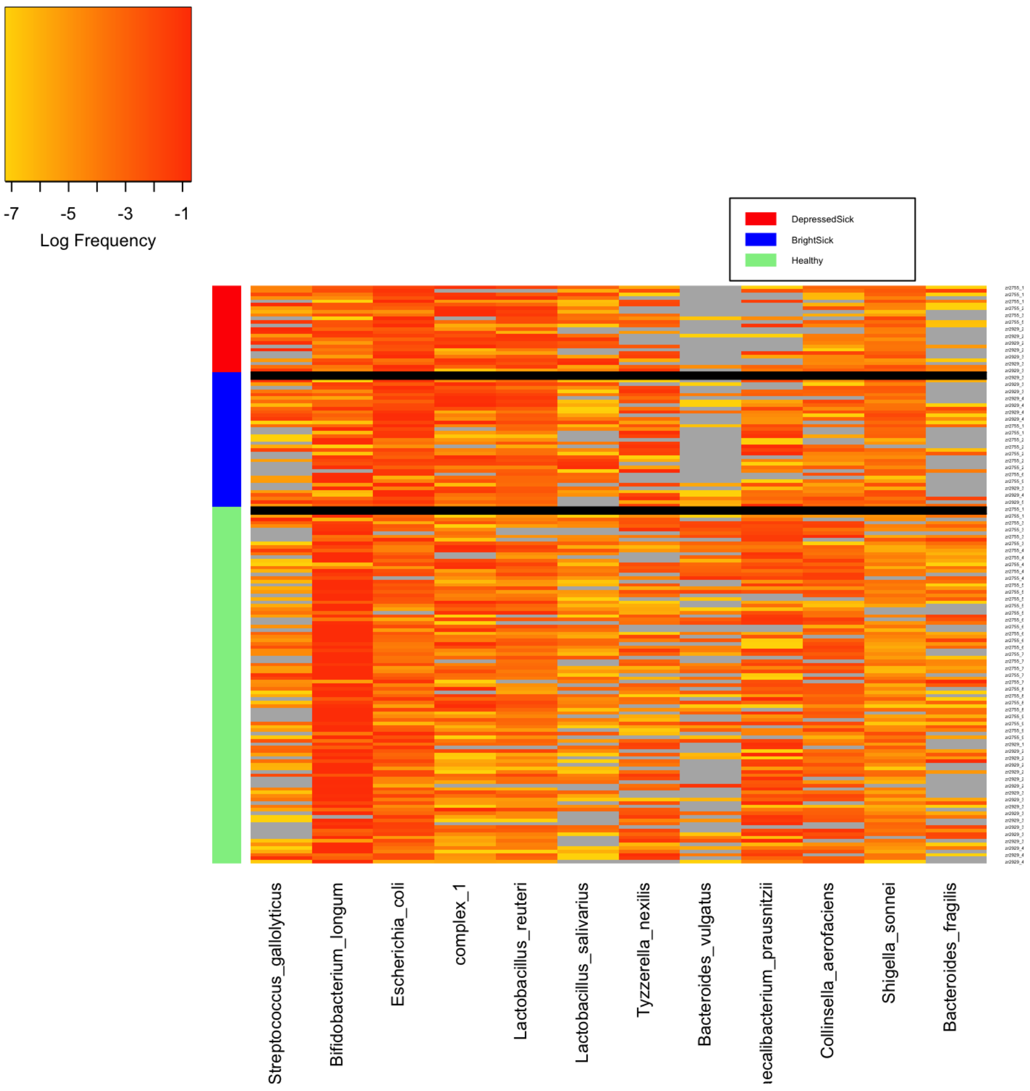

Linear mixed-effects model results

Linear mixed-effects model (LME) for relevant species according to the chi-square results

#Running lme models with the dataset mergedASV_24 dataset where healthy (= aH) Holstein (= aHolstein) to have the intercept as healthy Holstein calves.

```
library("readxl")
mergedASV_24<- read_excel("mergedASV_24.xlsx")

library ("emmeans")
library ("xlsx")
library ("openxlsx")
library ("readxl")
```

```r
library ("emmeans")
library ("ggplot2")
library ("magrittr")
library ("dplyr")
library ("tidyr")
library ("lme4")
library ("lmerTest")
library ("car")
library("effects")
library("MASS")

mergedASV_24 <- mergedASV_21 %>%
     mutate(SampleType = recode(SampleType, H = "aH"))

mergedASV_24 <- mergedASV_21 %>%
     mutate(Breed = recode(Breed, Holstein = "aHolstein"))


#Model for Species Bifidobacterium_longum
alice2.nbm <- glmer.nb(Bifidobacterium_longum ~ SampleType*Breed  + SampleAge
+ Period + AntibioticMilk + (1|FarmID) + offset(log(TotalReads)),
                      data = mergedASV_24)

## boundary (singular) fit: see ?isSingular

alice2.obj <- emmeans(alice2.nbm, pairwise ~ SampleType | Breed,type= "respon
se")
emmip(alice2.nbm, ~ SampleType | Breed, type = "response", CIs = T) + theme_b
w() + ylab("Normalized Read Counts") +
  scale_y_log10() #to plot
```

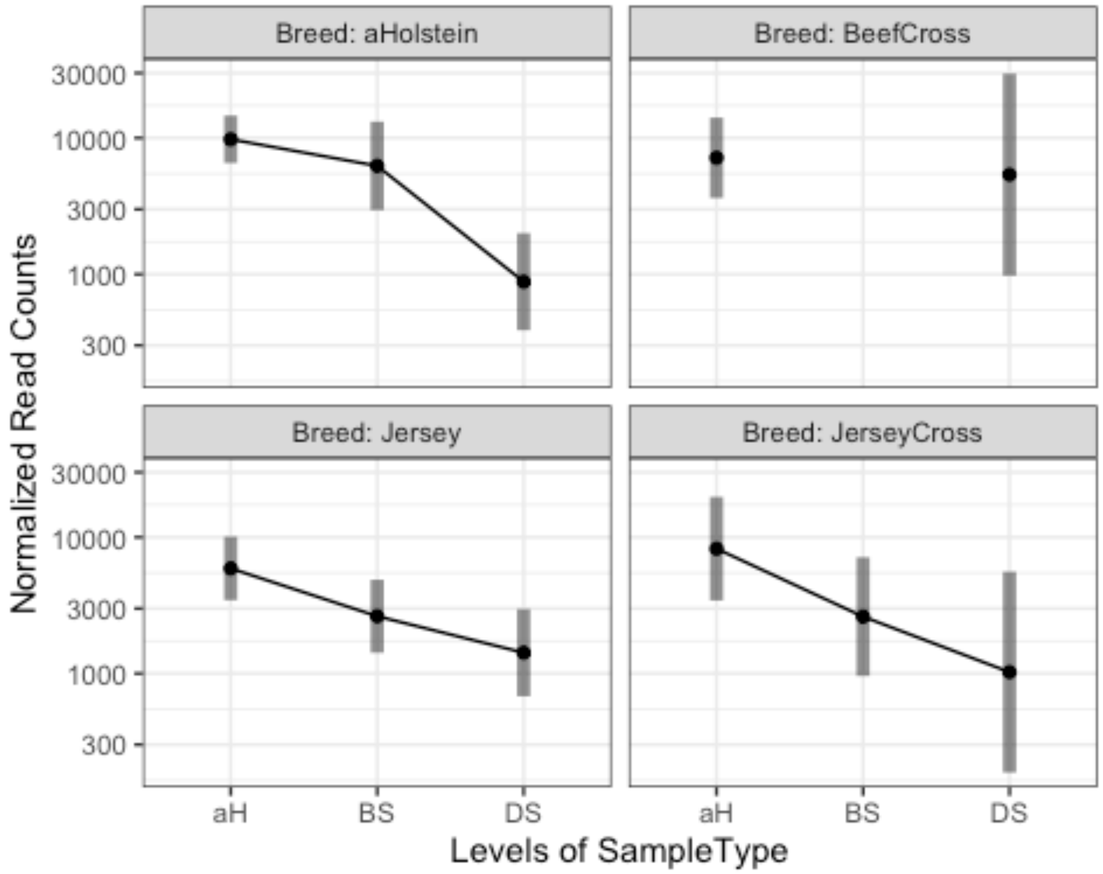


```
summary(alice2.nbm)

## Generalized linear mixed model fit by maximum likelihood (Laplace
##   Approximation) [glmerMod]
##  Family: Negative Binomial(0.6809)  ( log )
## Formula: Bifidobacterium_longum ~ SampleType * Breed + SampleAge + Period
+
##     AntibioticMilk + (1 | FarmID) + offset(log(TotalReads))
##    Data: mergedASV_24
##
##      AIC      BIC   logLik deviance df.resid
##   3210.2   3260.1  -1589.1   3178.2      151
##
## Scaled residuals:
##     Min      1Q  Median      3Q     Max
## -0.8251 -0.6779 -0.1721  0.4344  3.9214
##
## Random effects:
##  Groups Name        Variance  Std.Dev.
##  FarmID (Intercept) 1.816e-10 1.348e-05
## Number of obs: 167, groups:  FarmID, 2
##
## Fixed effects:
```

```
##                                  Estimate Std. Error z value Pr(>|z|)
## (Intercept)                      -2.66563    0.76410  -3.489 0.000486 ***
## SampleTypeBS                     -0.44930    0.41145  -1.092 0.274839
## SampleTypeDS                     -2.40735    0.44041  -5.466  4.6e-08 ***
## BreedBeefCross                   -0.31322    0.35561  -0.881 0.378427
## BreedJersey                      -0.50514    0.31346  -1.612 0.107070
## BreedJerseyCross                 -0.17155    0.47335  -0.362 0.717043
## SampleAge                         0.08824    0.04725   1.868 0.061825 .
## Period                            0.14133    0.24315   0.581 0.561063
## AntibioticMilkTRUE                0.76434    0.34728   2.201 0.027739 *
## SampleTypeDS:BreedBeefCross       2.12221    1.00899   2.103 0.035440 *
## SampleTypeBS:BreedJersey         -0.35933    0.53170  -0.676 0.499161
## SampleTypeDS:BreedJersey          0.98259    0.61636   1.594 0.110895
## SampleTypeBS:BreedJerseyCross -0.69923    0.78499  -0.891 0.373061
## SampleTypeDS:BreedJerseyCross  0.32068    1.03605   0.310 0.756927
## ---
## Signif. codes:  0 '***' 0.001 '**' 0.01 '*' 0.05 '.' 0.1 ' ' 1

##
## Correlation matrix not shown by default, as p = 14 > 12.
## Use print(x, correlation=TRUE)  or
##     vcov(x)        if you need it

## fit warnings:
## fixed-effect model matrix is rank deficient so dropping 1 column / coeffic
ient
## optimizer (Nelder_Mead) convergence code: 0 (OK)
## boundary (singular) fit: see ?isSingular
```

```
alice2.obj #to get pvalues
```

```
## $emmeans
## Breed = aHolstein:
##  SampleType response   SE  df asymp.LCL asymp.UCL
##  aH             9790 2002 Inf      6557     14616
##  BS             6246 2383 Inf      2958     13192
##  DS              882  369 Inf       388      2003
##
## Breed = BeefCross:
##  SampleType response   SE  df asymp.LCL asymp.UCL
##  aH             7157 2482 Inf      3627     14122
##  BS           nonEst   NA  NA        NA        NA
##  DS             5381 4688 Inf       976     29681
##
## Breed = Jersey:
##  SampleType response   SE  df asymp.LCL asymp.UCL
##  aH             5907 1620 Inf      3451     10112
##  BS             2632  829 Inf      1419      4879
##  DS             1421  534 Inf       680      2969
##
## Breed = JerseyCross:
```

```
##  SampleType response   SE  df asymp.LCL asymp.UCL
##  aH             8246 3686 Inf      3434     19804
##  BS             2615 1339 Inf       959      7131
##  DS             1023  886 Inf       187      5586
##
## Results are averaged over the levels of: Period, AntibioticMilk
## Confidence level used: 0.95
## Intervals are back-transformed from the log scale
##
## $contrasts
## Breed = aHolstein:
##  contrast  ratio    SE  df z.ratio p.value
##  aH / BS    1.57 0.645 Inf 1.092   0.5190
##  aH / DS   11.10 4.890 Inf 5.466   <.0001
##  BS / DS    7.09 3.993 Inf 3.474   0.0015
##
## Breed = BeefCross:
##  contrast  ratio    SE  df z.ratio p.value
##  aH / BS  nonEst    NA  NA     NA       NA
##  aH / DS    1.33 1.213 Inf 0.313   0.9476
##  BS / DS  nonEst    NA  NA     NA       NA
##
## Breed = Jersey:
##  contrast  ratio    SE  df z.ratio p.value
##  aH / BS    2.24 0.860 Inf 2.111   0.0875
##  aH / DS    4.16 1.847 Inf 3.207   0.0038
##  BS / DS    1.85 0.856 Inf 1.332   0.3772
##
## Breed = JerseyCross:
##  contrast  ratio    SE  df z.ratio p.value
##  aH / BS    3.15 2.107 Inf 1.719   0.1981
##  aH / DS    8.06 7.629 Inf 2.204   0.0705
##  BS / DS    2.56 2.525 Inf 0.949   0.6091
##
## Results are averaged over the levels of: Period, AntibioticMilk
## P value adjustment: tukey method for comparing a family of 3 estimates
## Tests are performed on the log scale

#Model for E coli
lindsey3.nbm <- glmer.nb(Escherichia_coli ~ SampleType*Breed  + SampleAge + P
eriod + AntibioticMilk + (1|FarmID) + offset(log(TotalReads)),
                         data = mergedASV_24)

## boundary (singular) fit: see ?isSingular

lindsey3.obj <- emmeans(lindsey3.nbm, pairwise ~ SampleType | Breed, type= "r
esponse")
emmip(lindsey3.nbm, ~ SampleType | Breed, type = "response", CIs = T) + theme
_bw() + ylab("Normalized Read Counts") +
  scale_y_log10() #to plot
```

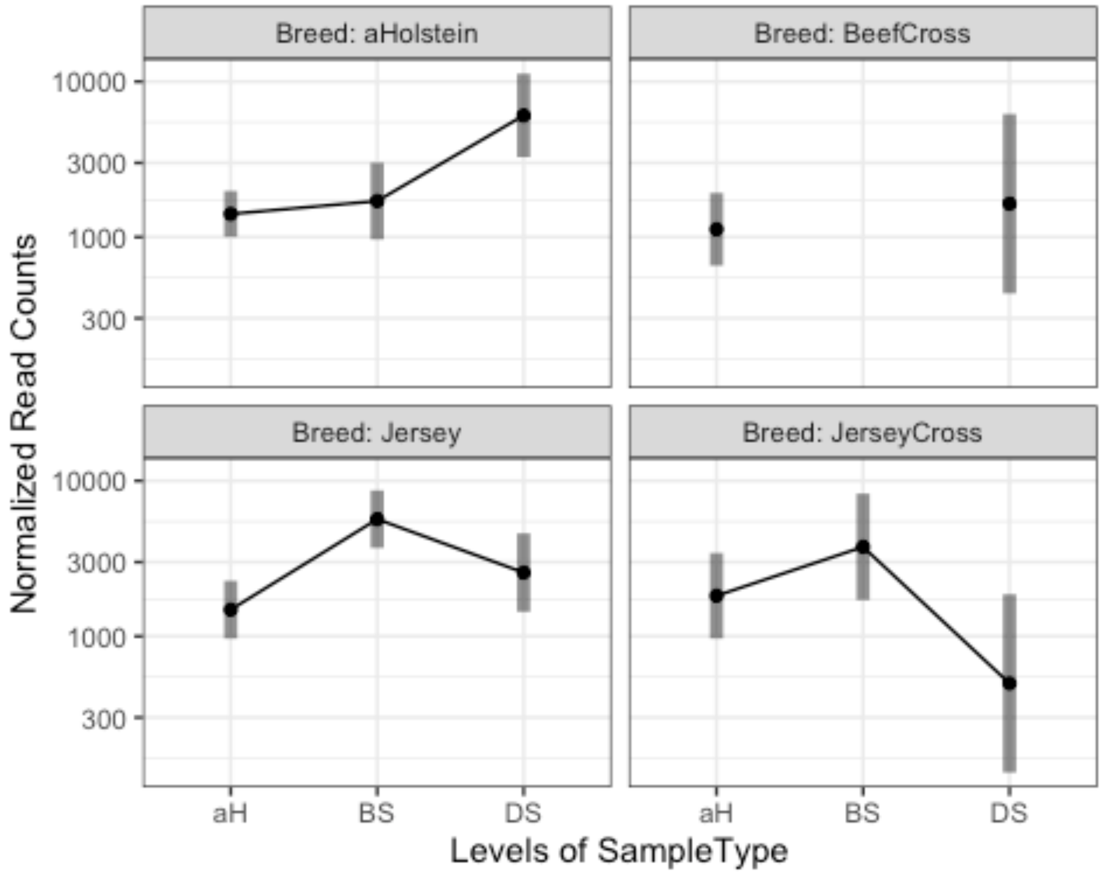


```r
summary(lindsey3.nbm)
```

```
## Generalized linear mixed model fit by maximum likelihood (Laplace
##   Approximation) [glmerMod]
##  Family: Negative Binomial(1.1358)  ( log )
## Formula: Escherichia_coli ~ SampleType * Breed + SampleAge + Period +
##     AntibioticMilk + (1 | FarmID) + offset(log(TotalReads))
##    Data: mergedASV_24
##
##      AIC      BIC   logLik deviance df.resid
##   2858.1   2908.0  -1413.1   2826.1      151
##
## Scaled residuals:
##     Min      1Q  Median      3Q     Max
## -1.0648 -0.6651 -0.2995  0.3685  6.3010
##
## Random effects:
##  Groups Name        Variance  Std.Dev.
##  FarmID (Intercept) 1.872e-12 1.368e-06
## Number of obs: 167, groups:  FarmID, 2
##
## Fixed effects:
##                               Estimate Std. Error z value Pr(>|z|)
```

```
## (Intercept)                       -1.61864    0.56496  -2.865 0.004169 **
## SampleTypeBS                       0.19157    0.30704   0.624 0.532683
## SampleTypeDS                       1.46032    0.34880   4.187 2.83e-05 ***
## BreedBeefCross                    -0.22691    0.28010  -0.810 0.417887
## BreedJersey                        0.05207    0.25395   0.205 0.837548
## BreedJerseyCross                   0.25870    0.34530   0.749 0.453738
## SampleAge                         -0.16774    0.03417  -4.909 9.15e-07 ***
## Period                            -0.04732    0.17824  -0.266 0.790624
## AntibioticMilkTRUE                 0.21571    0.27140   0.795 0.426717
## SampleTypeDS:BreedBeefCross       -1.08193    0.78870  -1.372 0.170132
## SampleTypeBS:BreedJersey           1.14977    0.42419   2.711 0.006717 **
## SampleTypeDS:BreedJersey          -0.91042    0.49979  -1.822 0.068513 .
## SampleTypeBS:BreedJerseyCross      0.53274    0.57854   0.921 0.357144
## SampleTypeDS:BreedJerseyCross     -2.75913    0.81784  -3.374 0.000742 ***
## ---
## Signif. codes:  0 '***' 0.001 '**' 0.01 '*' 0.05 '.' 0.1 ' ' 1

##
## Correlation matrix not shown by default, as p = 14 > 12.
## Use print(x, correlation=TRUE)  or
##     vcov(x)        if you need it

## fit warnings:
## fixed-effect model matrix is rank deficient so dropping 1 column / coeffic
ient
## optimizer (Nelder_Mead) convergence code: 0 (OK)
## boundary (singular) fit: see ?isSingular
```

```
lindsey3.obj #to get pvalues
```

```
## $emmeans
## Breed = aHolstein:
##  SampleType response   SE  df asymp.LCL asymp.UCL
##  aH             1407  244 Inf      1001      1978
##  BS             1704  494 Inf       965      3009
##  DS             6061 1912 Inf      3265     11249
##
## Breed = BeefCross:
##  SampleType response   SE  df asymp.LCL asymp.UCL
##  aH             1121  308 Inf       654      1922
##  BS           nonEst   NA  NA        NA        NA
##  DS             1637 1110 Inf       433      6185
##
## Breed = Jersey:
##  SampleType response   SE  df asymp.LCL asymp.UCL
##  aH             1482  323 Inf       967      2273
##  BS             5668 1233 Inf      3701      8682
##  DS             2569  762 Inf      1437      4593
##
## Breed = JerseyCross:
##  SampleType response   SE  df asymp.LCL asymp.UCL
```

```
##   aH              1822  589 Inf       967      3433
##   BS              3760 1513 Inf      1709      8275
##   DS               497  335 Inf       133      1862
##
## Results are averaged over the levels of: Period, AntibioticMilk
## Confidence level used: 0.95
## Intervals are back-transformed from the log scale
##
## $contrasts
## Breed = aHolstein:
##   contrast  ratio     SE  df z.ratio p.value
##   aH / BS   0.826 0.2535 Inf -0.624  0.8070
##   aH / DS   0.232 0.0810 Inf -4.187  0.0001
##   BS / DS   0.281 0.1197 Inf -2.980  0.0081
##
## Breed = BeefCross:
##   contrast  ratio     SE  df z.ratio p.value
##   aH / BS  nonEst     NA  NA      NA      NA
##   aH / DS   0.685 0.4846 Inf -0.535  0.8542
##   BS / DS  nonEst     NA  NA      NA      NA
##
## Breed = Jersey:
##   contrast  ratio     SE  df z.ratio p.value
##   aH / BS   0.261 0.0762 Inf -4.604  <.0001
##   aH / DS   0.577 0.1992 Inf -1.593  0.2486
##   BS / DS   2.207 0.7974 Inf  2.190  0.0729
##
## Breed = JerseyCross:
##   contrast  ratio     SE  df z.ratio p.value
##   aH / BS   0.485 0.2432 Inf -1.443  0.3187
##   aH / DS   3.665 2.6960 Inf  1.766  0.1813
##   BS / DS   7.562 5.8620 Inf  2.610  0.0246
##
## Results are averaged over the levels of: Period, AntibioticMilk
## P value adjustment: tukey method for comparing a family of 3 estimates
## Tests are performed on the log scale
```

```
#Model for Lactobacillus reuteri
sophie3.nbm <- glmer.nb(Lactobacillus_reuteri ~ SampleType*Breed  + SampleAge
+ (1|FarmID) + Period + AntibioticMilk + offset(log(TotalReads)),
                        data = mergedASV_24)
```

```
## boundary (singular) fit: see ?isSingular
```

```
sophie3.obj <- emmeans(sophie3.nbm, pairwise ~ SampleType | Breed, type= "res
ponse")
emmip(sophie3.nbm, ~ SampleType | Breed, type = "response", CIs = T) + theme_
bw() + ylab("Normalized Read Counts") +
  scale_y_log10() #to plot
```

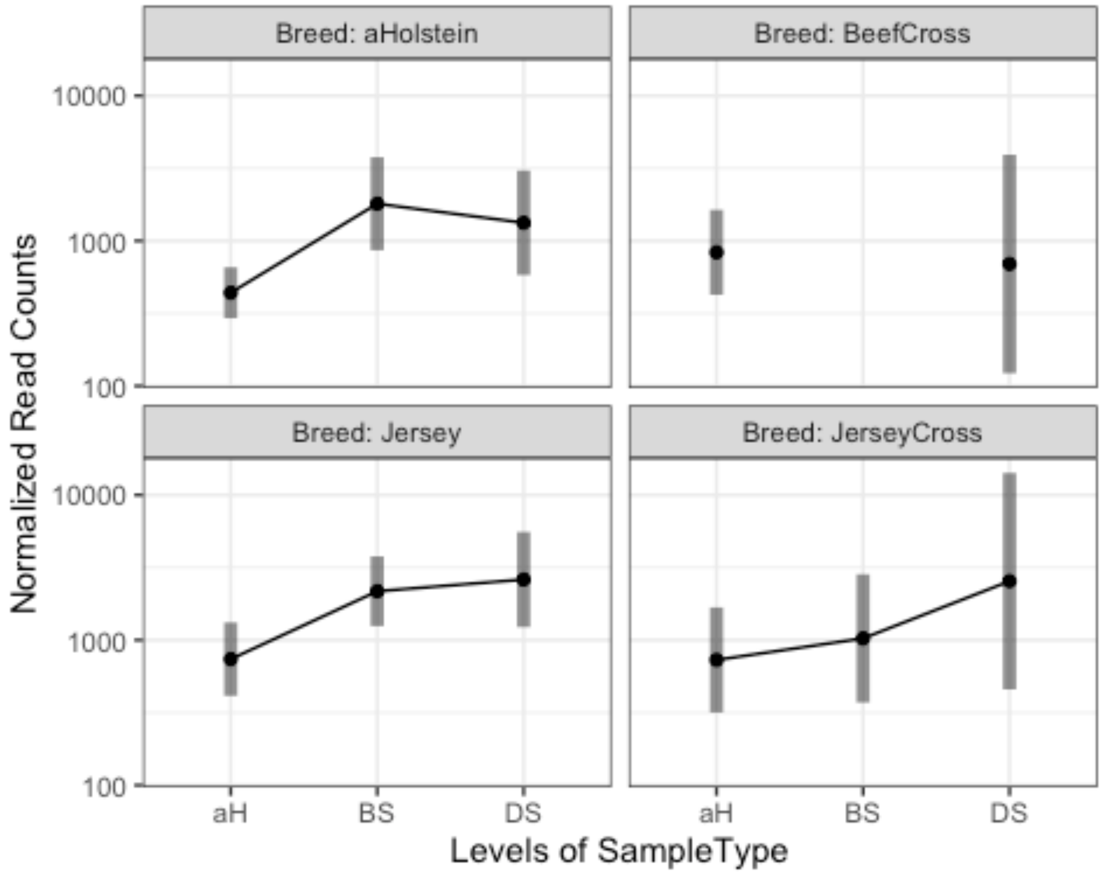


```
summary(sophie3.nbm)

## Generalized linear mixed model fit by maximum likelihood (Laplace
##   Approximation) [glmerMod]
##  Family: Negative Binomial(0.6719)  ( log )
## Formula: Lactobacillus_reuteri ~ SampleType * Breed + SampleAge + (1 |
##     FarmID) + Period + AntibioticMilk + offset(log(TotalReads))
##    Data: mergedASV_24
##
##      AIC      BIC   logLik deviance df.resid
##   2647.0   2696.9  -1307.5   2615.0      151
##
## Scaled residuals:
##     Min      1Q  Median      3Q     Max
## -0.8196 -0.6457 -0.2904  0.2624  3.4177
##
## Random effects:
##  Groups Name        Variance Std.Dev.
##  FarmID (Intercept) 1.26e-11 3.55e-06
## Number of obs: 167, groups:  FarmID, 2
##
## Fixed effects:
##                               Estimate Std. Error z value Pr(>|z|)
```

```
## (Intercept)                     -4.44673    0.68049  -6.535 6.38e-11 ***
## SampleTypeBS                     1.41273    0.41744   3.384 0.000714 ***
## SampleTypeDS                     1.11053    0.44155   2.515 0.011900 *
## BreedBeefCross                   0.64186    0.36434   1.762 0.078118 .
## BreedJersey                      0.52107    0.34839   1.496 0.134742
## BreedJerseyCross                 0.50862    0.44170   1.152 0.249515
## SampleAge                        0.10238    0.04292   2.385 0.017065 *
## Period                          -0.80906    0.24223  -3.340 0.000837 ***
## AntibioticMilkTRUE               0.70018    0.29071   2.409 0.016018 *
## SampleTypeDS:BreedBeefCross     -1.29591    1.04006  -1.246 0.212768
## SampleTypeBS:BreedJersey        -0.33472    0.55848  -0.599 0.548943
## SampleTypeDS:BreedJersey         0.15358    0.64282   0.239 0.811176
## SampleTypeBS:BreedJerseyCross -1.07079    0.75141  -1.425 0.154150
## SampleTypeDS:BreedJerseyCross  0.13999    1.06093   0.132 0.895024
## ---
## Signif. codes:  0 '***' 0.001 '**' 0.01 '*' 0.05 '.' 0.1 ' ' 1

##
## Correlation matrix not shown by default, as p = 14 > 12.
## Use print(x, correlation=TRUE)  or
##     vcov(x)        if you need it

## fit warnings:
## fixed-effect model matrix is rank deficient so dropping 1 column / coeffic
ient
## optimizer (Nelder_Mead) convergence code: 0 (OK)
## boundary (singular) fit: see ?isSingular
```

```
sophie3.obj #to get pvalues
```

```
## $emmeans
## Breed = aHolstein:
##  SampleType response     SE  df asymp.LCL asymp.UCL
##  aH              439   90.5 Inf       293       658
##  BS             1803  679.2 Inf       862      3773
##  DS             1333  564.1 Inf       581      3055
##
## Breed = BeefCross:
##  SampleType response     SE  df asymp.LCL asymp.UCL
##  aH              834  286.9 Inf       425      1637
##  BS           nonEst     NA  NA        NA        NA
##  DS              693  613.1 Inf       122      3924
##
## Breed = Jersey:
##  SampleType response     SE  df asymp.LCL asymp.UCL
##  aH              739  219.6 Inf       413      1323
##  BS             2172  613.1 Inf      1249      3777
##  DS             2617 1007.5 Inf      1230      5565
##
## Breed = JerseyCross:
##  SampleType response     SE  df asymp.LCL asymp.UCL
```

```
##   aH               730  310.6 Inf       317      1681
##   BS              1028  533.5 Inf       372      2843
##   DS              2550 2234.2 Inf       458     14204
##
## Results are averaged over the levels of: Period, AntibioticMilk
## Confidence level used: 0.95
## Intervals are back-transformed from the log scale
##
## $contrasts
## Breed = aHolstein:
##   contrast  ratio    SE  df z.ratio p.value
##   aH / BS   0.243 0.102 Inf -3.384  0.0021
##   aH / DS   0.329 0.145 Inf -2.515  0.0319
##   BS / DS   1.353 0.771 Inf  0.530  0.8565
##
## Breed = BeefCross:
##   contrast  ratio    SE  df z.ratio p.value
##   aH / BS  nonEst    NA  NA      NA      NA
##   aH / DS   1.204 1.126 Inf  0.198  0.9786
##   BS / DS  nonEst    NA  NA      NA      NA
##
## Breed = Jersey:
##   contrast  ratio    SE  df z.ratio p.value
##   aH / BS   0.340 0.130 Inf -2.813  0.0136
##   aH / DS   0.282 0.133 Inf -2.681  0.0201
##   BS / DS   0.830 0.389 Inf -0.398  0.9166
##
## Breed = JerseyCross:
##   contrast  ratio    SE  df z.ratio p.value
##   aH / BS   0.710 0.466 Inf -0.521  0.8609
##   aH / DS   0.286 0.275 Inf -1.300  0.3949
##   BS / DS   0.403 0.403 Inf -0.909  0.6346
##
## Results are averaged over the levels of: Period, AntibioticMilk
## P value adjustment: tukey method for comparing a family of 3 estimates
## Tests are performed on the log scale
```

```
#Model for Lactobacillus_salivarius
claire3.nbm <- glmer.nb(Lactobacillus_salivarius  ~ SampleType*Breed  + Sampl
eAge + Period + AntibioticMilk + (1|FarmID) + offset(log(TotalReads)),
                        data = mergedASV_24)
```

```
## boundary (singular) fit: see ?isSingular
```

```
claire3.obj <- emmeans(claire3.nbm, pairwise ~ SampleType | Breed, type= "res
ponse")
emmip(claire3.nbm, ~ SampleType | Breed, type = "response", CIs = T) + theme_
bw() + ylab("Normalized Read Counts") +
  scale_y_log10() #to plot
```

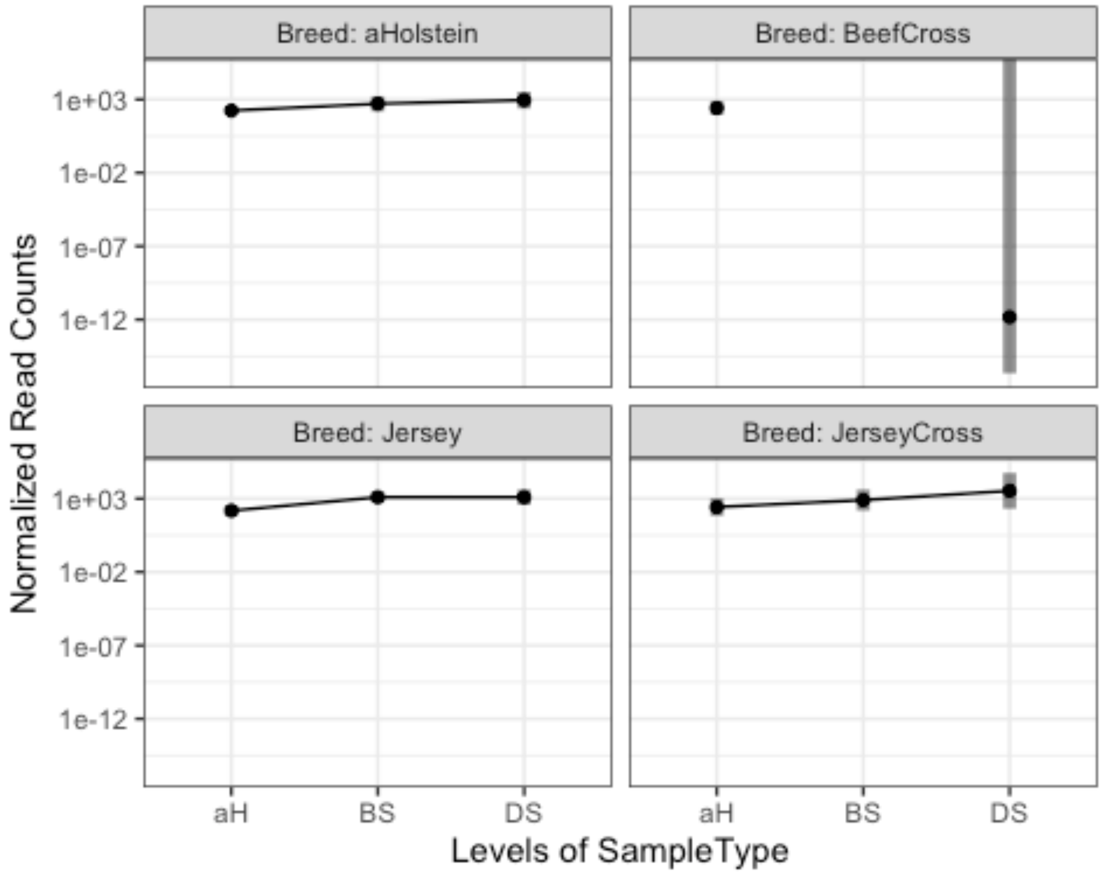


```
summary(claire3.nbm)

## Generalized linear mixed model fit by maximum likelihood (Laplace
##   Approximation) [glmerMod]
##  Family: Negative Binomial(0.2493)  ( log )
## Formula: Lactobacillus_salivarius ~ SampleType * Breed + SampleAge + Perio
d +
##     AntibioticMilk + (1 | FarmID) + offset(log(TotalReads))
##    Data: mergedASV_24
##
##      AIC      BIC   logLik deviance df.resid
##   2046.5   2096.4  -1007.3   2014.5      151
##
## Scaled residuals:
##     Min      1Q  Median      3Q     Max
## -0.4993 -0.4903 -0.3926  0.0127  4.8055
##
## Random effects:
##  Groups Name        Variance  Std.Dev.
##  FarmID (Intercept) 8.747e-11 9.352e-06
## Number of obs: 167, groups:  FarmID, 2
##
## Fixed effects:
```

```
##                                   Estimate Std. Error z value Pr(>|z|)
## (Intercept)                     -5.422e+00  1.113e+00  -4.874  1.1e-06 ***
## SampleTypeBS                     1.074e+00  6.581e-01   1.631   0.1028
## SampleTypeDS                     1.637e+00  7.336e-01   2.231   0.0257 *
## BreedBeefCross                   4.124e-01  5.905e-01   0.698   0.4849
## BreedJersey                     -1.356e-01  5.303e-01  -0.256   0.7982
## BreedJerseyCross                 4.334e-01  7.465e-01   0.581   0.5616
## SampleAge                        4.525e-02  6.313e-02   0.717   0.4735
## Period                          -4.430e-01  3.666e-01  -1.209   0.2268
## AntibioticMilkTRUE               7.630e-01  5.079e-01   1.502   0.1330
## SampleTypeDS:BreedBeefCross     -3.443e+01  4.962e+05   0.000   0.9999
## SampleTypeBS:BreedJersey         1.046e+00  8.905e-01   1.174   0.2402
## SampleTypeDS:BreedJersey         4.788e-01  1.047e+00   0.457   0.6475
## SampleTypeBS:BreedJerseyCross  2.131e-02  1.238e+00   0.017   0.9863
## SampleTypeDS:BreedJerseyCross  9.126e-01  1.731e+00   0.527   0.5981
## ---
## Signif. codes:  0 '***' 0.001 '**' 0.01 '*' 0.05 '.' 0.1 ' ' 1

##
## Correlation matrix not shown by default, as p = 14 > 12.
## Use print(x, correlation=TRUE)  or
##     vcov(x)        if you need it

## fit warnings:
## fixed-effect model matrix is rank deficient so dropping 1 column / coeffic
ient
## optimizer (Nelder_Mead) convergence code: 0 (OK)
## boundary (singular) fit: see ?isSingular
```

```
claire3.obj #to get pvalues
```

```
## $emmeans
## Breed = aHolstein:
##  SampleType response     SE  df asymp.LCL asymp.UCL
##  aH              172   58.3 Inf      88.5       334
##  BS              503  305.1 Inf     153.4      1651
##  DS              884  595.9 Inf     235.7      3313
##
## Breed = BeefCross:
##  SampleType response     SE  df asymp.LCL asymp.UCL
##  aH              260  147.6 Inf      85.4       791
##  BS           nonEst     NA  NA        NA        NA
##  DS                0    0.0 Inf       0.0       Inf
##
## Breed = Jersey:
##  SampleType response     SE  df asymp.LCL asymp.UCL
##  aH              150   67.5 Inf      62.3       362
##  BS             1251  577.5 Inf     505.9      3092
##  DS             1246  775.9 Inf     367.5      4223
##
## Breed = JerseyCross:
```

```
##  SampleType response     SE  df asymp.LCL asymp.UCL
##  aH              265  187.4 Inf      66.5      1059
##  BS              793  674.6 Inf     149.7      4201
##  DS             3395 4861.4 Inf     205.1     56191
##
## Results are averaged over the levels of: Period, AntibioticMilk
## Confidence level used: 0.95
## Intervals are back-transformed from the log scale
##
## $contrasts
## Breed = aHolstein:
##  contrast    ratio      SE  df z.ratio p.value
##  aH / BS  0.00e+00 0.0e+00 Inf -1.631  0.2324
##  aH / DS  0.00e+00 0.0e+00 Inf -2.231  0.0661
##  BS / DS  1.00e+00 1.0e+00 Inf -0.629  0.8040
##
## Breed = BeefCross:
##  contrast    ratio      SE  df z.ratio p.value
##  aH / BS    nonEst      NA  NA      NA      NA
##  aH / DS  1.75e+14 8.7e+19 Inf  0.000  1.0000
##  BS / DS    nonEst      NA  NA      NA      NA
##
## Breed = Jersey:
##  contrast    ratio      SE  df z.ratio p.value
##  aH / BS  0.00e+00 0.0e+00 Inf -3.454  0.0016
##  aH / DS  0.00e+00 0.0e+00 Inf -2.861  0.0117
##  BS / DS  1.00e+00 1.0e+00 Inf  0.005  1.0000
##
## Breed = JerseyCross:
##  contrast    ratio      SE  df z.ratio p.value
##  aH / BS  0.00e+00 0.0e+00 Inf -1.028  0.5594
##  aH / DS  0.00e+00 0.0e+00 Inf -1.622  0.2363
##  BS / DS  0.00e+00 0.0e+00 Inf -0.886  0.6490
##
## Results are averaged over the levels of: Period, AntibioticMilk
## P value adjustment: tukey method for comparing a family of 3 estimates
## Tests are performed on the log scale
```

```
#Model for Shigella_sonnei
paty3.nbm <- glmer.nb(Shigella_sonnei  ~ SampleType*Breed  + SampleAge + (1|F
armID) + Period + AntibioticMilk +offset(log(TotalReads)),
                      data = mergedASV_24)
```

```
## boundary (singular) fit: see ?isSingular
```

```
paty3.obj <- emmeans(paty3.nbm, pairwise ~ SampleType | Breed, type= "respons
e")
emmip(paty3.nbm, ~ SampleType | Breed, type = "response", CIs = T) + theme_bw
() + ylab("Normalized Read Counts") +
  scale_y_log10() #to plot
```

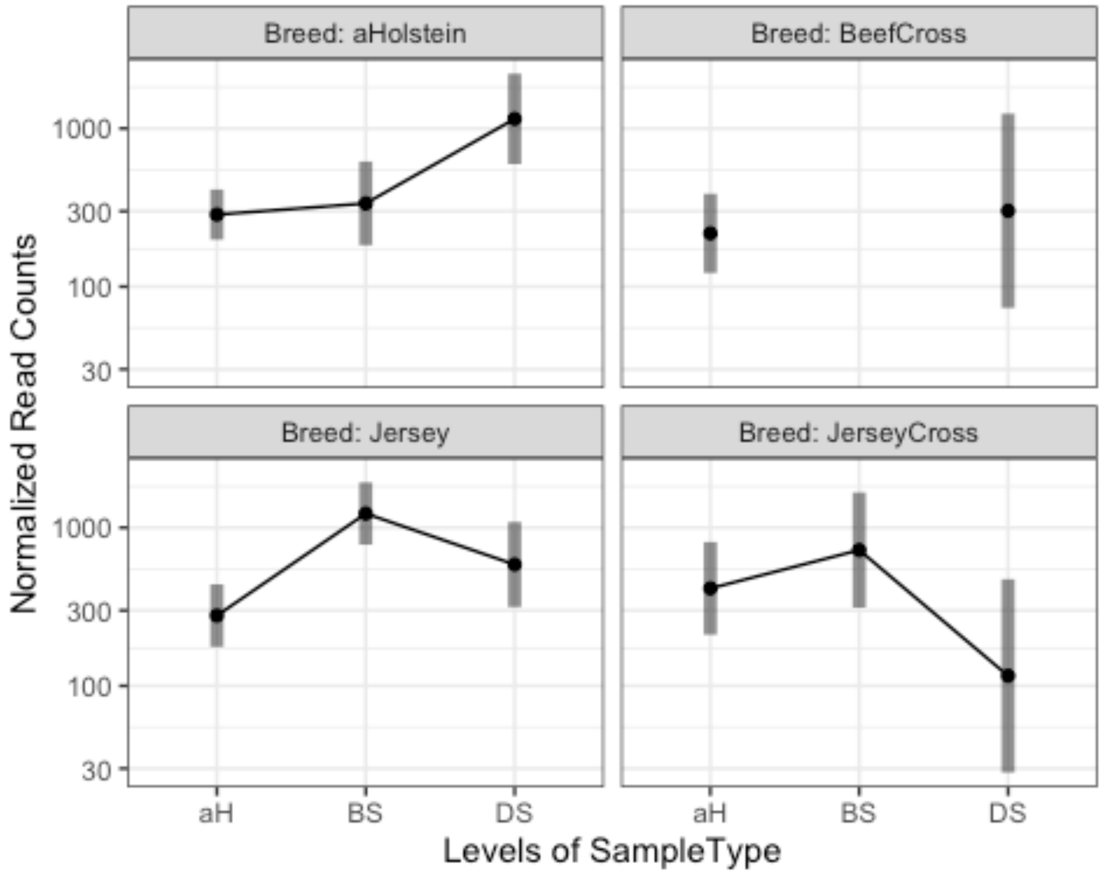


```
summary(paty3.nbm)

## Generalized linear mixed model fit by maximum likelihood (Laplace
##   Approximation) [glmerMod]
##  Family: Negative Binomial(1.0031)  ( log )
## Formula: Shigella_sonnei ~ SampleType * Breed + SampleAge + (1 | FarmID) +
##     Period + AntibioticMilk + offset(log(TotalReads))
##    Data: mergedASV_24
##
##      AIC      BIC   logLik deviance df.resid
##   2329.3   2379.1  -1148.6   2297.3      151
##
## Scaled residuals:
##     Min      1Q  Median      3Q     Max
## -0.9980 -0.6766 -0.2331  0.3061  6.3582
##
## Random effects:
##  Groups Name        Variance  Std.Dev.
##  FarmID (Intercept) 5.332e-12 2.309e-06
## Number of obs: 167, groups:  FarmID, 2
##
## Fixed effects:
##                               Estimate Std. Error z value Pr(>|z|)
```

```
## (Intercept)                      -3.26235    0.59921  -5.444 5.20e-08 ***
## SampleTypeBS                      0.16281    0.32714   0.498 0.618706
## SampleTypeDS                      1.39352    0.37114   3.755 0.000174 ***
## BreedBeefCross                   -0.27384    0.29817  -0.918 0.358400
## BreedJersey                      -0.02616    0.27122  -0.096 0.923159
## BreedJerseyCross                  0.37023    0.36847   1.005 0.315010
## SampleAge                        -0.15743    0.03605  -4.367 1.26e-05 ***
## Period                           -0.08002    0.18975  -0.422 0.673258
## AntibioticMilkTRUE                0.21556    0.29233   0.737 0.460898
## SampleTypeDS:BreedBeefCross      -1.06438    0.83874  -1.269 0.204436
## SampleTypeBS:BreedJersey          1.32355    0.45482   2.910 0.003613 **
## SampleTypeDS:BreedJersey         -0.64923    0.53116  -1.222 0.221598
## SampleTypeBS:BreedJerseyCross     0.39540    0.61603   0.642 0.520970
## SampleTypeDS:BreedJerseyCross    -2.66710    0.87070  -3.063 0.002190 **
## ---
## Signif. codes:  0 '***' 0.001 '**' 0.01 '*' 0.05 '.' 0.1 ' ' 1

##
## Correlation matrix not shown by default, as p = 14 > 12.
## Use print(x, correlation=TRUE)  or
##     vcov(x)        if you need it

## fit warnings:
## fixed-effect model matrix is rank deficient so dropping 1 column / coeffic
ient
## optimizer (Nelder_Mead) convergence code: 0 (OK)
## boundary (singular) fit: see ?isSingular
```

```
paty3.obj #to get pvalues
```

```
## $emmeans
## Breed = aHolstein:
##  SampleType response    SE  df asymp.LCL asymp.UCL
##  aH              286  53.2 Inf     198.2       411
##  BS              336 104.5 Inf     182.7       618
##  DS             1150 386.3 Inf     595.6      2222
##
## Breed = BeefCross:
##  SampleType response    SE  df asymp.LCL asymp.UCL
##  aH              217  63.9 Inf     122.0       386
##  BS           nonEst    NA  NA        NA        NA
##  DS              302 217.9 Inf      73.3      1242
##
## Breed = Jersey:
##  SampleType response    SE  df asymp.LCL asymp.UCL
##  aH              278  64.7 Inf     176.3       439
##  BS             1230 284.5 Inf     781.3      1935
##  DS              585 185.1 Inf     315.0      1088
##
## Breed = JerseyCross:
##  SampleType response    SE  df asymp.LCL asymp.UCL
```

```
##   aH                413 142.3 Inf     210.6       812
##   BS                723 309.4 Inf     312.2      1672
##   DS                116  83.1 Inf      28.3       473
## 
## Results are averaged over the levels of: Period, AntibioticMilk 
## Confidence level used: 0.95 
## Intervals are back-transformed from the log scale 
## 
## $contrasts
## Breed = aHolstein:
##   contrast  ratio     SE  df z.ratio p.value
##   aH / BS   0.850 0.2780 Inf -0.498  0.8724
##   aH / DS   0.248 0.0921 Inf -3.755  0.0005
##   BS / DS   0.292 0.1326 Inf -2.712  0.0184
## 
## Breed = BeefCross:
##   contrast  ratio     SE  df z.ratio p.value
##   aH / BS  nonEst     NA  NA      NA      NA
##   aH / DS   0.720 0.5419 Inf -0.437  0.9001
##   BS / DS  nonEst     NA  NA      NA      NA
## 
## Breed = Jersey:
##   contrast  ratio     SE  df z.ratio p.value
##   aH / BS   0.226 0.0706 Inf -4.759  <.0001
##   aH / DS   0.475 0.1738 Inf -2.034  0.1040
##   BS / DS   2.100 0.8071 Inf  1.931  0.1300
## 
## Breed = JerseyCross:
##   contrast  ratio     SE  df z.ratio p.value
##   aH / BS   0.572 0.3054 Inf -1.046  0.5478
##   aH / DS   3.574 2.8005 Inf  1.625  0.2350
##   BS / DS   6.245 5.1499 Inf  2.221  0.0676
## 
## Results are averaged over the levels of: Period, AntibioticMilk 
## P value adjustment: tukey method for comparing a family of 3 estimates 
## Tests are performed on the log scale
```

```
#Model for complex_1_Lactobacillus _ unclassified Lactobacillus
paula3.nbm <- glmer.nb(complex_1 ~ SampleType*Breed  + SampleAge + (1|FarmID) 
+ Period + AntibioticMilk + offset(log(TotalReads)),
                       data = mergedASV_24)

## boundary (singular) fit: see ?isSingular

paula3.obj <- emmeans(paula3.nbm, pairwise ~ SampleType | Breed, type= "respo
nse")
emmip(paula3.nbm, ~ SampleType | Breed, type = "response", CIs = T) + theme_b
w() + ylab("Normalized Read Counts") +
  scale_y_log10() #to plot
```

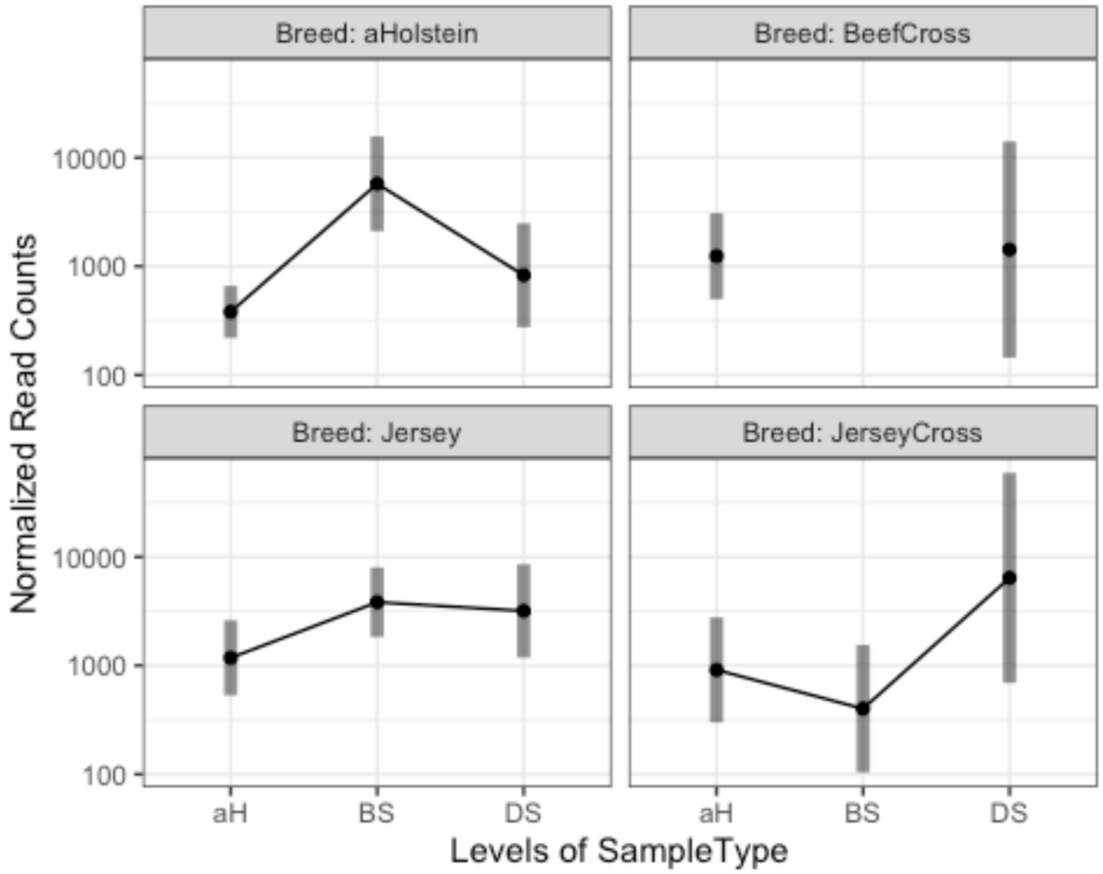


```
summary(paula3.nbm)

## Generalized linear mixed model fit by maximum likelihood (Laplace
##   Approximation) [glmerMod]
##  Family: Negative Binomial(0.3852)  ( log )
## Formula: complex_1 ~ SampleType * Breed + SampleAge + (1 | FarmID) + Perio
d +
##     AntibioticMilk + offset(log(TotalReads))
##    Data: mergedASV_24
##
##      AIC      BIC   logLik deviance df.resid
##   2643.4   2693.3  -1305.7   2611.4      151
##
## Scaled residuals:
##     Min      1Q  Median      3Q     Max
## -0.6206 -0.5779 -0.4176  0.1432  6.4951
##
## Random effects:
##  Groups Name        Variance  Std.Dev.
##  FarmID (Intercept) 5.171e-10 2.274e-05
## Number of obs: 167, groups:  FarmID, 2
##
## Fixed effects:
```

```
##                                  Estimate Std. Error z value Pr(>|z|)
## (Intercept)                      -4.84110    0.91783  -5.275 1.33e-07 ***
## SampleTypeBS                      2.71484    0.60071   4.519 6.20e-06 ***
## SampleTypeDS                      0.77588    0.58751   1.321 0.186627
## BreedBeefCross                    1.18007    0.47296   2.495 0.012592 *
## BreedJersey                       1.12871    0.48478   2.328 0.019897 *
## BreedJerseyCross                  0.87216    0.58153   1.500 0.133676
## SampleAge                         0.21431    0.05489   3.904 9.45e-05 ***
## Period                           -1.45503    0.35158  -4.139 3.49e-05 ***
## AntibioticMilkTRUE                1.01829    0.38817   2.623 0.008707 **
## SampleTypeDS:BreedBeefCross      -0.63553    1.36209  -0.467 0.640797
## SampleTypeBS:BreedJersey         -1.53576    0.77346  -1.986 0.047080 *
## SampleTypeDS:BreedJersey          0.21862    0.85656   0.255 0.798541
## SampleTypeBS:BreedJerseyCross -3.53598    1.00713  -3.511 0.000446 ***
## SampleTypeDS:BreedJerseyCross  1.17492    1.36368   0.862 0.388920
## ---
## Signif. codes:  0 '***' 0.001 '**' 0.01 '*' 0.05 '.' 0.1 ' ' 1

##
## Correlation matrix not shown by default, as p = 14 > 12.
## Use print(x, correlation=TRUE)  or
##     vcov(x)        if you need it

## fit warnings:
## fixed-effect model matrix is rank deficient so dropping 1 column / coeffic
ient
## optimizer (Nelder_Mead) convergence code: 0 (OK)
## boundary (singular) fit: see ?isSingular

paula3.obj #to get pvalues

## $emmeans
## Breed = aHolstein:
##  SampleType response   SE  df asymp.LCL asymp.UCL
##  aH              381  107 Inf       220       660
##  BS             5753 2951 Inf      2105     15723
##  DS              828  465 Inf       275      2490
##
## Breed = BeefCross:
##  SampleType response   SE  df asymp.LCL asymp.UCL
##  aH             1240  576 Inf       499      3080
##  BS           nonEst   NA  NA        NA        NA
##  DS             1427 1667 Inf       144     14100
##
## Breed = Jersey:
##  SampleType response   SE  df asymp.LCL asymp.UCL
##  aH             1178  478 Inf       531      2611
##  BS             3829 1440 Inf      1832      8003
##  DS             3184 1606 Inf      1184      8558
##
## Breed = JerseyCross:
```

```
##  SampleType response   SE  df asymp.LCL asymp.UCL
##  aH              911  517 Inf       300      2772
##  BS              401  277 Inf       104      1549
##  DS             6410 7275 Inf       693     59282
##
## Results are averaged over the levels of: Period, AntibioticMilk
## Confidence level used: 0.95
## Intervals are back-transformed from the log scale
##
## $contrasts
## Breed = aHolstein:
##  contrast  ratio     SE  df z.ratio p.value
##  aH / BS  0.0662 0.0398 Inf -4.519  <.0001
##  aH / DS  0.4603 0.2704 Inf -1.321  0.3836
##  BS / DS  6.9515 5.5425 Inf  2.432  0.0398
##
## Breed = BeefCross:
##  contrast  ratio     SE  df z.ratio p.value
##  aH / BS  nonEst     NA  NA      NA      NA
##  aH / DS  0.8691 1.0616 Inf -0.115  0.9927
##  BS / DS  nonEst     NA  NA      NA      NA
##
## Breed = Jersey:
##  contrast  ratio     SE  df z.ratio p.value
##  aH / BS  0.3076 0.1555 Inf -2.332  0.0515
##  aH / DS  0.3699 0.2329 Inf -1.580  0.2544
##  BS / DS  1.2027 0.7454 Inf  0.298  0.9523
##
## Breed = JerseyCross:
##  contrast  ratio     SE  df z.ratio p.value
##  aH / BS  2.2731 1.9594 Inf  0.953  0.6069
##  aH / DS  0.1422 0.1763 Inf -1.573  0.2575
##  BS / DS  0.0625 0.0821 Inf -2.111  0.0875
##
## Results are averaged over the levels of: Period, AntibioticMilk
## P value adjustment: tukey method for comparing a family of 3 estimates
## Tests are performed on the log scale
```

```
#Model for Streptococcus_gallolyticus
daisy3.nbm <- glmer.nb(Streptococcus_gallolyticus ~ SampleType*Breed  + Sampl
eAge + (1|FarmID) + Period + AntibioticMilk + offset(log(TotalReads)),
                       data = mergedASV_24)
```

```
## boundary (singular) fit: see ?isSingular
```

```
daisy3.obj <- emmeans(daisy3.nbm, pairwise ~ SampleType | Breed, type= "respo
nse")
emmip(daisy3.nbm, ~ SampleType | Breed, type = "response", CIs = T) + theme_b
w() + ylab("Normalized Read Counts") +
  scale_y_log10() #to plot
```

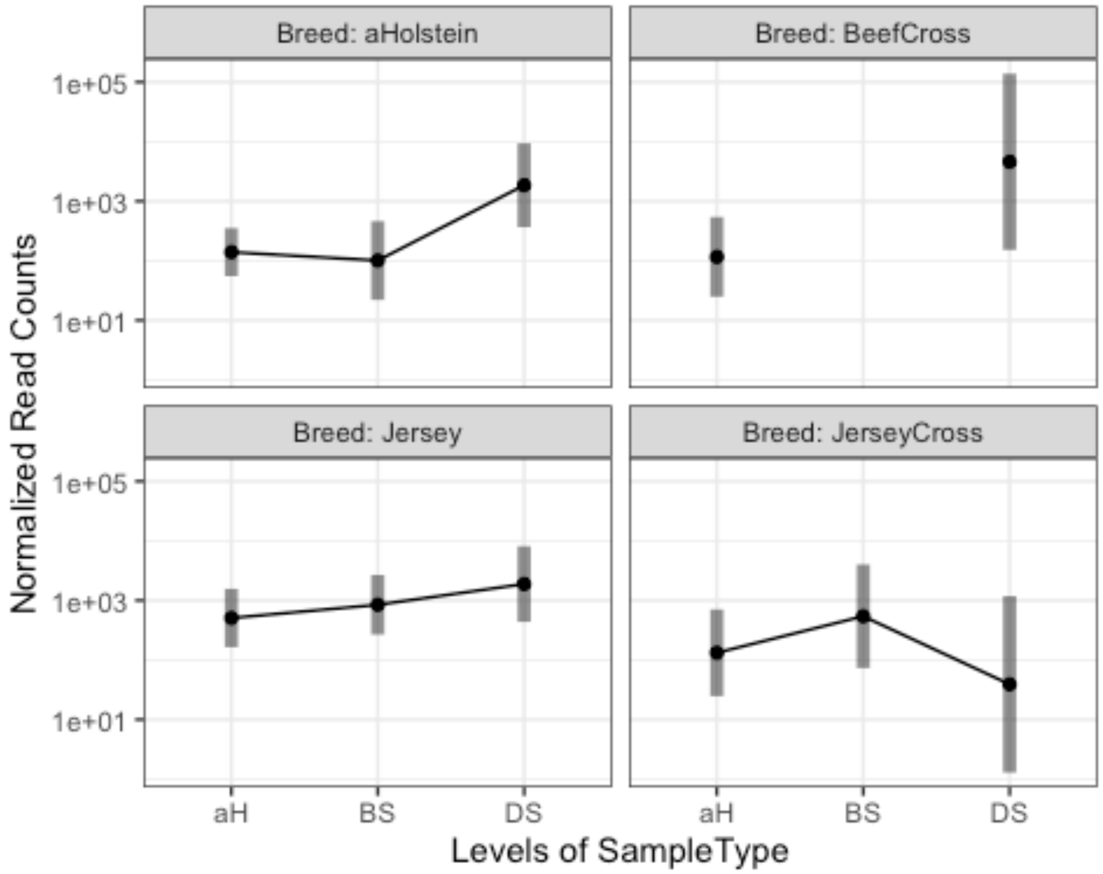


```
summary(daisy3.nbm)

## Generalized linear mixed model fit by maximum likelihood (Laplace
##   Approximation) [glmerMod]
##  Family: Negative Binomial(0.1726)  ( log )
## Formula: Streptococcus_gallolyticus ~ SampleType * Breed + SampleAge +
##     (1 | FarmID) + Period + AntibioticMilk + offset(log(TotalReads))
##    Data: mergedASV_24
##
##      AIC      BIC   logLik deviance df.resid
##   2017.4   2067.3   -992.7   1985.4      151
##
## Scaled residuals:
##     Min      1Q  Median      3Q     Max
## -0.4155 -0.4150 -0.3757 -0.0059 10.7536
##
## Random effects:
##  Groups Name        Variance Std.Dev.
##  FarmID (Intercept) 5.86e-13 7.655e-07
## Number of obs: 167, groups:  FarmID, 2
##
## Fixed effects:
##                                 Estimate Std. Error z value Pr(>|z|)
```

```
## (Intercept)                        -6.1098     1.6734  -3.651 0.000261 ***
## SampleTypeBS                       -0.3241     0.7586  -0.427 0.669169
## SampleTypeDS                        2.5857     0.8869   2.915 0.003552 **
## BreedBeefCross                     -0.1839     0.7263  -0.253 0.800172
## BreedJersey                         1.2894     0.6400   2.015 0.043934 *
## BreedJerseyCross                   -0.0554     0.9506  -0.058 0.953532
## SampleAge                           0.1293     0.1227   1.054 0.291785
## Period                             -1.1913     0.5015  -2.375 0.017529 *
## AntibioticMilkTRUE                  2.3706     0.7713   3.074 0.002115 **
## SampleTypeDS:BreedBeefCross         1.0894     2.0694   0.526 0.598584
## SampleTypeBS:BreedJersey            0.8360     1.0717   0.780 0.435349
## SampleTypeDS:BreedJersey           -1.2724     1.2649  -1.006 0.314433
## SampleTypeBS:BreedJerseyCross       1.7364     1.4877   1.167 0.243163
## SampleTypeDS:BreedJerseyCross      -3.8036     2.0938  -1.817 0.069273 .
## ---
## Signif. codes:  0 '***' 0.001 '**' 0.01 '*' 0.05 '.' 0.1 ' ' 1

##
## Correlation matrix not shown by default, as p = 14 > 12.
## Use print(x, correlation=TRUE)  or
##     vcov(x)        if you need it

## fit warnings:
## fixed-effect model matrix is rank deficient so dropping 1 column / coeffic
ient
## optimizer (Nelder_Mead) convergence code: 0 (OK)
## boundary (singular) fit: see ?isSingular

daisy3.obj #to get pvalues

## $emmeans
## Breed = aHolstein:
##  SampleType response     SE  df asymp.LCL asymp.UCL
##  aH            139.7   66.2 Inf     55.16       354
##  BS            101.0   78.4 Inf     22.05       463
##  DS           1853.8 1533.2 Inf    366.50      9377
##
## Breed = BeefCross:
##  SampleType response     SE  df asymp.LCL asymp.UCL
##  aH            116.2   91.3 Inf     24.91       542
##  BS           nonEst     NA  NA        NA        NA
##  DS           4584.9 7968.1 Inf    152.06    138243
##
## Breed = Jersey:
##  SampleType response     SE  df asymp.LCL asymp.UCL
##  aH            507.1  291.8 Inf    164.16      1566
##  BS            846.0  497.2 Inf    267.34      2677
##  DS           1885.5 1406.0 Inf    437.19      8131
##
## Breed = JerseyCross:
##  SampleType response     SE  df asymp.LCL asymp.UCL
```

```
##  aH             132.1  113.0 Inf     24.71       707
##  BS             542.4  554.0 Inf     73.28      4015
##  DS              39.1   68.0 Inf      1.29      1182
##
## Results are averaged over the levels of: Period, AntibioticMilk
## Confidence level used: 0.95
## Intervals are back-transformed from the log scale
##
## $contrasts
## Breed = aHolstein:
##  contrast   ratio      SE  df z.ratio p.value
##  aH / BS   1.3828  1.0490 Inf  0.427  0.9043
##  aH / DS   0.0753  0.0668 Inf -2.915  0.0099
##  BS / DS   0.0545  0.0592 Inf -2.676  0.0204
##
## Breed = BeefCross:
##  contrast   ratio      SE  df z.ratio p.value
##  aH / BS   nonEst      NA  NA      NA      NA
##  aH / DS   0.0253  0.0471 Inf -1.979  0.1173
##  BS / DS   nonEst      NA  NA      NA      NA
##
## Breed = Jersey:
##  contrast   ratio      SE  df z.ratio p.value
##  aH / BS   0.5994  0.4654 Inf -0.659  0.7871
##  aH / DS   0.2689  0.2424 Inf -1.457  0.3119
##  BS / DS   0.4487  0.3905 Inf -0.921  0.6271
##
## Breed = JerseyCross:
##  contrast   ratio      SE  df z.ratio p.value
##  aH / BS   0.2436  0.3109 Inf -1.107  0.5100
##  aH / DS   3.3799  6.4112 Inf  0.642  0.7969
##  BS / DS  13.8747 27.3767 Inf  1.333  0.3768
##
## Results are averaged over the levels of: Period, AntibioticMilk
## P value adjustment: tukey method for comparing a family of 3 estimates
## Tests are performed on the log scale
```

```
 #Model for Faecalibacterium prausnitzii
jenny2.nbm <- glmer.nb(Faecalibacterium_prausnitzii ~ SampleType*Breed  + Sam
pleAge + Period + AntibioticMilk + (1|FarmID) + offset(log(TotalReads)),
                      data = mergedASV_24)

## boundary (singular) fit: see ?isSingular

jenny2.obj <- emmeans(jenny2.nbm, pairwise ~ SampleType | Breed, type= "respo
nse")
emmip(jenny2.nbm, ~ SampleType | Breed, type = "response", CIs = T) + theme_b
w() + ylab("Normalized Read Counts") +
  scale_y_log10(lim = c(1,1e4)) #to plot
```

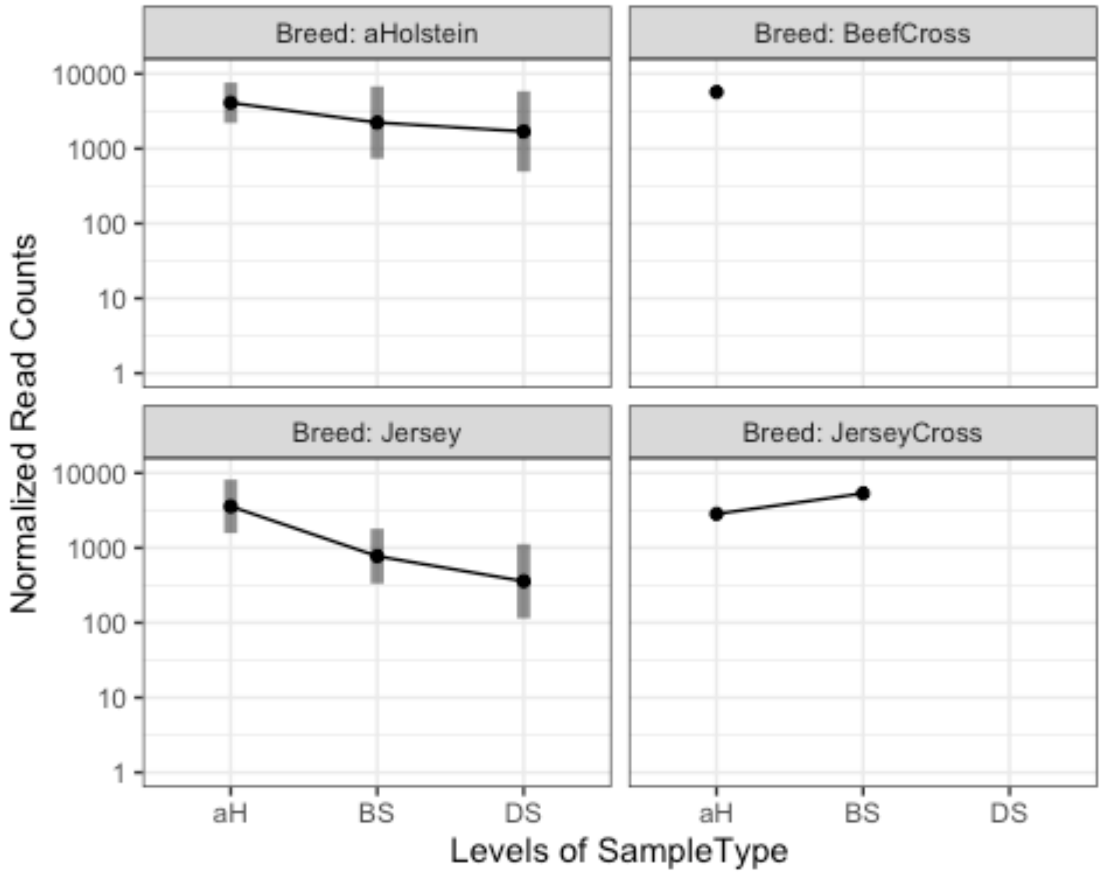


```
summary(jenny2.nbm)

## Generalized linear mixed model fit by maximum likelihood (Laplace
##   Approximation) [glmerMod]
##  Family: Negative Binomial(0.285)  ( log )
## Formula: Faecalibacterium_prausnitzii ~ SampleType * Breed + SampleAge +
##     Period + AntibioticMilk + (1 | FarmID) + offset(log(TotalReads))
##    Data: mergedASV_24
##
##      AIC      BIC   logLik deviance df.resid
##   2464.8   2514.7  -1216.4   2432.8      151
##
## Scaled residuals:
##     Min      1Q  Median      3Q     Max
## -0.5339 -0.5235 -0.2826  0.2525  3.6757
##
## Random effects:
##  Groups Name        Variance  Std.Dev.
##  FarmID (Intercept) 5.186e-11 7.201e-06
## Number of obs: 167, groups:  FarmID, 2
##
## Fixed effects:
##                                 Estimate Std. Error z value Pr(>|z|)
```

```
## (Intercept)                        -0.2404     1.0417  -0.231  0.81749
## SampleTypeBS                       -0.6081     0.6155  -0.988  0.32313
## SampleTypeDS                       -0.8821     0.6859  -1.286  0.19846
## BreedBeefCross                      0.3286     0.5521   0.595  0.55179
## BreedJersey                        -0.1342     0.4958  -0.271  0.78662
## BreedJerseyCross                   -0.3651     0.6983  -0.523  0.60107
## SampleAge                          -0.1868     0.0590  -3.167  0.00154 **
## Period                              0.4120     0.3456   1.192  0.23319
## AntibioticMilkTRUE                 -1.4225     0.4746  -2.998  0.00272 **
## SampleTypeDS:BreedBeefCross       -27.5750 19404.8910  -0.001  0.99887
## SampleTypeBS:BreedJersey           -0.9239     0.8327  -1.110  0.26720
## SampleTypeDS:BreedJersey           -1.4197     0.9796  -1.449  0.14724
## SampleTypeBS:BreedJerseyCross       1.2375     1.1575   1.069  0.28501
## SampleTypeDS:BreedJerseyCross     -26.8763 17884.9668  -0.002  0.99880
## ---
## Signif. codes:  0 '***' 0.001 '**' 0.01 '*' 0.05 '.' 0.1 ' ' 1

##
## Correlation matrix not shown by default, as p = 14 > 12.
## Use print(x, correlation=TRUE)  or
##     vcov(x)        if you need it

## fit warnings:
## fixed-effect model matrix is rank deficient so dropping 1 column / coeffic
ient
## optimizer (Nelder_Mead) convergence code: 0 (OK)
## boundary (singular) fit: see ?isSingular
```

```
jenny2.obj #to get pvalues
```

```
## $emmeans
## Breed = aHolstein:
##  SampleType response   SE  df asymp.LCL asymp.UCL
##  aH             4090 1296 Inf      2198      7609
##  BS             2226 1262 Inf       733      6761
##  DS             1693 1068 Inf       492      5826
##
## Breed = BeefCross:
##  SampleType response   SE  df asymp.LCL asymp.UCL
##  aH             5680 3019 Inf      2005     16097
##  BS           nonEst   NA  NA        NA        NA
##  DS                0    0 Inf         0       Inf
##
## Breed = Jersey:
##  SampleType response   SE  df asymp.LCL asymp.UCL
##  aH             3576 1501 Inf      1570      8143
##  BS              773  334 Inf       331      1802
##  DS              358  209 Inf       114      1122
##
## Breed = JerseyCross:
##  SampleType response   SE  df asymp.LCL asymp.UCL
```

```
##   aH              2839 1874 Inf       778     10354
##   BS              5327 4237 Inf      1120     25323
##   DS                 0    0 Inf         0       Inf
##
## Results are averaged over the levels of: Period, AntibioticMilk
## Confidence level used: 0.95
## Intervals are back-transformed from the log scale
##
## $contrasts
## Breed = aHolstein:
##  contrast    ratio       SE  df z.ratio p.value
##  aH / BS  2.00e+00 1.00e+00 Inf   0.988  0.5844
##  aH / DS  2.00e+00 2.00e+00 Inf   1.286  0.4031
##  BS / DS  1.00e+00 1.00e+00 Inf   0.327  0.9426
##
## Breed = BeefCross:
##  contrast    ratio       SE  df z.ratio p.value
##  aH / BS    nonEst       NA  NA      NA      NA
##  aH / DS  2.28e+12 4.43e+16 Inf   0.001  1.0000
##  BS / DS    nonEst       NA  NA      NA      NA
##
## Breed = Jersey:
##  contrast    ratio       SE  df z.ratio p.value
##  aH / BS  5.00e+00 3.00e+00 Inf   2.671  0.0207
##  aH / DS  1.00e+01 7.00e+00 Inf   3.328  0.0025
##  BS / DS  2.00e+00 2.00e+00 Inf   1.097  0.5158
##
## Breed = JerseyCross:
##  contrast    ratio       SE  df z.ratio p.value
##  aH / BS  1.00e+00 1.00e+00 Inf -0.632  0.8026
##  aH / DS  1.14e+12 2.03e+16 Inf   0.002  1.0000
##  BS / DS  2.13e+12 3.81e+16 Inf   0.002  1.0000
##
## Results are averaged over the levels of: Period, AntibioticMilk
## P value adjustment: tukey method for comparing a family of 3 estimates
## Tests are performed on the log scale
```

```
#Model for Collinsella aerofaciens
hope2.nbm <- glmer.nb(Collinsella_aerofaciens ~ SampleType*Breed  + SampleAge
+ Period + AntibioticMilk + (1|FarmID) + offset(log(TotalReads)),
                      data = mergedASV_24)
```

```
## boundary (singular) fit: see ?isSingular
```

```
hope2.obj <- emmeans(hope2.nbm, pairwise ~ SampleType | Breed, type= "respons
e")
emmip(hope2.nbm, ~ SampleType | Breed, type = "response", CIs = T) + theme_bw
() + ylab("Normalized Read Counts") +
  scale_y_log10(lim = c(1,1e4)) #to plot
```

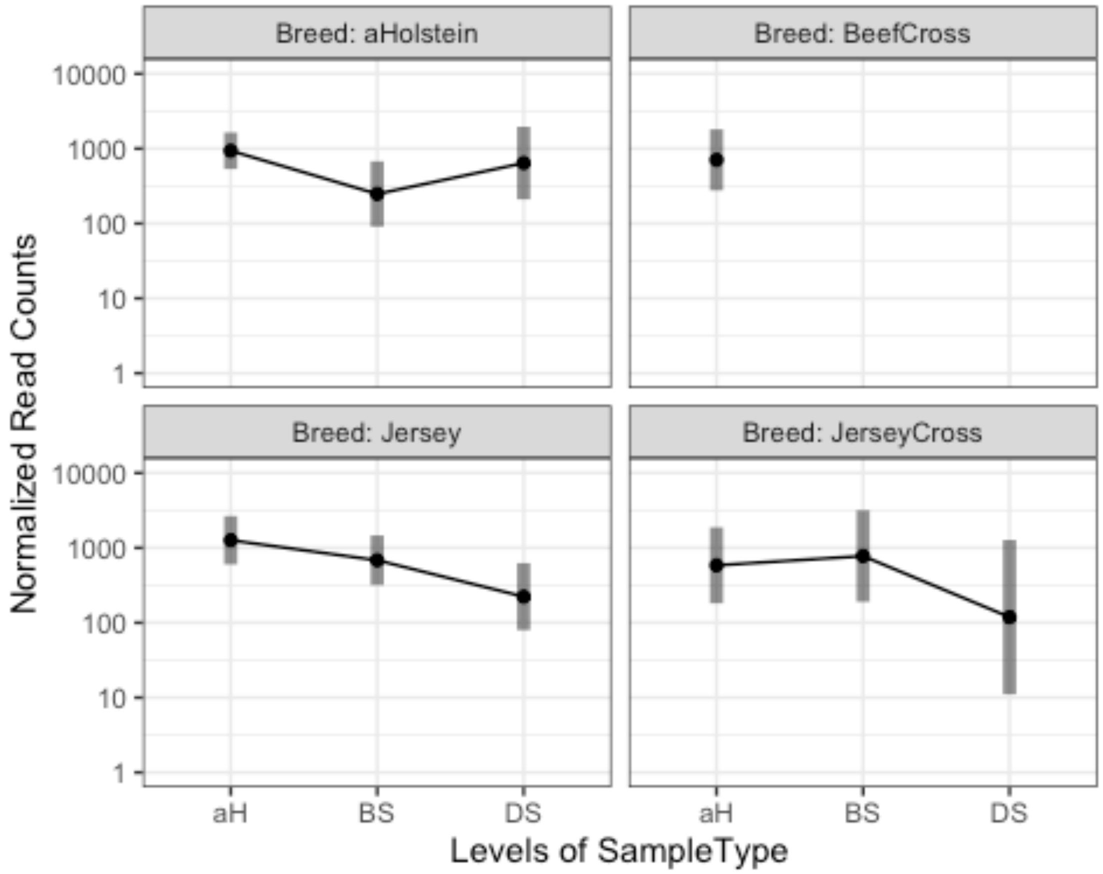


```
summary(hope2.nbm)

## Generalized linear mixed model fit by maximum likelihood (Laplace
##   Approximation) [glmerMod]
##  Family: Negative Binomial(0.3498)  ( log )
## Formula: Collinsella_aerofaciens ~ SampleType * Breed + SampleAge + Period
+
##     AntibioticMilk + (1 | FarmID) + offset(log(TotalReads))
##    Data: mergedASV_24
##
##      AIC      BIC   logLik deviance df.resid
##   2418.7   2468.6  -1193.4   2386.7      151
##
## Scaled residuals:
##     Min      1Q  Median      3Q     Max
## -0.5914 -0.5208 -0.2470  0.2848  3.1981
##
## Random effects:
##  Groups Name        Variance  Std.Dev.
##  FarmID (Intercept) 3.184e-09 5.643e-05
## Number of obs: 167, groups:  FarmID, 2
##
## Fixed effects:
```

```
##                                  Estimate Std. Error z value Pr(>|z|)
## (Intercept)                    -5.197e+00  9.393e-01  -5.533 3.15e-08 ***
## SampleTypeBS                   -1.329e+00  5.558e-01  -2.391  0.01679 *
## SampleTypeDS                   -3.781e-01  6.194e-01  -0.610  0.54161
## BreedBeefCross                 -2.811e-01  4.984e-01  -0.564  0.57282
## BreedJersey                     2.998e-01  4.474e-01   0.670  0.50273
## BreedJerseyCross               -4.708e-01  6.301e-01  -0.747  0.45500
## SampleAge                       1.623e-01  5.328e-02   3.046  0.00232 **
## Period                         -2.566e-01  3.094e-01  -0.829  0.40693
## AntibioticMilkTRUE              9.185e-01  4.286e-01   2.143  0.03212 *
## SampleTypeDS:BreedBeefCross    -3.737e+01  4.165e+06   0.000  0.99999
## SampleTypeBS:BreedJersey        7.132e-01  7.519e-01   0.949  0.34284
## SampleTypeDS:BreedJersey       -1.356e+00  8.842e-01  -1.534  0.12515
## SampleTypeBS:BreedJerseyCross   1.614e+00  1.045e+00   1.544  0.12261
## SampleTypeDS:BreedJerseyCross -1.215e+00  1.463e+00  -0.830  0.40630
## ---
## Signif. codes:  0 '***' 0.001 '**' 0.01 '*' 0.05 '.' 0.1 ' ' 1

##
## Correlation matrix not shown by default, as p = 14 > 12.
## Use print(x, correlation=TRUE)  or
##     vcov(x)        if you need it

## fit warnings:
## fixed-effect model matrix is rank deficient so dropping 1 column / coeffic
ient
## optimizer (Nelder_Mead) convergence code: 0 (OK)
## boundary (singular) fit: see ?isSingular
```

```
hope2.obj #to get pvalues
```

```
## $emmeans
## Breed = aHolstein:
##  SampleType response  SE  df asymp.LCL asymp.UCL
##  aH              935 267 Inf     534.0      1638
##  BS              248 127 Inf      90.8       675
##  DS              641 365 Inf     209.9      1956
##
## Breed = BeefCross:
##  SampleType response  SE  df asymp.LCL asymp.UCL
##  aH              706 339 Inf     275.9      1807
##  BS           nonEst  NA  NA        NA        NA
##  DS                0   0 Inf       0.0       Inf
##
## Breed = Jersey:
##  SampleType response  SE  df asymp.LCL asymp.UCL
##  aH             1262 478 Inf     600.4      2653
##  BS              682 266 Inf     317.5      1464
##  DS              223 117 Inf      79.5       625
##
## Breed = JerseyCross:
```

```
##  SampleType response  SE  df asymp.LCL asymp.UCL
##  aH              584 348 Inf     181.6      1878
##  BS              777 558 Inf     190.0      3174
##  DS              119 144 Inf      11.1      1274
##
## Results are averaged over the levels of: Period, AntibioticMilk
## Confidence level used: 0.95
## Intervals are back-transformed from the log scale
##
## $contrasts
## Breed = aHolstein:
##  contrast    ratio       SE  df z.ratio p.value
##  aH / BS  4.00e+00 2.00e+00 Inf  2.391  0.0443
##  aH / DS  1.00e+00 1.00e+00 Inf  0.610  0.8144
##  BS / DS  0.00e+00 0.00e+00 Inf -1.259  0.4187
##
## Breed = BeefCross:
##  contrast    ratio       SE  df z.ratio p.value
##  aH / BS    nonEst       NA  NA      NA      NA
##  aH / DS  2.47e+16 1.03e+23 Inf  0.000  1.0000
##  BS / DS    nonEst       NA  NA      NA      NA
##
## Breed = Jersey:
##  contrast    ratio       SE  df z.ratio p.value
##  aH / BS  2.00e+00 1.00e+00 Inf  1.189  0.4597
##  aH / DS  6.00e+00 4.00e+00 Inf  2.778  0.0151
##  BS / DS  3.00e+00 2.00e+00 Inf  1.766  0.1810
##
## Breed = JerseyCross:
##  contrast    ratio       SE  df z.ratio p.value
##  aH / BS  1.00e+00 1.00e+00 Inf -0.317  0.9463
##  aH / DS  5.00e+00 7.00e+00 Inf  1.199  0.4536
##  BS / DS  7.00e+00 9.00e+00 Inf  1.354  0.3654
##
## Results are averaged over the levels of: Period, AntibioticMilk
## P value adjustment: tukey method for comparing a family of 3 estimates
## Tests are performed on the log scale
```

```
#Model for Bacteroides vulgatus
amy2.nbm <- glmer.nb(Bacteroides_vulgatus    ~ SampleType*Breed  + SampleAge
+ Period + AntibioticMilk + (1|FarmID) + offset(log(TotalReads)),
                    data = mergedASV_24)

## boundary (singular) fit: see ?isSingular

amy2.obj <- emmeans(amy2.nbm, pairwise ~ SampleType | Breed, type= "response"
)
emmip(amy2.nbm, ~ SampleType | Breed, type = "response", CIs = T) + theme_bw(
) + ylab("Normalized Read Counts") +
  scale_y_log10() #to plot
```

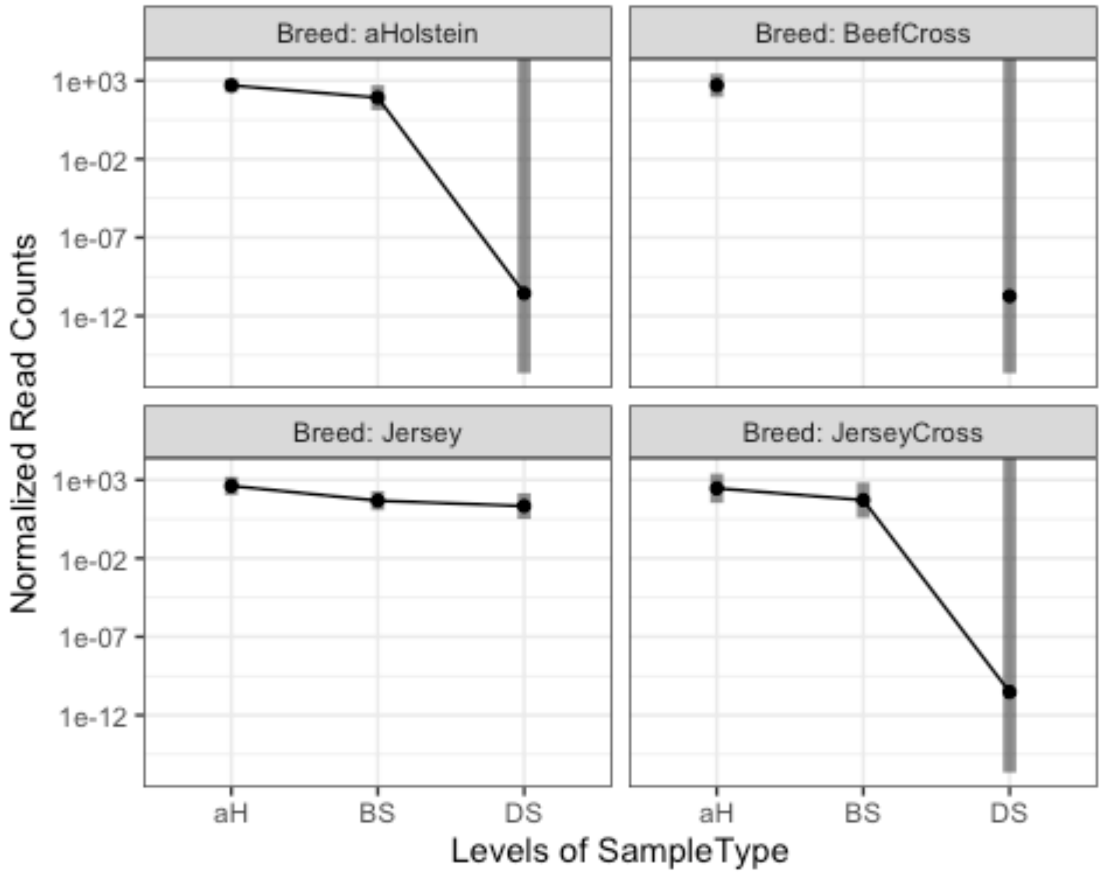


```
summary(amy2.nbm)

## Generalized linear mixed model fit by maximum likelihood (Laplace
##   Approximation) [glmerMod]
##  Family: Negative Binomial(0.1053)  ( log )
## Formula: Bacteroides_vulgatus ~ SampleType * Breed + SampleAge + Period +
##     AntibioticMilk + (1 | FarmID) + offset(log(TotalReads))
##    Data: mergedASV_24
##
##      AIC      BIC   logLik deviance df.resid
##   1325.6   1375.5   -646.8   1293.6      151
##
## Scaled residuals:
##     Min      1Q  Median      3Q     Max
## -0.3244 -0.3243 -0.3181  0.0000  4.7428
##
## Random effects:
##  Groups Name        Variance  Std.Dev.
##  FarmID (Intercept) 1.408e-12 1.187e-06
## Number of obs: 167, groups:  FarmID, 2
##
## Fixed effects:
##                                 Estimate Std. Error z value Pr(>|z|)
```

```
## (Intercept)                     -3.090e+00  1.840e+00  -1.680   0.0930 .
## SampleTypeBS                    -1.791e+00  1.016e+00  -1.763   0.0779 .
## SampleTypeDS                    -3.052e+01  5.127e+04  -0.001   0.9995
## BreedBeefCross                   4.166e-03  9.103e-01   0.005   0.9963
## BreedJersey                     -1.832e-01  8.181e-01  -0.224   0.8228
## BreedJerseyCross                -5.286e-01  1.153e+00  -0.458   0.6466
## SampleAge                        5.359e-02  1.024e-01   0.524   0.6006
## Period                          -8.843e-01  5.908e-01  -1.497   0.1345
## AntibioticMilkTRUE              -6.218e-01  8.321e-01  -0.747   0.4549
## SampleTypeDS:BreedBeefCross     -4.206e-01  1.884e+05   0.000   1.0000
## SampleTypeBS:BreedJersey        -3.725e-01  1.373e+00  -0.271   0.7861
## SampleTypeDS:BreedJersey         2.757e+01  5.127e+04   0.001   0.9996
## SampleTypeBS:BreedJerseyCross    6.084e-02  1.910e+00   0.032   0.9746
## SampleTypeDS:BreedJerseyCross    6.120e-01  1.500e+05   0.000   1.0000
## ---
## Signif. codes:  0 '***' 0.001 '**' 0.01 '*' 0.05 '.' 0.1 ' ' 1

##
## Correlation matrix not shown by default, as p = 14 > 12.
## Use print(x, correlation=TRUE)  or
##     vcov(x)        if you need it

## fit warnings:
## fixed-effect model matrix is rank deficient so dropping 1 column / coeffic
ient
## optimizer (Nelder_Mead) convergence code: 0 (OK)
## boundary (singular) fit: see ?isSingular

amy2.obj #to get pvalues

## $emmeans
## Breed = aHolstein:
##  SampleType response    SE  df asymp.LCL asymp.UCL
##  aH            494.7 262.0 Inf    175.19      1397
##  BS             82.6  77.5 Inf     13.10       520
##  DS              0.0   0.0 Inf      0.00       Inf
##
## Breed = BeefCross:
##  SampleType response    SE  df asymp.LCL asymp.UCL
##  aH            496.8 436.1 Inf     88.89      2776
##  BS           nonEst    NA  NA        NA        NA
##  DS              0.0   0.0 Inf      0.00       Inf
##
## Breed = Jersey:
##  SampleType response    SE  df asymp.LCL asymp.UCL
##  aH            411.9 286.8 Inf    105.22      1612
##  BS             47.4  34.0 Inf     11.60       193
##  DS             21.5  20.8 Inf      3.24       143
##
## Breed = JerseyCross:
##  SampleType response    SE  df asymp.LCL asymp.UCL
```

```
##  aH             291.6 319.7 Inf      34.00      2501
##  BS              51.7  68.3 Inf       3.88       689
##  DS               0.0   0.0 Inf       0.00       Inf
##
## Results are averaged over the levels of: Period, AntibioticMilk
## Confidence level used: 0.95
## Intervals are back-transformed from the log scale
##
## $contrasts
## Breed = aHolstein:
##  contrast    ratio       SE  df z.ratio p.value
##  aH / BS  6.00e+00 6.00e+00 Inf 1.763   0.1823
##  aH / DS  1.80e+13 9.20e+17 Inf 0.001   1.0000
##  BS / DS  3.00e+12 1.54e+17 Inf 0.001   1.0000
##
## Breed = BeefCross:
##  contrast    ratio       SE  df z.ratio p.value
##  aH / BS    nonEst       NA  NA     NA      NA
##  aH / DS  2.73e+13 4.96e+18 Inf 0.000   1.0000
##  BS / DS    nonEst       NA  NA     NA      NA
##
## Breed = Jersey:
##  contrast    ratio       SE  df z.ratio p.value
##  aH / BS  9.00e+00 8.00e+00 Inf 2.288   0.0575
##  aH / DS  1.90e+01 2.20e+01 Inf 2.586   0.0263
##  BS / DS  2.00e+00 3.00e+00 Inf 0.681   0.7746
##
## Breed = JerseyCross:
##  contrast    ratio       SE  df z.ratio p.value
##  aH / BS  6.00e+00 9.00e+00 Inf 1.051   0.5445
##  aH / DS  9.73e+12 1.37e+18 Inf 0.000   1.0000
##  BS / DS  1.73e+12 2.43e+17 Inf 0.000   1.0000
##
## Results are averaged over the levels of: Period, AntibioticMilk
## P value adjustment: tukey method for comparing a family of 3 estimates
## Tests are performed on the log scale

#Model for Bacteroides_fragilis
dale2.nbm <- glmer.nb(Bacteroides_fragilis  ~ SampleType*Breed  + SampleAge +
(1|FarmID) + Period + AntibioticMilk + offset(log(TotalReads)),
                     data = mergedASV_24)

## boundary (singular) fit: see ?isSingular

dale2.obj <- emmeans(dale2.nbm, pairwise ~ SampleType | Breed, type= "respons
e")
emmip(dale2.nbm, ~ SampleType | Breed, type = "response", CIs = T) + theme_bw
() + ylab("Normalized Read Counts") +
  scale_y_log10() #to plot
```

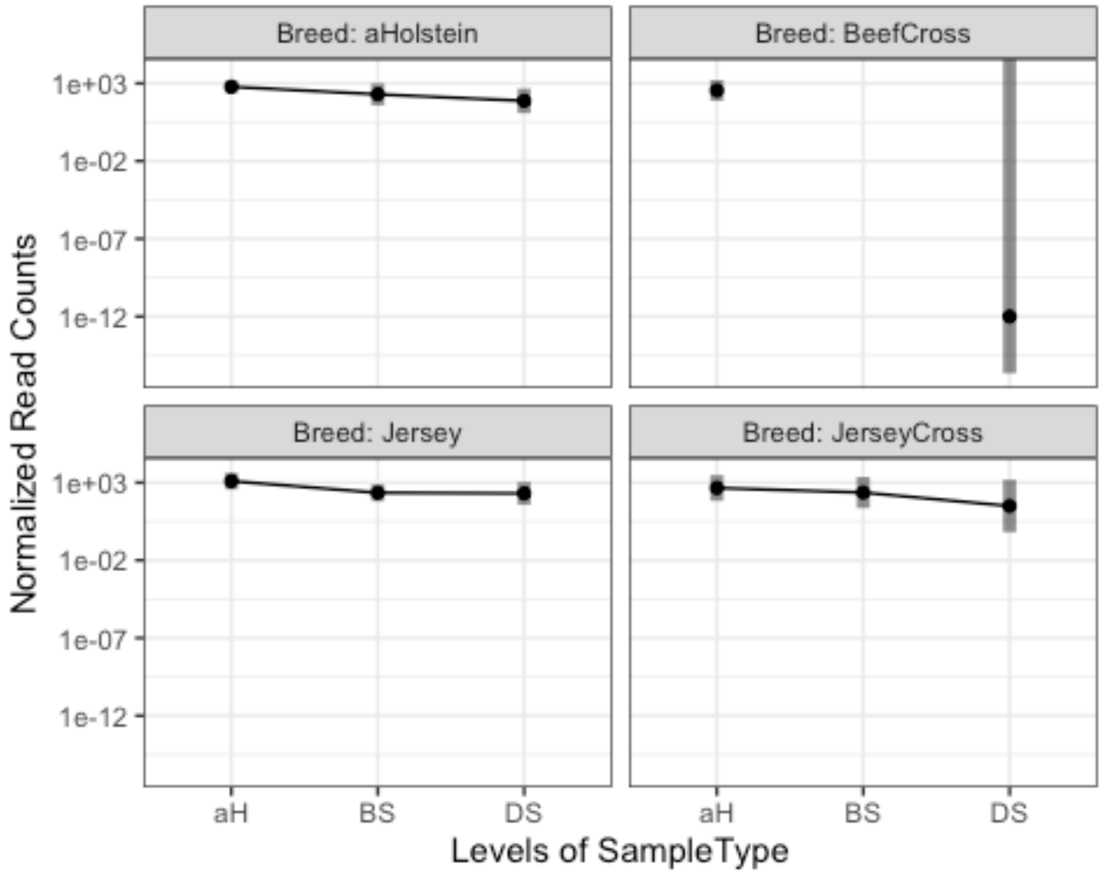


```
summary(dale2.nbm)

## Generalized linear mixed model fit by maximum likelihood (Laplace
##   Approximation) [glmerMod]
##  Family: Negative Binomial(0.131)  ( log )
## Formula: Bacteroides_fragilis ~ SampleType * Breed + SampleAge + (1 |
##     FarmID) + Period + AntibioticMilk + offset(log(TotalReads))
##    Data: mergedASV_24
##
##      AIC      BIC   logLik deviance df.resid
##   1644.1   1694.0   -806.1   1612.1      151
##
## Scaled residuals:
##     Min      1Q  Median      3Q     Max
## -0.3619 -0.3617 -0.3432 -0.0370  4.9374
##
## Random effects:
##  Groups Name        Variance  Std.Dev.
##  FarmID (Intercept) 1.371e-09 3.703e-05
## Number of obs: 167, groups:  FarmID, 2
##
## Fixed effects:
##                                   Estimate Std. Error z value Pr(>|z|)
```

```
## (Intercept)                      -1.113e+00  1.535e+00  -0.725   0.4683
## SampleTypeBS                     -1.119e+00  9.080e-01  -1.233   0.2177
## SampleTypeDS                     -2.086e+00  1.013e+00  -2.059   0.0395 *
## BreedBeefCross                   -5.463e-01  8.145e-01  -0.671   0.5024
## BreedJersey                       7.256e-01  7.311e-01   0.992   0.3210
## BreedJerseyCross                 -2.880e-01  1.030e+00  -0.280   0.7797
## SampleAge                        -2.075e-01  8.707e-02  -2.384   0.0171 *
## Period                           -4.641e-01  5.057e-01  -0.918   0.3587
## AntibioticMilkTRUE               -4.879e-01  7.003e-01  -0.697   0.4860
## SampleTypeDS:BreedBeefCross      -3.140e+01  6.002e+05   0.000   1.0000
## SampleTypeBS:BreedJersey         -6.070e-01  1.229e+00  -0.494   0.6212
## SampleTypeDS:BreedJersey          2.665e-01  1.445e+00   0.184   0.8537
## SampleTypeBS:BreedJerseyCross     4.425e-01  1.708e+00   0.259   0.7956
## SampleTypeDS:BreedJerseyCross -5.897e-01  2.393e+00  -0.246   0.8053
## ---
## Signif. codes:  0 '***' 0.001 '**' 0.01 '*' 0.05 '.' 0.1 ' ' 1

##
## Correlation matrix not shown by default, as p = 14 > 12.
## Use print(x, correlation=TRUE)  or
##     vcov(x)        if you need it

## fit warnings:
## fixed-effect model matrix is rank deficient so dropping 1 column / coeffic
ient
## optimizer (Nelder_Mead) convergence code: 0 (OK)
## boundary (singular) fit: see ?isSingular
```

```
dale2.obj #to get pvalues
```

```
## $emmeans
## Breed = aHolstein:
##  SampleType response    SE  df asymp.LCL asymp.UCL
##  aH            602.0 281.3 Inf   240.917      1504
##  BS            196.5 164.4 Inf    38.152      1013
##  DS             74.8  69.6 Inf    12.056       464
##
## Breed = BeefCross:
##  SampleType response    SE  df asymp.LCL asymp.UCL
##  aH            348.6 273.1 Inf    75.062      1619
##  BS           nonEst    NA  NA        NA        NA
##  DS              0.0   0.0 Inf     0.000       Inf
##
## Breed = Jersey:
##  SampleType response    SE  df asymp.LCL asymp.UCL
##  aH           1243.7 770.4 Inf   369.390      4187
##  BS            221.3 141.0 Inf    63.460       772
##  DS            201.7 173.4 Inf    37.420      1087
##
## Breed = JerseyCross:
##  SampleType response    SE  df asymp.LCL asymp.UCL
```

```
##   aH             451.3 439.6 Inf    66.901      3045
##   BS             229.4 269.3 Inf    22.979      2290
##   DS              31.1  61.6 Inf     0.641      1507
##
## Results are averaged over the levels of: Period, AntibioticMilk
## Confidence level used: 0.95
## Intervals are back-transformed from the log scale
##
## $contrasts
## Breed = aHolstein:
##  contrast    ratio      SE  df z.ratio p.value
##  aH / BS  3.00e+00 3.0e+00 Inf 1.233   0.4338
##  aH / DS  8.00e+00 8.0e+00 Inf 2.059   0.0985
##  BS / DS  3.00e+00 3.0e+00 Inf 0.782   0.7140
##
## Breed = BeefCross:
##  contrast    ratio      SE  df z.ratio p.value
##  aH / BS    nonEst      NA  NA     NA       NA
##  aH / DS  3.49e+14 2.1e+20 Inf 0.000   1.0000
##  BS / DS    nonEst      NA  NA     NA       NA
##
## Breed = Jersey:
##  contrast    ratio      SE  df z.ratio p.value
##  aH / BS  6.00e+00 5.0e+00 Inf 2.040   0.1028
##  aH / DS  6.00e+00 6.0e+00 Inf 1.783   0.1751
##  BS / DS  1.00e+00 1.0e+00 Inf 0.090   0.9956
##
## Breed = JerseyCross:
##  contrast    ratio      SE  df z.ratio p.value
##  aH / BS  2.00e+00 3.0e+00 Inf 0.460   0.8898
##  aH / DS  1.50e+01 3.2e+01 Inf 1.231   0.4346
##  BS / DS  7.00e+00 1.7e+01 Inf 0.881   0.6523
##
## Results are averaged over the levels of: Period, AntibioticMilk
## P value adjustment: tukey method for comparing a family of 3 estimates
## Tests are performed on the log scale
```

Prediction of disease

Disease prediction model

```
library("glmnet")
library("compositions")
library("tidyverse")
library("readxl")

mergedASV_21<- read_excel("mergedASV_21.xlsx")
```

```r
linmod <- formula(paste(paste("~",paste(names(mergedASV_21[c(2:58,60:62)]), c
ollapse = "+"),sep=""), "- 1"))
mergedASV2 <- mergedASV_21
mergedASV2[,2:58] <- mergedASV2[,2:58] / mergedASV2$TotalReads
mm <- model.matrix(linmod, mergedASV2)
regmod <- glmnet(mm, mergedASV2$Sick, family = "binomial")
regmod.cv <- cv.glmnet(mm, mergedASV2$Sick, family = "binomial")
coef(regmod.cv, s = "lambda.min")
```

```
## 64 x 1 sparse Matrix of class "dgCMatrix"
##                                             1
## (Intercept)                         0.1499450
## Bifidobacterium_longum             -1.0701368
## complex_1                           .
## Escherichia_coli                    8.5669651
## Lactobacillus_johnsonii             .
## Faecalibacterium_prausnitzii       -1.4413436
## Lactobacillus_reuteri               8.4050925
## Streptococcus_gallolyticus          1.0680583
## Tyzzerella_nexilis                  2.4727046
## Butyricicoccus_pullicaecorum       -3.7271108
## Collinsella_aerofaciens            -8.2071587
## Ruminococcus_torques                .
## Ruminococcus_gnavus                 .
## Lactobacillus_salivarius            8.3600234
## Shigella_sonnei                     .
## Bacteroides_fragilis               -5.0321852
## Bacteroides_vulgatus               -9.7210343
## Faecalicoccus_pleomorphus          -2.4482050
## Erysipelatoclostridium_ramosum      .
## Natranaerovirga_hydrolytica         .
## Lachnoclostridium_urinimassiliense  .
## Pseudoflavonifractor_capillosus     .
## Lachnoclostridium_pacaense        -60.7213975
## Flavonifractor_plautii             26.7816244
## Eggerthella_lenta                -900.2945773
## Howardella_ureilytica               .
## Roseimarinus_sediminis              .
## Bacteroides_sartorii                .
## Prevotella_baroniae                 .
## Odoribacter_splanchnicus            .
## Olsenella_profusa                   .
## Butyricimonas_virosa                .
## Pedobacter_alluvionis               .
## Stoquefichus_massiliensis           .
## Anaerotruncus_rubiinfantis          .
## Acetobacteroides_hydrogenigenes     .
## Anaerophaga_thermohalophila         .
## Alistipes_ihumii                    .
## Desulfovibrio_piger                 .
```

```
## Bacteroides_faecichinchillae          .
## Mycoplasma_arginini                   .
## Faecalitalea_cylindroides             .
## Anaerosolibacter_carboniphilus        .
## Bacillus_cereus                       .
## Robinsoniella_peoriensis              .
## Pedobacter_arcticus                   .
## Oscillibacter_ruminantium             .
## Collinsella_intestinalis              .
## Butyricicoccus_desmolans              .
## Eubacterium_sulci                     .
## Prevotella_conceptionensis            .
## Eubacterium_nodatum                   .
## Treponema_succinifaciens              .
## Holdemania_filiformis             1985.4126043
## Phocea_massiliensis                   .
## Mogibacterium_neglectum               .
## Ethanoligenens_harbinense             .
## Sporanaerobacter_acetigenes           .
## BreedBeefCross                      -0.3574345
## BreedHolstein                         .
## BreedJersey                          0.3817031
## BreedJerseyCross                      .
## SampleAge                           -0.1134314
## FarmIDTRUE                            .
```

```
minmod <- as.matrix(coef(regmod.cv, s = "lambda.1se") * c(1,apply(mm, 2, sd))
)
minmod[minmod !=0,]

#S3 Table - Coefficients of predictors of GI disease in calves.
##                   (Intercept)          Bifidobacterium_longum
##                  -0.824578700                    -0.225297894
##              Escherichia_coli           Lactobacillus_reuteri
##                   0.598952951                     0.391967328
##      Streptococcus_gallolyticus              Tyzzerella_nexilis
##                   0.087333520                     0.065588894
##         Collinsella_aerofaciens        Lactobacillus_salivarius
##                  -0.202218305                     0.273688586
##            Bacteroides_fragilis             Bacteroides_vulgatus
##                  -0.064688709                    -0.075184391
##       Faecalicoccus_pleomorphus Pseudoflavonifractor_capillosus
##                  -0.005167921                    -0.017057505
##      Lachnoclostridium_pacaense                Eggerthella_lenta
##                  -0.063097546                    -0.496730679
##                    BreedJersey                       SampleAge
##                   0.100018952                    -0.043157772

regmod.cv$cvm[which(regmod.cv$lambda == regmod.cv$lambda.1se)]

## [1] 0.9229986
```

```r
plot(regmod.cv) # S2 Fig - Model improvement with different numbers of parame
ters.
```

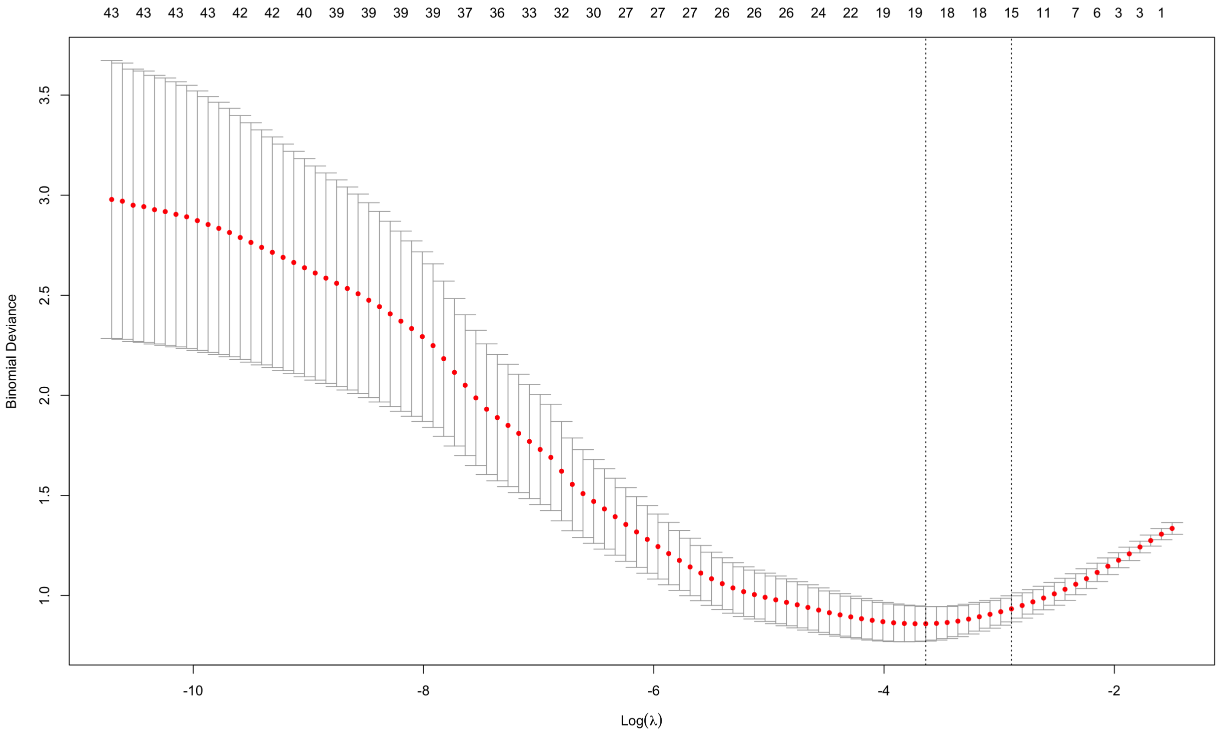

Supplement: S1 Appendix — (PDF) [file pone.0262317.s005.pdf]
